# A stabilized glycomimetic conjugate vaccine inducing protective antibodies against *Neisseria meningitidis* serogroup A

**DOI:** 10.1038/s41467-020-18279-x

**Published:** 2020-09-07

**Authors:** Jacopo Enotarpi, Marta Tontini, Cristiana Balocchi, Daan van der Es, Ludovic Auberger, Evita Balducci, Filippo Carboni, Daniela Proietti, Daniele Casini, Dmitri V. Filippov, Hermen S. Overkleeft, Gijsbert A. van der Marel, Cinzia Colombo, Maria Rosaria Romano, Francesco Berti, Paolo Costantino, Jeroen D. C. Codeé, Luigi Lay, Roberto Adamo

**Affiliations:** 1 Department of Chemistry and CRC Polymeric Materials (LaMPo), University of Milan, Milan, Italy; 2 Department of Bioorganic Synthesis, Leiden University, 2333 Leiden, The Netherlands; 3 GSK, Via Fiorentina 1, 53100 Siena, Italy

**Keywords:** Glycoconjugates, Lead optimization, Conjugate vaccines

## Abstract

*Neisseria meningitidis* serogroup A capsular polysaccharide (MenA CPS) consists of (1 → 6)-2-acetamido-2-deoxy-α-D-mannopyranosyl phosphate repeating units, *O*-acetylated at position C3 or C4. Glycomimetics appear attractive to overcome the CPS intrinsic lability in physiological media, due to cleavage of the phosphodiester bridge, and to develop a stable vaccine with longer shelf life in liquid formulation. Here, we generate a series of non-acetylated carbaMenA oligomers which are proven more stable than the CPS. An octamer (DP8) inhibits the binding of a MenA specific bactericidal mAb and polyclonal serum to the CPS, and is selected for further in vivo testing. However, its CRM_197_ conjugate raises murine antibodies towards the non-acetylated CPS backbone, but not the natural acetylated form. Accordingly, random *O*-acetylation of the DP8 is performed, resulting in a structure (Ac-carbaMenA) showing improved inhibition of anti-MenA CPS antibody binding and, after conjugation to CRM_197_, eliciting anti-MenA protective murine antibodies, comparably to the vaccine benchmark.

## Abstract (web summary)

The *Neisseria meningitidis* serogroup A capsular polysaccharide (MenA CPS) is a component of commercial vaccines, but is unstable. Here, the authors generate glycomimetic oligomers that demonstrate higher stability than their natural counterparts and induce protective antibodies in mice.

## Introduction

Vaccination, one of the most important advancements of modern medicine, is considered the most cost-effective strategy to protect the population from infectious diseases by the WHO^1^. Although well-established vaccines are commonly and successfully employed against a large number of diseases, the increased occurrence of antimicrobial resistance has raised the need for new and more efficient vaccines to prevent infections. In order to increase safety margins and improve efficacy of traditional vaccines, based on live-attenuated or whole-cell killed microorganisms, effort has been directed to the development of subunit vaccines, in which only a purified microbial component is administered. Amongst the subunit vaccines, carbohydrate-based vaccines have been applied to a variety of bacterial infections (*Haemophilus influenzae* type b, *Streptococcus pneumoniae*, *Neisseria meningitidis* and others)^2^. The vast majority of pathogens exposes a dense, often highly conserved array of glycan structures on their surface that exert a protective function against the host’s immune defense and are essential for their pathogenicity. Therefore, they represent attractive targets for vaccine design^3^. Typical examples are capsular polysaccharides (CPS) of encapsulated bacteria and lipopolysaccharides of Gram-negative bacteria. A major drawback of polysaccharide-based vaccines is their limited clinical efficacy. In fact, they are poorly immunogenic in infants, young children and immunocompromised patients. They do not induce immunological memory and their response is therefore not boosted by subsequent immunizations^4–6^. This derives from the fact that carbohydrates raise a T cell-independent immune response, hallmarked by the production of low affinity IgM antibodies and no conventional B cell-mediated immunological memory. Conjugation of CPS to a carrier protein, such as tetanus or diphtheria toxoids (TT, DT), or the nontoxic mutant of diphtheria toxin (Cross-Reacting Material 197, CRM_197_), leads to glycoconjugates with T cell-dependent properties^7^. Glycan-protein antigens enable memory B cell proliferation, ensuring long-lasting protection of the host. These features combined with the extensive clinical experience render glycoconjugates amongst the safest and most efficacious vaccines developed so far, and their generation has been one of the greatest success stories in the biomedical sciences^4,8,9^.

**Fig. 1:**
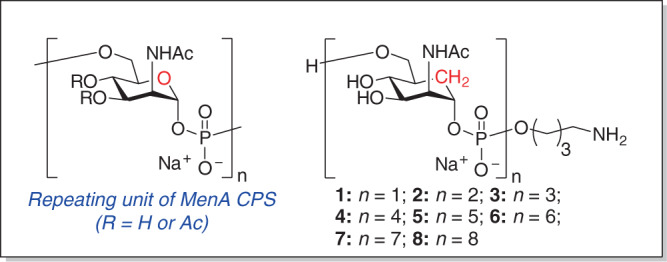
Structure of MenA CPS and carbaMenA. **a** Native CPS repeating unit and **b** target MenA carba analogs.

The Gram-negative encapsulated bacterium *N. meningitidis* is a major cause of bacterial meningitis occurring beyond the neonatal period. Six out of thirteen serotypes (A, C, B, W, Y, and X) are responsible of more than 90% of the reported infections^10^. Particularly, serogroup A (MenA) causes epidemic meningococcal disease in developing countries, predominantly throughout what is known as the African meningitis belt, with over 250 million of at-risk population^11,12^. The recent introduction of a MenA capsular polysaccharide-conjugate vaccine has dramatically decreased the disease incidence^13,14^, but the development and manufacture of an efficient glycoconjugate vaccine in fully liquid, shelf stable formulation, which would avoid the reconstitution of the lyophilized formulation before injection, remains a great challenge. The MenA capsular polysaccharide (MenA CPS) consists of (1 → 6)-linked 2-acetamido-2-deoxy-α-D-mannopyranosyl phosphate repeating units (Fig. 1)^15,16^. It has been shown that isolated MenA polysaccharide carries acetyl esters primarily at its C3-hydroxyl (80%) with limited acetylation of the C4-hydroxyl (10%). Recent studies however on meningococcal cells by high-resolution magic angle spinning NMR (HRMAS) spectroscopy have revealed a substitution at the C3-OH of 50-60% and at the C4-OH of 25–30%^15,17^. Like other phosphodiester containing polysaccharides, MenA is known to be poorly hydrolytically stable^18^. The intrinsic instability of MenA CPS is enhanced by the axial orientation of the acetamido functionality at C-2, which assists the facile cleavage of the anomeric phosphodiester linkage in aqueous environment^19,20^. The faster kinetic of hydrolysis of MenA polysaccharide compared to other antigens is well established^19^. A recent report suggests that a MenA-TT glycoconjugate is more stable to pH and temperature variation compared to the unconjugated polysaccharide, but this work only evaluated the activity of the conjugate in terms of antigenicity and not immunogenicity, and the effect of (partial) degradation on human immunization is unpredictable^21^. Generally, stability issues can be controlled by storage at 2–8 °C or overcome by freeze-drying. However, the availability of stable antigens, independent from the efficiency of the cold chain used for vaccine distribution, and with the potential of longer shelf life in liquid formulation is extremely attractive^22^.

Much effort is currently devoted to the generation of synthetic carbohydrate vaccines, in which chemically made oligosaccharides are used as antigens in conjugate vaccines^23,24^. The use of synthetic material has many advantages, including well-defined size, minimal (if any) batch-to-batch variation, and controlled conjugation chemistry. Indeed, synthetic carbohydrate vaccines have reached the market. In all these efforts fragments of the natural polysaccharides are copied through organic synthesis. Strategies for the chemical or enzymatic synthesis of natural MenA structures have been proposed^25,26^. Differently from these approaches, we envisaged the use of synthetic chemistry to generate stabilized MenA glycomimetics^27,28^. Mimics, in which structural changes are implemented to improve specific features of the natural polysaccharide have so far not been incorporated in conjugate vaccines inducing protective immune responses^29^. Given the exquisite recognition of antigens by antibodies it is a grand challenge to develop mimetics that sufficiently mimic their natural counterpart so that they can raise antibodies against the parent bacterium. We and others have explored the stabilization of the CPS in different ways. MenA CPS analogs in which the anomeric oxygen of the interglycosidic phosphodiester bond is replaced with a methylene group to obtain nonhydrolysable *C*-phosphono analogs of MenA CPS oligomers have been reported and mimics, in which the ring-oxygen is replaced by a methylene group, have also been conceived^30,31^. We previously generated short oligomers of these carbaMenA analogs, containing up to three repeating units (Fig. 1)^32^. We showed that IgG antibodies could be raised using a protein conjugate of the carbaMenA oligomer with a degree of polymerization of 3 (i.e., with three repeating units, DP3) that were capable of recognizing the native MenA CPS^33^. However, the carbaMenA DP3 showed poor potential in inhibiting the binding of anti-MenA CPS antibodies with the capsule, indicating the trimer to be a relatively poor synthetic antigen. Previous work delivering “natural” synthetic MenA fragments has shown that short oligos (4 repeating units) can be recognized by anti-MenA antibodies but the minimal epitope capable of inducing a strong immune response in immunization experiments remains to be determined^26^. To improve the efficacy of the carbaMenA antigens, here we describe the generation of longer oligomers, assembled using phosphoramidite chemistry. To this end oligomers up to 8 repeating units in length are evaluated as potential synthetic antigens (Fig. 1). CarbaMenA DP8 proves to be a lead antigen, capable of binding antibodies raised against the natural polysaccharide as well as to a functional monoclonal antibody. However, its CRM_197_ conjugate elicits in vivo antibodies recognizing the deacetylated polysaccharide but not the natural MenA capsule. Introduction of acetyl esters in the carbaMenA DP8 glycomimetic is demonstrated crucial to improve the MenA polysaccharide mimicry^34,35^, as the CRM_197_ conjugate of the Ac-carbaMenA DP8 leads to a high level of functional anti-MenA CPS antibodies, equal to the MenA vaccine benchmark. This work thus presents a proof-of-principle glycomimetic vaccine with activity that matches the conjugate based on the natural polysaccharide.

## Results

### Chemical synthesis of the nonacetylated carba analogs

**Fig. 2:**
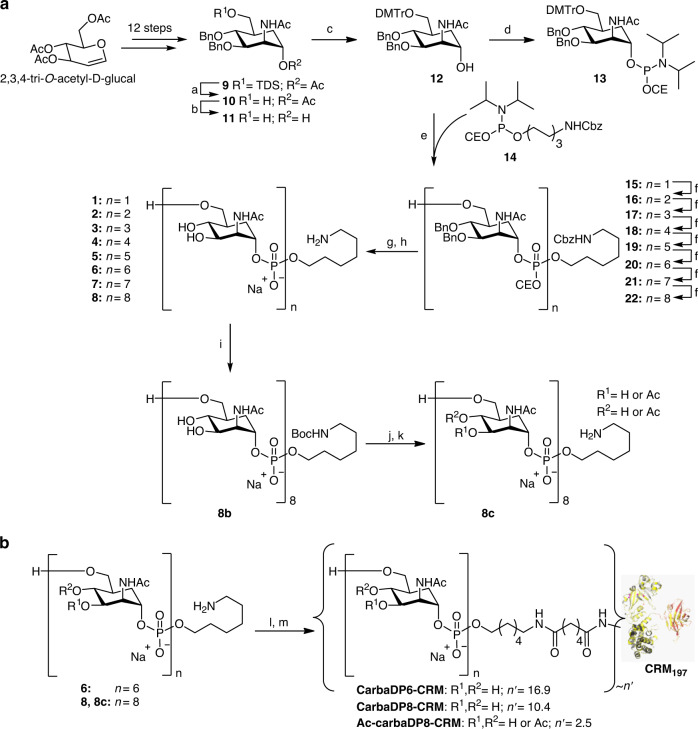
A: oligomer assembly. **a** TBAF, THF, 0 °C → rt, 92%. **b** NaOMe, MeOH, rt, 85%. **c** DMTrCl, Et_3_N, DCM, rt, 91%. **d** 2-cyanoethyl N,N-diisopropyl-chlorophosphoramidite, N,N-diisopropylethylamine, DCM, rt, 13 (94%). **e** I. 14, DCI, MeCN, II. CSO, MeCN, III. TCA, DCM, H_2_O, 94%. **f** I. 13, DCI, MeCN, II. CSO, MeCN, III. TCA, DCM, H_2_O, 16 (82%), 17 (95%), 18 (90%), 19 (92%), 20 (88%), 21 (86%), 22 (87%). **g** NH_4_OH, H_2_O, dioxane. **h** H_2_, Pd black, H_2_O, AcOH, 1 (99%), 2 (76%), 3 (69%), 4 (39%), 5 (88%), 6 (83%), 7 (77%), 8 (44%). **i** (Boc)_2_O, NaHCO_3_, rt, 16 h. **j** Ac_2_O/imidazole, 40 °C, ~9d. **k** TFA, rt, 1 h 56% over 3 steps. B: Conjugation to carrier protein. **l** di-N-hydroxysuccinimidyl adipate, TEA, DMSO. (**m**) CRM_197_, NaPi pH = 7.2.

Previously, we synthesized nonacetylated MenA carba-oligomers up to three repeating units using H-phosphonate methodology^32,36^. We envisaged that for longer fragments (oligomers **1**-**8**, Fig. 2a) the use of phosphoramidite building blocks would be more effective for the formation of the phosphodiester linkages^37,38^. In line with contemporary DNA chemistry and building on our recent experience in the generation of synthetic oligo(glycerolphosphate) and oligo(ribitolphosphate) teichoic acid antigens, we opted for the use of the dimethoxytrityl (DMTr) ether to temporarily mask the primary alcohol functions to be elongated^39–42^. Each elongation cycle is based on the iteration of a three-step sequence, comprising the coupling of the phosphoramidite with the growing chain alcohol, oxidation of the intermediate phosphite to the corresponding phosphotriester and unmasking of the primary hydroxyl on the (n + 1) oligomer. As illustrated in Scheme 1, the key elongation building block **13** is obtained from intermediate **12**, which in turn is derived in three steps from known carbasugar **9**^32^. The latter carba mannose building block can be prepared from the commercially available 3,4,6-tri-O-acetyl-D-glucal as we recently reported^32^. Thus, the primary silyl ether and acetyl ester were removed from compound **9** by the consecutive action of tetrabutylammonium fluoride and NaOMe, to give diol **11** in 78% yield over two steps. Next the DMTr group was regioselectively introduced providing alcohol **12** in 91% yield. This compound was converted into the elongation block phosphoramidite **13** in 94% yield by reaction with 2-cyanoethyl-N,N-diisopropyl-chlorophosphoramidite.

With the building block in hand, the target oligomers were assembled. The synthesis started with the installation of the aminohexanol spacer on alcohol **12** using known phosphoramidite **14**^38^. The building blocks were coupled in a two-step one pot reaction using dicyanoimidazole (DCI) for activation of the phosphoramidite. Oxidation of the in situ formed phosphite was carried out with (1 S)-(+)-(10-camphorsulfonyl)-oxaziridine (CSO). DCI (pK_a_ 5.2) was preferred over the conventionally used tetrazole (pK_a_ 4.9), because its lower acidity appeared more suited to preserve the acid labile DMTr group. CSO was used instead of iodine because of its higher solubility in nonaqueous solvents such as acetonitrile. The crude phophotriester was treated with trichloroacetic acid (TCA) to cleave the DMTr group and the product was purified by size-exclusion chromatography (Sephadex LH-20) giving the spacer-equipped monomer **15** in 94% yield. The subsequent couplings were all performed following the procedure described above. For elongation of the longer oligomers (*n* > 1), a larger amount of the phosphoramidite **13** was used and the coupling reaction time was increased to ensure complete conversion of the alcohol. The yield for each elongation cycle was good to excellent, ranging between 82 and 95%. Octamer **22** was obtained in 40% overall yield starting from **12**. Fragments **16**-**22** were deprotected using a two-steps sequence. First the cyanoethyl groups (OCE) were removed using an aqueous ammonia solution (33%). Next, all remaining protecting groups (the benzyl ethers and carboxybenzyl carbamate) on the so-formed phosphodiesters were cleaved off by hydrogenolysis over palladium black, to give the target oligomers **1**–**8**.

### Stability study of carbaMenA DP8

**Fig. 3:**
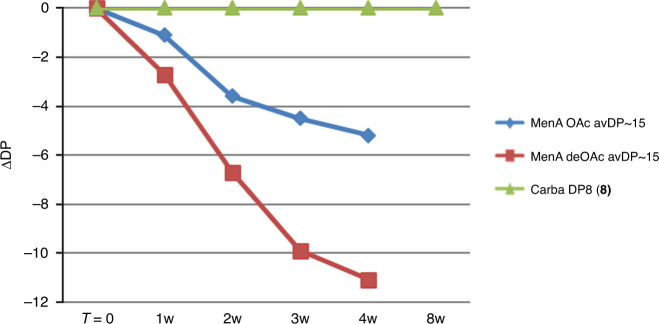
Stability of carba DP8. The sugar analog was compared to acetylated and de-O-acetylated MenA CPS avDP–15 at 37 °C in 5 mM NaOAc pH 7; ΔDP was measured as a function of the time based on ^31^P NMR profile, as shown in Supplementary Fig. 1.

The reduction of the polysaccharide size over the time could be detrimental for vaccine activity. To confirm the predicted higher stability of carbaMenA analogs we undertook an accelerated stability study, monitoring the change of chain length in buffered 5 mM sodium acetate pH 7 at 37 °C.^19^ The results of the accelerated stability studies are generally considered predictive of the behavior at the recommended storage temperature (usually 2–8 °C)^22^. Compound **8** was compared to the natural polysaccharide portion with an average DP (avDP) of ~15, obtained according to reported procedures^43^, and the corresponding de-O-acetylated form (deOAc), prepared by treatment of the same fragment with 0.5 M NaOH. ^1^H and ^31^P NMR spectra were recorded at day 0, 7, 14, 21, and 28 (+56 for the carba structure), and results are reported in Fig. 3 as a function of ΔDP (DP starting material-DP at the time point). The avDP of the two fragments deriving from polysaccharide was obtained from the ^31^P NMR signals and expressed as [(P_Int_/P_Ter_) + 1], where P_Int_ is molar concentration of the internal phosphate groups (phosphodiester groups) and P_Ter_ the molar concentration of terminal phosphate groups (phosphomonoester groups) as previously reported^19^. In the ^31^P NMR spectrum the ^31^P monoester signal typically appears at *δ* 1–5 ppm^44^. The carba DP8 exhibited neither change in the ^1^H NMR-spectrum nor detectable appearance of monoester peaks at the ^31^P NMR at day 56 (Supplementary Figs. 1 and 2). Treatment of this sample with 0.5 M NaOH, at 37 °C for 3 days was performed for complete hydrolysis of the structure into monomers. This generated two monoesters peaks at 3.80 and 5.10 ppm, respectively, which were assigned to the phosphorylated H-1 positions of the monomers and linker deriving from hydrolysis. In contrast, the intensity of the monoesters signals relative to the phosphorylated H-1 of the OAc and de-*O*-acetylated (deOAc) MenA fragments at 3.73 and 4.20 ppm, respectively, clearly increased over the time. A dramatic size reduction was found particularly for the nonacetylated avDP~15 fragment, which dropped down to avDP7 already at day 28. This clearly proved the effect of replacement of the ring oxygen with a methylene in stabilizing the molecule. A less severe reduction in size was observed for the acetylated natural MenA avDP~15 fragment, whose avDP decreased from 18 to 12.8 at day 28, as previously reported, highlighting a possible role of the electronwithdrawing acetyl substituent in stabilizing the natural polymer^19^.

### In vitro selection of oligomer length

**Fig. 4:**
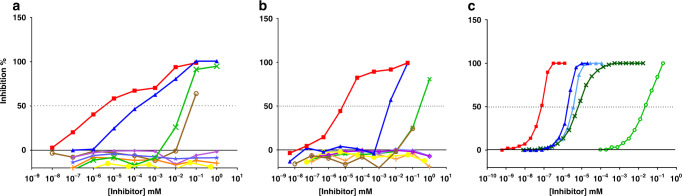
Inhibition of the binding of anti-MenA antibodies to CPS. Competitive ELISA with anti-MenA mAb (**a**) and anti-MenA polyclonal serum (**b**) using different length nonacetylated carbaMenA oligomers as inhibitors and CPS as coating. **c** Competitive SPR of binding between anti-MenA mAb and immobilized biotylinated CPS. Inhibitors were reported as described: red curve for MenA CPS; blue curve MenA avDP–15; light blue curve MenA DP8; brown curve MenA deOAc CPS; green curve carba DP8; dark green curve carbaDP8 OAc; purple curve carba DP4; light purple carba DP6; orange curve carba DP7; yellow curve negative control. MenA CPS and deOAc CPS were the positive controls and the β-glucan Laminarin was the negative control in **a** and **b**. MenA CPS and fragments thereof were used as positive controls and anti-MenC mAb was flown on the chip as negative control in **c**. Data from a single experiment representative of two experiments is shown.

An important prerequisite for the immunogenicity of the carba analogs is their ability to mimic the natural MenA CPS. To investigate this property in vitro, binding of oligomers with the different length to a bactericidal anti-MenA mAb (JW-A1)^45–47^ in comparison to CPS and a medium length oligomer with avDP ~15 was tested (Fig. 4a). Remarkably, while DPs from 4 to 7 did not recognize the mAb, binding was observed with DP8, although with 4 orders of magnitude higher IC_50_ compared to the native CPS. De-O-acetylation of MenA CPS led to diminished recognition, signifying the specificity of the mAb for acetylated epitopes. A similar behavior was observed when the inhibitors were assayed with an anti-MenA polyclonal serum generated by immunization of mice with a MenA-CRM_197_ conjugate (Fig. 4b), when the carba DP8 performed as inhibitor comparably to the deOAc CPS. This corroborated the propensity of carba DP8 to anti-MenA antibody binding. The different fragments were then tested against an anti-deOAc MenA serum (Supplementary Fig. 3). In this case the deOAc CPS (IC_50_ = 1.86 × 10^−6^) inhibited similarly to the Ac counterpart (Supplementary Table 1), indicating the specificity of the serum for the CPS backbone independently of the acetylation pattern. Most importantly, recognition was observed for all the carba DP4-8 analogs, demonstrating that they all resembled deacetylated MenA CPS portions. Of note, the binding affinity of carba DP8 (IC_50_ = 1.59 × 10^−2^ mM) was one order of magnitude higher than DP7 (IC_50_ = 1.84 × 10^−1^ mM). Based on these results DP8 and DP6 were selected for conjugation to carrier protein to compare the capacity of the two oligomers to elicit antibodies in mice, the first clearly binding to anti-MenA antibodies and the latter showing no recognition.

### Conjugation of carbaMenA DP6 and DP8 to CRM_197_

Compounds **6** and **8** were coupled to CRM_197_ using a conjugation procedure previously reported, taking advantage of the di-N-hydroxysuccinimidyl adipate linker (Fig. 2b)^48,49^. Conjugates produced by this method are known not to elicit anti-linker antibodies^50^. After treatment of the amines of **6** and **8** with the spacer in DMSO containing triethylamine, the obtained activated oligomer was purified by co-precipitation with acetone and used for conjugation. Overnight incubation with CRM_197_ at a 100:1 glycan/protein molar ratio (corresponding to ~40-50 active ester: protein, as determined by NHS spectrophotometric quantification) in buffered pH 7.2 solution, gave the desired neo-glycoconjugate, as assessed by SDS page gel electrophoresis (Supplementary Fig. 4), which was purified by dialysis against a 30 kDa MW cutoff membrane^51^. A saccharide/protein molar ratio of 16.9 and 10.4 was determined by MALDI TOF MS for the two conjugates from carba DP6 and DP8, respectively.

### Immunogenicity of the carbaMenA CRM_197_ conjugates

**Fig. 5:**
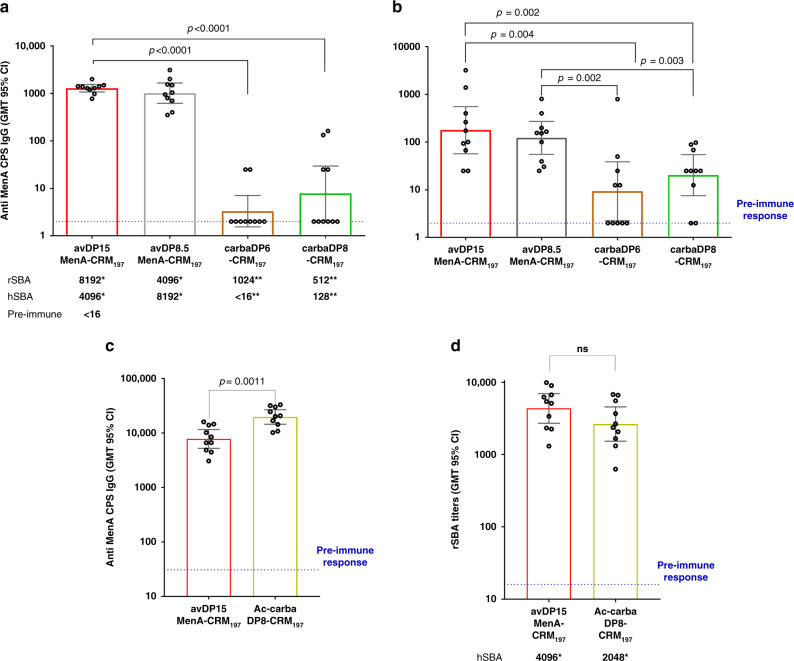
Immune response elicited by the neo-glycoconjugates. Antibody titers are reported in the **a**–**c** panel as Geometric Mean (horizontal bar) with the 95% of CI (vertical bar). rSBA titers are reported in the **d** panel as Geometric Mean (horizontal bar) with the 95% of CI (vertical bar). Two-tailed Mann-Whitney test was used to compare ranks; *n* = 10. Pre-immune was the negative control in both type of analysis. **a** Anti-MenA IgG titers estimated in individual murine sera after the second boost against the natural MenA CPS. *p* < 0.0001 between avDP~15 MenA and carbaDP6/DP8 conjugates. **b** Anti-deOAc MenA IgG titers determined against the de-O-acetylated MenA CPS conjugated to HSA. *p* = 0.002 between avDP~15 and carbaDP8 conjugates, and between avDP8.5 and carbaDP6 conjugates; *p* = 0.003 between avDP8.5 and carbaDP8 conjugates; and *p* = 0.004 between avDP~15 and carbaDP6 conjugates comparison. **c** Anti-MenA IgG titers estimated in individual sera after the second boost using MenA CPS for coating. *p* = 0.0011 comparing avDP15 and Ac-carbaDP8 conjugates. **d** Human and rabbit serum bactericidal titers measured after the third injection on pooled and individual mice sera, respectively. Not significant differences were found comparing the ranks. Immunizations were conducted in duplicates and data from a representative experiment are here shown. *Human and rabbit SBA titers measured after the third injection on pooled sera; **Human and rabbit SBA titers measured after the third injection on pooled sera from responder mice.

To test the immunogenicity of the conjugated carbaMenA DP6 and DP8, groups of eight BALB/c female mice were immunized with the neoglycoconjugates. Conjugates prepared as previously described from sized MenA polysaccharide with avDP8.5 and ~15 were used as controls^33^. Mice received three subcutaneously (s.c.) doses (2 µg on saccharide base), two weeks apart. The neo-glycoconjugate induced an immune response at week 3, as observed by assaying the sera elicited by the conjugate against the same product coated on ELISA plates (Supplementary Fig. 5). At the serum dilutions tested no anti-CRM_197_ antibodies were detectable. Each of the conjugates gave antibodies recognizing the conjugated hapten and the specificity of this recognition was confirmed by competitive ELISA (Supplementary Fig. 5). The binding of the anti-carbaMenA DP8 serum was inhibited by the unconjugated octamer to a greater extent than its conjugated form, due to the multivalent exposition of the hapten. Furthermore, this binding was inhibited for ~25% by the deOAc CPS and almost equally by the naturally acetylated counterpart, anticipating the potential of the raised antibodies in recognizing the capsule structure. To determine the capacity of the elicited antibodies to cross-react with the backbone CPS structure deprived of the acetyl substituents, the sera were assayed against the nonacetylated CPS conjugated to Human Serum Albumin (HSA). The carbaMenA conjugates clearly showed an anti-deOAc CPS immune response, but IgG titers were inferior to those elicited by the conjugated avDP8.5 and ~15 used as controls (*p* ≤ 0.003, Fig. 5b). The response of the two carba analogs conjugates was not statistical different, however the number of responder mice to the DP8 conjugate was higher than for the DP6 conjugate.

When the ELISA was conducted using the acetylated CPS as coating reagent, the difference between the anti-native MenA IgG titers elicited by the carbaMenA DP6 and DP8 neoglycoconjugates and the controls was even more evident (*p* < 0.0001, Fig. 5a). The functionality of the elicited antibodies was next assessed on pooled sera by measuring the complement mediate lysis of bacteria expressing the acetylated CPS, as reported in literature^33,52^. This assay is considered predictive of protection in humans for meningococcal vaccines^53^. It revealed poor bactericidal activity for the pooled serum from responder mice raised using the carbaMenA DP6 and DP8 conjugates (1024 vs 512, respectively). When the Serum Bactericidal Activity (SBA) was measured using human complement, the carba DP8-CRM_197_ showed a titer of 128, significantly lower that the SBA of serum generated by the benchmark vaccine, based on the natural MenA CPS avDP~15. These results are in line with the observation reported in literature that antibodies elicited by a conjugate of the deOAc MenA CPS are scarcely functional^35^.

Taken together, this data revealed that the carbaMenA DP8 is an effective, stable mimic of the MenA CPS, capable of binding anti-MenA CPS antibodies. The carbaMenA DP8 conjugate induced antibodies able to activate human complement deposition, while the carbaMenA DP6 did not, highlighting the DP8 molecule as lead antigen. The carbaMenA DP8 neoglycoconjugate vaccine, however, elicited only low levels of bactericidal anti-MenA antibodies. Considering that the MenA CPS is variably *O*-acetylated at position 3 and 4, we therefore sought to further increase the resemblance to the natural polysaccharide and increase the generation of protective antibodies by randomly *O*-acetylating the carbaMenA DP8 lead molecule.

### Optimization of the glycomimetic vaccine candidate

To introduce acetyl esters on carbaMenA DP8, we first installed a temporary Boc protecting group on the amine group of the linker (Fig. 2a). The resulting compound was next carefully acetylated by treatment with Ac_2_O/imidazole to reach an acetylation level of 75%, similarly to the natural CPS. Boc-deprotection then provided the conjugation-ready Ac-carbaMenA DP8 **8c**. NMR analysis revealed the acetyls to reside on either the C-3 or C-4 positions, with also concomitant acetylation at C-3/4 up to an extent of 44% (see Supplementary Fig. 6 and other Supplemental Information for full characterization). To test this compound as a potential antigen, we first evaluated binding with the anti-MenA CPS mAb, in a competitive Surface Plasmon Resonance (SPR) experiment (Fig. 4c). This SPR was optimized to increase the sensitivity of the assay compared to the previously done standard ELISA. As shown in Fig. 5c, Ac-carbaMenA DP8 **8c** exhibited a 4-log higher capacity as inhibitor compared to its nonacetylated counterpart **8**, and binding to the mAb was almost comparable to the natural avDP8 and avDP~15 oligomers. Based on this promising result, **8c** was conjugated to CRM_197_ through the two-step procedure used for the nonacetylated oligomers, involving reaction with the di-N-hydroxysuccinimidyl adipate spacer and incubation with CRM_197,_ in 13:1 active ester: protein molar ratio, based on NHS spectrophotometric quantification (Fig. 2b). The purified neo-glycoconjugate was used in an immunization study with ten BALB/c female mice, using the avDP~15 CRM_197_ conjugate as comparator. After three s.c. injections (2 µg on saccharide base), the sera were collected and analyzed for the content of bactericidal IgGs (Fig. 5c).

Remarkably, the Ac-carbaMenA DP8 conjugate induced higher levels of anti-MenA CPS antibodies compared to the control. SBA titers analyzed in individual mice also showed that the synthetic antigen was able to induce rabbit complement mediated bactericidal killing of MenA bacteria statistically noninferior to the vaccine benchmark (Fig. 5d). Analysis in pooled sera confirmed that the human complement mediated bactericidal activity was also comparable between Ac-carbaMenA DP8 and the natural avDP~15, revealing Ac-carbaMenA DP8 as a true and potent mimic of the MenA CPS, that can be used in the generation of a stabilized neoglycoconjugate vaccine.

## Discussion

The availability of stable MenA polysaccharide analogs is very attractive for the development of a stable MenA glycoconjugate vaccine. To overcome the intrinsic lability of the anomeric phosphodiester linkages that connect the repeating units in the natural CPS, we have replaced the ring-oxygen of the constituting N-Acetyl mannosamines for a methylene entity, thereby generating carbaMenA oligomers. Using tailor made phosphoramidite building blocks we have prepared oligomers ranging from 1 to 8 monomers in length, each equipped with a chemoselective ligation handle. The substitution of the ring oxygen with a methylene in the carba-analogs resulted in a much higher stability, particularly when compared to the nonacetylated natural CPS fragment. CarbaMenA oligomers in the rage of DP4-8 inhibited the recognition of anti-de-OAc MenA antibodies directed to the deacetylated backbone of the capsule in a length-dependent manner. However, only carbaMenA DP8 competed for binding of an anti-MenA functional mAb, indicating that 8 repeating units are required to provide an effective synthetic antigen. While in silico analysis of carbaMenA related monomers has shown similar conformations with the natural sugar^54^, studies with oligomers up to the dodecamer have indicated that these longer structures may adopt a different overall conformation than their natural counterparts^55^. The results presented here show that the carbaMenA can adequately mimic the natural polysaccharide.

A CRM_197_ conjugate of the nonacetylated carba-MenA DP6 and DP8 were generated and used to induce murine antibodies that not only recognized the haptens which they were induced to, but were also capable of cross-reacting with the nonacetylated CPS backbone. However, only moderate levels of antiacetylated CPS IgGs, able to induce in vitro rabbit complement mediated bactericidal activity, were raised. Remarkably, serum from the carbaMenA DP8 conjugate was able to active human complement deposition. To further improve on its activity, the carbaMenA DP8 candidate was optimized by randomly acetylating the structure at a level comparable to the natural CPS. The structure exhibited a 4-log higher capacity to inhibit the binding of a protective mAb with the MenA CPS compared to its nonacetylated form. Most importantly, after conjugation to CRM_197_ the randomly acetylated carbaMenA DP8 elicited high level of anti-MenA CPS antibodies with bactericidal activity comparable to the MenA vaccine benchmark.

Synthetic oligosaccharides are widely explored as surrogates for natural polysaccharides in the generation of anti-bacterial vaccines. Generally, all these synthetic structures try to accurately duplicate their natural counterparts. We now show that synthetic chemistry can deliver mimetics with improved, tailor-made features, opening up new avenues in the design of carbohydrate-based vaccines^23^. Considering that the random O-acetylation at C3 and C4 proved essential to fully resemble the natural MenA polysaccharide, the role of these positions in the immunogenicity will deserve further studies^18^. Future efforts will be directed at the site-selective acetylation of the lead antigen carbaMenA DP8. The chemistry developed here will allow for the assembly of (acetyl functionalized) carbaMenA oligomers through automated syntheses protocols to generate a larger library of potential synthetic antigens to define the precise pattern responsible for the herein reported strong immune response.

## Methods

### General procedure A

Phosphoramidite coupling, oxidation, and detritylation on a typical scale (0.03–0.3 mmol): Starting alcohol was coevaporated 3 times with ACN, and was added freshly activated MS3Å and DCI (0.25 M solution in ACN, 1.5 eq). The solution was stirred for 15 min. To the mixture was added phosphoramidite reagent (0.1–0.16 M solution in ACN, 1.3–3 eq) and stirred until the total conversion of the starting material (≈2 h). Subsequently CSO (0.5 M solution in ACN, 2eq) was added to the reaction mixture and stirred for 15 min. The mixture was diluted with EtOAc and washed with a 1:1 solution of brine/NaHCO_3_. The water layer was extracted 2 times with EtOAc. The organic layer was dried over Na_2_SO_4_ and concentrated in vacuo. The crude was coevaporated 3 times with ACN and dissolved in DCM (5–10 mL). To the solution was added TCA (0.18 M solution in DCM) and stirred for 1 h. To the reaction mixture was added H_2_O and stirred for 15 min. The reaction was washed with a 1:1 solution of brine/NaHCO_3_. The water layer was extracted with DCM 3 times. The organic layer was dried over Na_2_SO_4_ and concentrated in vacuo. The crude was purified by flash chromatography (DCM/Acetone) or by size exclusion chromatography (Sephadex LH-20, MeOH/DCM 1:1).

### General procedure B

Deprotection on a typical scale (5–40 µmol): Starting alcohol was dissolved in NH_3_ (aqueous solution 30–33%, 1 mL per 10 µmol) and dioxane (until it completely dissolved). The reaction mixture was stirred for 2 h. The mixture was concentrated in vacuo. ^1^H NMR and ^31^P NMR analysis showed a total conversion to the semi-protected intermediate. The crude was dissolved in MilliQ H_2_O and eluted through a column containing Dowex Na^+^ cation-exchange resin (type: 50WX4-200, stored on a 0.5 M NaOH in H_2_O, flushed with MilliQ H_2_O and MeOH before use). The crude was dissolved in MilliQ H_2_O (2 mL per 10 µmol). To the reaction mixture was added 4–5 drops of glacial AcOH. The mixture was purged with Ar. To the solution was added a scup of Pd black. The reaction mixture was purged with H_2_ for a few seconds and stirred under H_2_ atmosphere for 3 days. To the mixture was added celite. The solution was filtrated and concentrated in vacuo. The crude was purified by size-exclusion chromatography (Toyopearl HW-40). The pure compound was dissolved in MilliQ H_2_O, eluted through a column containing Dowex Na^+^ cation-exchange resin (type: 50WX4-200, stored on a 0.5 M NaOH in H_2_O, flushed with MilliQ H_2_O and MeOH before use) and lyophilized.

### Preparation of neoglycoconjugates

In a typical experiment, 5–10 mg of the amino-oligosaccharides were vacuum dried, solubilized in 1:9 H_2_O: DMSO solution to a final amino group concentration of 40 mmol mL^−1^, and reacted with a 12-fold molar excess of di-N-hydroxysuccinimidyl adipate linker (SIDEA), in presence of fivefold molar excess triethylamine as compared with amino groups. The reaction was kept under gentle stirring at room temperature for 3 h. The activated oligosaccharides were purified by precipitation with four volumes of ethyl acetate followed by ten washes of the pellet with 1 mL of the same solvent. Finally the pellet was dried under vacuum, and the content of introduced N-hydroxysuccinimide ester groups was determined^44^.

Conjugates were prepared in 50 mM NaH_2_PO_4_ pH 7 using an active ester: protein molar ratio of 40-50:1 for **6** and **8** and 13:1 for **8c**, at the final protein concentration of 40 mg mL^−1^. The reaction was kept overnight at room temperature with gentle stirring. The conjugates were purified by tangential flow filtration (Vivaspin) using a cutoff of 30 kDa and using PBS pH 7.2 as buffer. Conjugates were characterized by micro BCA for total protein content and by MALDI analysis for total saccharide content.

### Mice immunization and ELISA analysis

Experiments were undertaken in accordance with the regulations of the Directive 2010/63/EU and GSK ethical guidelines, under approval of the Italian Ministry of Health. All mice were housed under specific pathogen-free conditions at the GSK Vaccines Animal Resource Centre. Antigens formulations have been prepared under sterile conditions. Groups of 10 BALB/c mice were immunized on days 1, 14 and 28; bleedings were performed on day 0 (pre immune), day 27 (post 2) and day 42 (post 3). Vaccines were administered in saccharide dose and the dosage of 2 μg/mice per dose in terms of saccharide. Adjuvant AlPO_4_ was used at the dose of 0.12 mg of Al^3+^. The antibody response induced by the glycoconjugates has been measured by ELISA. The pre-immune serum was used as negative control in this analysis. Plates were coated with HSA-deOAc or MenA CPS by adding 100 μL/well of a 5 μg mL^−1^ polysaccharide solution in PBS buffer at pH 8.2 followed by incubation overnight at 4 °C^46^. HSA-deOAc MenA CPS, CRM_197_ conjugates and CRM_197_ were coated at the protein concentration of 2 μg mL^−1^ in pH 7.2 PBS buffer. Coating solutions were removed from the plates by washing tree times with PBS buffer with 0.05% of Tween 20 (Sigma) (TPBS). A blocking step was then performed by adding 100 μL/well of BSA solution at 3% in TPBS and incubating the plates 1 h at 37 °C. Blocking solution was removed from the plates by washing three times with TPBS. A 200 μL/well of pre-diluted serum (1:25 for pre immune negative control, 1:200/1:500 for a reference serum and from 1:25/1:200 for test sera) was added in the first well of each column of the plate, while on the other wells 100 μL of TPBS was dispensed. Eight two-fold serial dilutions along each column were then performed by transferring from well to well 100 μL of sera solutions. After primary antibody dilution, plates were incubated for 2 h at 37 °C. Three washes with TPBS, 100 μL/well TPBS solutions of secondary antibody alkaline phosphates conjugates (anti mouse IgG 1:10000, Sigma-Aldrich) were then added, and the plates incubated 1 h at 37 °C. After three more washes with TPBS, 100 μL/well of a 1 mg mL^−1^ of p-NPP (Sigma) in a 0.5 M di-ethanolamine buffer pH 9.6 was added. Finally, plates were incubated for 30 min at room temperature and read at 405 nm using the plate reader Spectramax 190. Sera titers were expressed as the reciprocal of sera dilution corresponding to a cut-off OD = 1.

Each immunization group has been represented as the geometrical mean (GMT) with 95% CI of the single mouse titers. The statistical and graphical analysis has been done by GraphPad Prism 7 software.

### In vitro bactericidal assay

Functional antibodies induced by vaccine immunization were analyzed by measuring the complement-mediated lysis of *N. meningitidis* with an in vitro bactericidal assay^56^. A commercial lot of baby rabbit complement (Peel Freeze Biological, cod. 31061) was used as source of active complement for rSBA. The use of human complement samples deriving from the study protocol V72_92 was authorized by GSK HBS Reuse Check Team and performed upon written informed consent obtained from participants before the study-specific procedures.

*N. meningitidis* strain was grown overnight on chocolate agar plates at 37 °C in 5% CO_2_. Colonies were inoculated in Mueller-Hinton broth, containing 0.25% glucose to reach an OD600 of 0.05-0.08 and incubated at 37 °C with shaking. When bacterial suspensions reached OD600 of 0.25–0.27, bacteria were diluted in the assay buffer (DPBS with 1 % BSA and 0.1% glucose) at the working dilution (ca. 10^4^ CFU mL^−1^). The total volume in each well was 50 μL with 25 μL of serial two-fold dilutions of the test serum, 12.5 μL of bacteria at the working dilution and 12.5 μL of complement source. The tested sera were pooled and heat-inactivated for 30 minutes at 56 °C. Negative controls included bacteria incubated, separately, with the complement serum without the test serum and with test sera and the heat-inactivated complement. Immediately after the addition of the baby rabbit complement, negative controls were plated on Mueller-Hinton agar plates, using the tilt method (time 0). The microtiter plate was incubated for 1 h at 37 °C, then each sample was spotted in duplicate on Mueller-Hinton agar plates while the controls were plated using the tilt method (time 1). Agar plates were incubated overnight at 37 °C and the colonies corresponding to time 0 and time 1 (surviving bacteria) were counted. The serum bactericidal titers was defined as the serum dilution resulting in 50% decrease in colony forming units (CFU) per mL, after 60 min incubation of bacteria in the reaction mixture, compared to control CFU per mL at time 0. Typically, bacteria incubated without the test either pooled or individual murine serum in the presence of complement (negative control) showed a 150–200% increase in CFU mL^−1^, during the 60 min incubation time. The reference strain for meningococcal serotype A was F8238^57^.

### Reporting summary

Further information on research design is available in the Nature Research Reporting Summary linked to this article.

## Supplementary information

Supplementary Information

Peer Review File

Reporting Summary

## Data Availability

The authors declare that the data supporting the findings of this study are available within the paper and its Supplementary Information files. Source data are provided with this paper.

## Source data

Source Data
