# Publisher Correction: A stabilized glycomimetic conjugate vaccine inducing protective antibodies against *Neisseria meningitidis* serogroup A

**DOI:** 10.1038/s41467-020-20120-4

**Published:** 2020-11-25

**Authors:** Jacopo Enotarpi, Marta Tontini, Cristiana Balocchi, Daan van der Es, Ludovic Auberger, Evita Balducci, Filippo Carboni, Daniela Proietti, Daniele Casini, Dmitri V. Filippov, Hermen S. Overkleeft, Gijsbert A. van der Marel, Cinzia Colombo, Maria Rosaria Romano, Francesco Berti, Paolo Costantino, Jeroen D. C. Codeé, Luigi Lay, Roberto Adamo

**Affiliations:** 1 Department of Chemistry and CRC Polymeric Materials (LaMPo), University of Milan, Milan, Italy; 2 Department of Bioorganic Synthesis, Leiden University, 2333 Leiden, The Netherlands; 3 GSK, Via Fiorentina 1, 53100 Siena, Italy

**Keywords:** Glycoconjugates, Lead optimization, Conjugate vaccines

Correction to: *Nature Communications* 10.1038/s41467-020-18279-x, published online 7 September 2020.

The original version of this Article contained errors in Fig. 1. The label ‘a’ was omitted for the left panel and the label ‘b’ was omitted for the right panel. In Fig. 1b, the right parenthesis was incorrectly positioned indicating a 4-carbon linker, rather than the correct 6-carbon linker. These errors have been corrected in both the PDF and HTML versions of the Article.

