# Supplementary material for: A stabilized glycomimetic conjugate vaccine inducing protective antibodies against Neisseria meningitidis serogroup A
Source: Nat Commun. 2020 Sep 7;11:4434. doi: 10.1038/s41467-020-18279-x (PMC7477203; doi:10.1038/s41467-020-18279-x)
Supplement: Supplementary file 1 — Supplementary Information [file 41467_2020_18279_MOESM1_ESM.pdf]

Supplementary Information

A stabilized glycomimetic conjugate vaccine inducing protective antibodies against *Neisseria meningitidis* serogroup A

Jacopo Enotarpi[1,3†], Marta Tontini[2†], Cristiana Balocchi[2], Daan van der Es[3], Ludovic Auberger[1], Evita Balducci[2], Filippo Carboni[2], Daniela Proietti[2], Daniele Casini[2], Dmitri V. Filippov[3], Hermen S. Overkleeft[3], Gijsbert A. van der Marel[3], Cinzia Colombo[1], Maria Rosaria Romano[2], Francesco Berti[2], Paolo Costantino[4], Jeroen D. C. Codeé[3]*, Luigi Lay[1]*, Roberto Adamo[2]*

[1]*Department of Chemistry and CRC Polymeric Materials (LaMPo) , University of Milan, Milan, Italy.*
[2] *GSK, Via Fiorentina 1, 53100 Siena, Italy*
[3]*Department of Bioorganic Synthesis, Leiden University, 2333 Leiden, The Netherlands.*
[4] *Former employee of GSK Group of Companies, now retired.*
† *These authors contribute equally to the work*

*email: roberto.x.adamo@gsk.com; luigi.lay@unimi.it; jcodee@chem.leidenuniv.nl.

Contents

Supplementary Methods 3
Synthesis of CarbaMenA oligomers 3
General procedures and materials 3
Stability of carba DP8 compared to Ac and DeOAc MenA avDP18 9
In vitro evaluation of carbaMenA vs de-O-acetylated MenA CPS 10
Competitive ELISA analyses 10
Characterization of the CarbaMenA neoglycoconjugates 12
MALDI-TOF mass analysis 12
Sodium Dodecyl Sulfate- Polyacrilammide gel electrophoresis (SDS-Page) 12
Immunogenicity of Carba DP8 against itself 13
Synthesis of randomly acetylated CarbaMenA DP8 14
SPR mAb competitive binding 15
Conjugation of Ac-CarbaMenA to CRM_{197} 15
Quantification of conjugated random Ac-CarbaMenA 16
Supplementary Figures 17
NMR spectra of synthesized compounds 17

Supplementary Methods

Synthesis of CarbaMenA oligomers

General procedures and materials

All chemicals (Acros, Biosolve, Sigma-Aldrich and TCI) were used as received and all reactions were effectuated under an argon atmosphere, at ambient temperature (22°C), unless stated otherwise. For the TLC analysis were used aluminium sheets (Merck, TLC silica gel 60 F_{254}), sprayed with a solution of H_2SO_4 (20%) in EtOH or with a solution of $(NH_4)_6Mo_7O_{24}$•$4H_2O$ (25 g L^{-1}) and $(NH_4)_4Ce(SO_4)_4$•$2H_2O$ (10g L^{-1}) in 10% aqueous H_2SO_4 or with a solution of $KMnO_4$ (2%) and K_2CO_3 (1%) in H_2O and then heated at ≈ 140°C. For the column chromatography was used 40-63 µm 60Å silica gel (SD Screening Devices). NMR spectra (^1H, ^{13}C and ^{31}P) were recorded with a Bruker AV-400liq or a Bruker DMX-400solid or a Bruker AV-500 or a Bruker AV-600. High resolution mass spectra were recorded by direct injection on a mass spectrometer (Thermo Finnigan LTQ Orbitrap) equipped with an electrospray ion source in positive mode (source voltage 3.5 kV, sheath gas flow 10, capillary temperature 250°C) with resolution R= 60000 at m/z 400 (mass range m/z= 150-2000) and dioctylphthalate (m/z= 391.28428) as a lock mass.

Acetamido-3,4-di-*O*-benzyl-2-deoxy-6-*O*-thexyldimethylsilyl-5a-carba-α-D-mannopyranose (10). Silyl ether **9** (1.6 g, 2.7 mmol) was dissolved in dry THF (20 mL). The mixture was cooled down to 0°C. A 0.1 M solution in THF of TBAF (4.1 mL, 4.1 mmol) was slowly added. The reaction was heated up to room temperature and stirred for 3h. To the reaction was added AcOH (0.31 mL). The solution was extracted 3 times with DCM and washed once with brine. The organic layer was dried over Na_2SO_4 and concentrated in *vacuo.* The crude was purified by flash chromatography (EtOAc/Hexane) leading to product **10** (1.1 g, 2.52 mmol) in 92% yield. The spectroscopic data were in agreement with the reported data.[25]

2-Acetamido-3,4-di-*O*-benzyl-2-deoxy-5a-carba-α-D-mannopyranose (11). Alcohol **10** (1.12 g, 2.5 mmol) was dissolved in MeOH (32 mL). To the mixture was added NaOMe (0.03 g, 0.5 mmol). The reaction was stirred for 3h at room temperature. Amberlite H^+ resin was added until neutral pH was reached. The suspension was filtrated and concentrated in *vacuo*. ^1H NMR (400 MHz, $CDCl_3$) δ= 1.70 – 1.85 (m, 2H, H-5a), 1.90 (s, 3H, *Ac*NH), 2.19 – 2.23 (m, 1H, H-5), 3.60 – 3.79 (m, 3H, H-6, H-1), 3.83 – 3.90 (m, 1H, H-2), 3.91 – 3.99 (m, 1H, H-4), 4.14 – 4.23 (m, 1H, H-3), 4.33 – 4.41 (m, 1H, CH*H* Bn), 4.54 – 4.72 (m, 3H, CH_2 Bn,, CH*H* Bn), 5.79 (m, 1H, N*H*Ac), 7.22 – 7.42 (m, 10H, H_{arom}). ^{13}C NMR (100 MHz, $CDCl_3$) δ= 23.5 (CH_3 *Ac*NH), 30.6 (CH_2 C-5a), 39.5 (CH C-5), 53.5 (CH C-3), 64.1 (CH_2 C-6), 67.9 (CH C-4), 72.4 (CH_2 Bn), 73.8 (CH_2 Bn), 75.5 (CH C-1), 79.0 (CH C-4), 127.3 – 128.9 (CH_{arom}), 171.8 (C=O *Ac*NH). HRMS: $[C_{23}H_{29}NO_5 + H]^+$ calculated 400.21251, found 400.21179.

2-Acetamido-3,4-di-*O*-benzyl-*2*-deoxy-6-*O*-(bis(4-methoxyphenyl) (phenyl))-5-carba-α-D-mannopyranose (12). Diol **11** (0.9 g, 2.25 mmol) was dissolved in dry DCM (30 mL). To the mixture was added Et_3N (1.9 mL, 13.5 mmol). DMTrCl (1.16 g, 3.38 mmol) was added. The reaction was stirred for 2 hours. To the reaction was added H_2O and was washed once with brine. The organic layer was dried over Na_2SO_4 and concentrated in *vacuo*. The crude was purified by flash chromatography (EtOAc/Hexane) leading to product **12** (1.6 g, 2.04 mmol) in 91% yield. ^1H NMR (400 MHz, CD_3CN) δ= 1.70 – 1.85 (m, 1H, 5a'-H), 1.91 (s, 3H, *Ac*NH), 2.00 – 2.21 (m, 2H, 5a-H, 5-H), 3.01 – 3.19 (m, 1H, 6'-H), 3.27 – 3.37 (m, 1H, 6-H), 3.51 – 3.67 (m, 1H, H-4), 3.73 (s, 7H, H-3, 2x OMe), 4.06 – 4.20 (m, 1H, H-1), 4.22 – 4.32 (m, 1H, CH*H* Bn), 4.40 – 4.62 (m, 3H, CH_2 Bn, H-2), 4.65 – 4.73 (m, 1H, CH*H* Bn), 6.35 – 6.44 (m, 1H, N*H*Ac), 6.78 – 7.47 (m, 23H, H_{arom}). ^{13}C NMR (100 MHz,CD_3CN) δ= 23.2 (CH_3 *Ac*NH), 31.6 (CH_2 C-5a), 38.6 (CH C-5), 53.3 (CH C-2), 55.8 (2x CH_3 OMe),

64.6 (CH_2 C-6), 67.6 (CH C-1), 72.1 (CH_2 Bn), 73.8 (CH_2 Bn), 77.2 (CH C-4), 79.8 (CH C-3), 86.5 (C_q DMTr), 113.9 (CH_{arom}), 127.3 – 130.7 (CH_{arom}), 137.2 – 159.4 (5x C_q DMTr), 171.1 (C=O *Ac*NH). HRMS: $[C_{44}H_{47}NO_7 + Na]^+$ calculated 724.32501, found 724.32483.

1-*O*-((*N*,*N*-Diisopropylamino)-*O*-2-cyanoethyl-phosphoramidite))-2-Acetamido-3,4-di-*O*-benzyl-2-deoxy-6-*O*-(bis(4-methoxyphenyl)(phenyl))-5a-carba-α-D-mannopyranose (13). Alcohol **12** (1.5 g, 2.14 mmol) was co-evaporated 3 times with ACN, and dissolved in dry DCM (22 mL). To the mixture were added freshly activated MS3Å and DIPEA (dried over KOH pellets, 0.6 mL, 3.2 mmol). To the mixture was added 2-cyanoethyl N,N-diisopropyl-chlorophosphoramidite (0.6 mL, 2.6 mmol). The reaction was stirred for 2 hours. To the solution was added H_2O, and was washed once with a 1:1 solution of brine/$NaHCO_3$. The organic layer was dried over Na_2SO_4 and concentrated in *vacuo.* The crude was purified by flash chromatography (DCM/Acetone/Et_3N) leading to product **13** (1.81 g, 2.0 mmol) in 94% yield (mixture of diastereoisomers). ^{1}H NMR (400 MHz, CD_3CN) δ= 1.04 – 1.24 (m, 12H, 4x isopropylamino), 1.70 – 1.85 (m, 1H, 5a'-H), 1.92 (s, 3H, *Ac*NH), 2.00 – 2.21 (m, 2H, 5a-H, 5-H), 2.55 – 2.75 (m, 2H, CH_2 cyanoethyl), 2.98 – 3.10 (m, 1H, 6'-H), 3.27 – 3.37 (m, 1H, 6-H), 3.47 – 3.70 (m, 3H, 2x CH isopropylamino, H-4), 3.70 – 3.88 (m, 9H, H-3, CH_2 cyanoethyl, 2x OMe), 4.06 – 4.20 (m, 1H, H-1), 4.22 – 4.32 (m, 1H, CH*H* Bn), 4.40 – 4.62 (m, 3H, CH_2 Bn, H-2), 4.65 – 4.73 (m, 1H, CH*H* Bn), 6.35 – 6.44 (m, 1H, N*H*Ac), 6.78 – 7.47 (m, 23H, H_{arom}). ^{13}C NMR (100 MHz,CD_3CN) δ= 20.7 (CH_2 cyanoethyl), 22.9 (CH_3 *Ac*NH), 24.5 – 24.7 (2x CH_3 isopropylamino), 30.6 (CH_2 C-5a), 38.5 (CH C-5), 43.7 (2x CH isopropylamino), 51.7 (CH C-2), 55.5 (2x CH_3 OMe), 59.1 (CH_2 cyanoethyl), 64.2 (CH_2 C-6), 70.5 (CH C-1), 71.5 (CH_2 Bn), 74.3 (CH_2 Bn), 77.8 (CH C-4), 79.5 (CH C-3), 86.2 (C_q DMTr), 113.6 (CH_{arom}), 127.3 – 130.7 (CH_{arom}), 136.8 – 159.2 (5x C_q DMTr), 170 (C=O *Ac*NH).^{31}P NMR (162 MHz, CD_3CN) δ= 146.9, 147.26.

1-*O*-((2-Acetamido-3,4-di-*O*-benzyl-2-deoxy-5a-carba-α-D-mannopyranosyl-1-*O*-phosphoryl)2-cyanoethyl)-6-hexyl-benzyl-carbamate (15). Alcohol **12** (0.21 g, 0.3 mmol), was coupled to phosphoramidite **14** (2.5 mL 0.16M in ACN, 0.45 mmol), oxidized, detritylated using the general procedure as described above. The crude was purified by flash chromatography (DCM/Acetone) leading to product **15** (0.216 g, 0.282 mmol) in 94% yield. ^{1}H NMR (400 MHz, CD_3CN) δ= 1.24 – 1.40 (m, 4H, 2x CH_2 hexylspacer), 1.40 – 1.51 (m, 2H, CH_2 hexylspacer), 1.58 – 1.70 (m, 2H, CH_2 hexylspacer), 1.80 – 1.92 (m, 4H, 5a'-H, *Ac*NH), 1.96 – 2.02 (m, 2H, 5a-H, 5-H), 2.72 – 2.82 (m, 2H, CH_2 cyanoethyl), 2.96 (bs, 1H, OH), 3.02 – 3.12 (m, 2H, CH_2 hexylspacer), 3.56 – 3.74 (m, 3H, H-6, H-4), 3.76 – 3.84 (m, 1H, H-3), 3.95 – 4.07 (m, 2H, CH_2 hexylspacer), 4.08 – 4.20 (m, 2H, CH_2 cyanoethyl), 4.44 – 4.63 (m, 5H, H-1, H-2, CH_2 Bn, CH*H* Bn), 4.72 – 4.80 (m, 1H, CH*H* Bn), 5.03 (s, 2H, CH_2 Bn spacer), 5.70 (bs, 1H, NH), 6.49 – 6.60 (m, 1H, N*H*Ac), 7.23 – 7.44 (m, 15H, H_{arom}). ^{13}C NMR (100 MHz,CD_3CN) δ= 19.9 (CH_2 cyanoethyl), 22.8 (CH_3 *Ac*NH), 25.4 (CH_2 hexylspacer), 26.4 (CH_2 hexylspacer), 30.0 (CH_2 C-5a), 30.4 (CH_2 hexylspacer), 30.5 (CH_2 hexylspacer), 40.0 (CH C-5), 41.0 (CH_2 hexylspacer), 51.1 (CH C-2), 62.9 (CH_2 C-6), 63.0 (CH_2 cyanoethyl), 66.3 (CH_2 Bn spacer), 68.8 (CH_2 hexylspacer), 72.2 (CH_2 Bn), 74.0 (CH_2 Bn), 75.1 (CH C-1), 76.7 (CH C-4), 79.3 (CH C-3), 128.1 – 129.1 (CH_{arom}), 138.9 – 139.7 (3x C_q Bn), 170.8 (C=O *Ac*NH). ^{31}P NMR (162 MHz, CD_3CN) δ= –2.40, –2.36. HRMS: $[C_{40}H_{52}N_3O_{10}P + H]^+$ calculated 766.34707, found 766.34707.

1-*O*-di-((2-Acetamido-3,4-di-*O*-benzyl-2-deoxy-5a-carba-α-D-mannopyranosyl-1-*O*-phosphoryl)2-cyanoethyl)-6-hexyl-benzyl-carbamate (16). Alcohol **15** (0.186 g, 0.24 mmol), was coupled to phosphoramidite **13** (2.3 mL 0.16 M in ACN, 0.37 mmol), oxidized, detritylated using the general procedure as described above. The crude was purified by size exclusion chromatography (Sephadex LH-20, DCM/MeOH 1:1) leading to product **16** (0.255 g, 0.199 mmol) in 82% yield. ^{1}H NMR (400 MHz, CD_3CN) δ= 1.25 – 1.40 (m, 4H, 2x CH_2 hexylspacer), 1.40 – 1.51 (m, 2H, CH_2 hexylspacer), 1.58 – 1.71 (m, 2H, CH_2 hexylspacer), 1.80 – 1.92 (m, 8H, 2x 5a'-H, 2x *Ac*NH), 1.96 – 2.02 (m, 4H, 2x 5a-H, 2x 5-H), 2.70 – 2.81 (m, 4H, 2x CH_2 cyanoethyl), 2.96 (bs, 1H,

OH), 3.01 – 3.12 (m, 2H, CH_2 hexylspacer), 3.56 – 3.87 (m, 6H, 2x H-6, 2x H-4), 3.94 – 4.28 (m, 8H, 2x H-3, CH_2 hexylspacer, 2x CH_2 cyanoethyl), 4.29 – 4.85 (m, 12H, 2x H-1, 2x H-2, 4x CH_2 Bn), 5.03 (s, 2H, CH_2 Bn spacer), 5.75 (bs, 1H, NH), 6.52 – 6.62 (m, 1H, N*H*Ac), 6.85 – 6.99 (m, 1H, N*H*Ac), 7.21 – 7.41 (m, 25H, H_{arom}). ^{13}C NMR (100 MHz,CD_3CN) δ= 19.9 – 20.0 (2x CH_2 cyanoethyl), 22.9 – 23.0 (2x CH_3 *Ac*NH), 25.5 (CH_2 hexylspacer), 26.5 (CH_2 hexylspacer), 29.1 – 29.2 (2x CH_2 C-5a), 30.1 (CH_2 hexylspacer), 30.5 (CH_2 hexylspacer), 38.1 – 40.0 (2x CH C-5), 41.1 (CH_2 hexylspacer), 50.9 – 51.4 (2x CH C-2), 62.5 – 62.6 (2x CH_2 C-6), 63.0 – 63.2 (2x CH_2 cyanoethyl), 66.3 (CH_2 Bn spacer), 68.9 (CH_2 hexylspacer), 72.1 – 72.3 (4x CH_2 Bn), 75.0 – 75.4 (2x CH C-1), 75.5 – 76.9 (2x CH C-4), 79.2 – 79.5 (2x CH C-3), 128.2 – 129.1 (CH_{arom}), 138.9 – 139.6 (5x C_q Bn), 170.8 (2x C=O *Ac*NH). ^{31}P NMR (162 MHz, CD_3CN) δ= −2.60, −2.58, −2.34, −2.32, −2.22, −2.17. HRMS: $[C_{66}H_{83}N_5O_{17}P_2 + H]^+$ calculated 1280.53320, found 1280.53320.

1-*O*-tri-((2-Acetamido-3,4-di-*O*-benzyl-2-deoxy-5a-carba-α-D-mannopyranosyl-1-*O*-phosphoryl)2-cyanoethyl)-6-hexyl-benzyl-carbamate (17). Alcohol **16** (0.215 g, 0.167 mmol), was coupled to phosphoramidite **13** (1.6 mL 0.16 M in ACN, 0.25 mmol), oxidized, detritylated using the general procedure as described above. The crude was purified by size exclusion chromatography (Sephadex LH-20, DCM/MeOH 1:1) leading to product **17** (0.285 g, 0.158 mmol) in 95% yield. ^1H NMR (400 MHz, CD_3CN) δ= 1.25 – 1.40 (m, 4H, 2x CH_2 hexylspacer), 1.40 – 1.51 (m, 2H, CH_2 hexylspacer), 1.58 – 1.71 (m, 2H, CH_2 hexylspacer), 1.80 – 1.92 (m, 12H, 3x 5a'-H, 3x *Ac*NH), 1.96 – 2.30 (m, 6H, 3x 5a-H, 3x 5-H), 2.68 – 2.83 (m, 6H, 3x CH_2 cyanoethyl), 2.93 (bs, 1H, OH), 3.00 – 3.11 (m, 2H, CH_2 hexylspacer), 3.59 – 3.89 (m, 9H, 3x H-6, 3x H-4), 3.96 – 4.22 (m, 11H, 3x H-3, CH_2 hexylspacer, 3x CH_2 cyanoethyl), 4.31 – 4.86 (m, 18H, 3x H-1, 3x H-2, 6x CH_2 Bn), 5.03 (s, 2H, CH_2 Bn spacer), 5.78 (bs, 1H, NH), 6.55 – 6.65 (m, 1H, N*H*Ac), 6.9 – 7.15 (m, 2H, 2x N*H*Ac), 7.19 – 7.40 (m, 35H, H_{arom}). ^{13}C NMR (100 MHz,CD_3CN) δ= 20.0 – 20.1 (3x CH_2 cyanoethyl), 22.9 – 23.0 (3x CH_3 *Ac*NH), 25.5 (CH_2 hexylspacer), 26.5 (CH_2 hexylspacer), 28.9 – 29.2 (3x CH_2 C-5a), 30.1 (CH_2 hexylspacer), 30.5 (CH_2 hexylspacer), 38.0 – 40.0 (3x CH C-5), 41.1 (CH_2 hexylspacer), 50.8 – 51.4 (3x CH C-2), 62.5 – 63.0 (3x CH_2 C-6), 63.0 – 63.3 (3x CH_2 cyanoethyl), 66.3 (CH_2 Bn spacer), 68.4 (CH_2 hexylspacer), 72.1 – 74.1 (6x CH_2 Bn), 75.2 – 75.5 (3x CH C-1), 75.5 – 76.1 (3x CH C-4), 79.3 – 79.5 (3x CH C-3), 128.2 – 129.1 (CH_{arom}), 138.9 – 139.7 (7x C_q Bn), 170.9 – 171.2 (3x C=O *Ac*NH). ^{31}P NMR (162 MHz, CD_3CN) δ= −2.82, −2.77, −2.62, −2.58, −2.36, −2.33, −2.24, −2.20, −2.16. HRMS: $[C_{92}H_{114}N_7O_{24}P_3 + H]^+$ calculated 1795.72333, found 1795.22333.

1-*O*-tetra-((2-Acetamido-3,4-di-*O*-benzyl-2-deoxy-5a-carba-α-D-mannopyranosyl-1-*O*-phosphoryl)2-cyanoethyl)-6-hexyl-benzyl-carbamate (18). Alcohol **17** (0.267 g, 0.148 mmol), was coupled to phosphoramidite **13** (1.4 mL 0.16 M in ACN, 0.22 mmol), oxidized, detritylated using the general procedure as described above. The crude was purified by size exclusion chromatography (Sephadex LH-20, DCM/MeOH 1:1) leading to product **18** (0.299 g, 0.129 mmol) in 87% yield. ^1H NMR (400 MHz, $(CD_3)_2CO$) δ= 1.31 – 1.47 (m, 4H, 2x CH_2 hexylspacer), 1.47 – 1.57 (m, 2H, CH_2 hexylspacer), 1.62 – 1.75 (m, 2H, CH_2 hexylspacer), 1.85 – 2.02 (m, 16H, 4x 5a'-H, 4x *Ac*NH), 2.07 – 2.17 (m, 8H, 4x 5a-H, 4x 5-H), 2.82 – 3.00 (m, 8H, 4x CH_2 cyanoethyl), 3.08 – 3.18 (m, 2H, CH_2 hexylspacer), 3.66 – 4.01 (m, 12H, 4x H-6, 4x H-4), 4.04 – 4.36 (m, 14H, 4x H-3, CH_2 hexylspacer, 4x CH_2 cyanoethyl), 4.40 – 4.94 (m, 24H, 4x H-1, 4x H-2, 8x CH_2 Bn), 5.05 (s, 2H, CH_2 Bn spacer), 6.39 (bs, 1H, NH), 7.17 – 7.42 (m, 45H, H_{arom}), 7.42 – 7.80 (m, 4H, N*H*Ac). ^{13}C NMR (100 MHz, $(CD_3)_2CO$) δ= 20.0 – 20.1 (4x CH_2 cyanoethyl), 23.1 – 23.2 (4x CH_3 *Ac*NH), 25.8 (CH_2 hexylspacer), 26.8 (CH_2 hexylspacer), 29.2 – 29.8 (4x CH_2 C-5a), 30.8 (CH_2 hexylspacer), 30.8 (CH_2 hexylspacer), 38.3 – 40.3 (4x CH C-5), 41.4 (CH_2 hexylspacer), 51.2 – 51.5 (4x CH C-2), 62.6 – 63.4 (4x CH_2 C-6), 63.4 – 63.6 (4x CH_2 cyanoethyl), 66.2 (CH_2 Bn spacer), 68.8 (CH_2 hexylspacer), 72.0 – 75.0 (8x CH_2 Bn), 75.6 – 75.8 (4x CH C-1), 76.5 – 77.2 (4x CH C-4), 79.7 – 79.8 (4x CH C-3), 128.1 – 129.1 (CH_{arom}), 139.3 – 140.1 (9x C_q Bn), 170.7 – 171.2 (4x C=O *Ac*NH). ^{31}P NMR (162 MHz, $CD_3)_2CO$) δ= −2.84, −2.77, −2.68, −2.47, −2.42, −2.37, −2.30, −1.96, −1.91, −1.89. HRMS: $[C_{118}H_{145}N_9O_{31}P_4 + 2H]^{++}$ calculated 1155.45892, found 1155.45892

1-*O*-penta-((2-Acetamido-3,4-di-*O*-benzyl-2-deoxy-5a-carba-α-D-mannopyranosyl-1-*O*-phosphoryl)2-cyanoethyl)-6-hexyl-benzyl-carbamate (19). Alcohol **18** (0.277 g, 0.120 mmol), was coupled to phosphoramidite **13** (1.1 mL 0.16 M in ACN, 0.18 mmol), oxidized, detritylated using the general procedure as described above. The crude was purified by size exclusion chromatography (Sephadex LH-20, DCM/MeOH 1:1) leading to product **19** (0.31 g, 0.110 mmol) in 92% yield. ^{1}H NMR (400 MHz, $(CD_3)_2CO$) δ= 1.31 – 1.46 (m, 4H, 2x CH_2 hexylspacer), 1.46 – 1.58 (m, 2H, CH_2 hexylspacer), 1.62 – 1.75 (m, 2H, CH_2 hexylspacer), 1.84 – 2.02 (m, 20H, 5x 5a'-H, 5x *Ac*NH), 2.07 – 2.19 (m, 10H, 5x 5a-H, 5x 5-H), 2.82 – 2.97 (m, 10H, 5x CH_2 cyanoethyl), 3.08 – 3.18 (m, 2H, CH_2 hexylspacer), 3.67 – 4.02 (m, 15H, 5x H-6, 5x H-4), 4.04 – 4.36 (m, 17H, 5x H-3, CH_2 hexylspacer, 5x CH_2 cyanoethyl), 4.38 – 4.95 (m, 30H, 5x H-1, 5x H-2, 10x CH_2 Bn), 5.05 (s, 2H, CH_2 Bn spacer), 6.43 (bs, 1H, NH), 7.16 – 7.41 (m, 55H, H_{arom}), 7.42 – 7.86 (m, 5H, N*H*Ac). ^{13}C NMR (100 MHz, $(CD_3)_2CO$) δ= 19.8 – 20.0 (5x CH_2 cyanoethyl), 23.0 – 23.1 (5x CH_3 *Ac*NH), 25.7 (CH_2 hexylspacer), 26.7 (CH_2 hexylspacer), 29.2 – 30.0 (5x CH_2 C-5a), 30.7 (CH_2 hexylspacer), 30.7 (CH_2 hexylspacer), 38.2 – 40.2 (5x CH C-5), 41.2 (CH_2 hexylspacer), 51.0 – 51.4 (5x CH C-2), 62.5 – 63.2 (5x CH_2 C-6), 63.3 – 63.5 (5x CH_2 cyanoethyl), 66.1 (CH_2 Bn spacer), 68.7 (CH_2 hexylspacer), 72.0 – 75.0 (10x CH_2 Bn), 75.6 – 75.8 (5x CH C-1), 76.5 – 77.2 (5x CH C-4), 79.7 – 79.8 (5x CH C-3), 128.0 – 129.0 (CH_{arom}), 139.2 – 140.0 (11x C_q Bn), 170.7 – 171.2 (5x C=O *Ac*NH). ^{31}P NMR (162 MHz, $CD_3)_2CO$) δ= –2.84, –2.77, –2.68, –2.47, –2.42, –2.37, –2.30, –1.96, –1.88, –1.89, –1.86, –1.84, –1.79. HRMS: $[C_{144}H_{176}N_{11}O_{38}P_5 + 2H]^{++}$ calculated 1412.55219, found 1412.55219.

1-*O*-hexa-((2-Acetamido-3,4-di-*O*-benzyl-2-deoxy-5a-carba-α-D-mannopyranosyl-1-*O*-phosphoryl)2-cyanoethyl)-6-hexyl-benzyl-carbamate (20). Alcohol **19** (0.280 g, 0.099 mmol), was coupled to phosphoramidite **13** (1.24 mL 0.16 M in ACN, 0.20 mmol), oxidized, detritylated using the general procedure as described above. The crude was purified by size exclusion chromatography (Sephadex LH-20, DCM/MeOH 1:1) leading to product **20** (0.29 g, 0.087 mmol) in 88% yield. ^{1}H NMR (500 MHz, $(CD_3)_2CO$) δ= 1.31 – 1.46 (m, 4H, 2x CH_2 hexylspacer), 1.46 – 1.57 (m, 2H, CH_2 hexylspacer), 1.63 – 1.74 (m, 2H, CH_2 hexylspacer), 1.84 – 2.02 (m, 24H, 6x 5a'-H, 6x *Ac*NH), 2.07 – 2.30 (m, 12H, 6x 5a-H, 6x 5-H), 2.82 – 2.97 (m, 12H, 6x CH_2 cyanoethyl), 3.09 – 3.18 (m, 2H, CH_2 hexylspacer), 3.67 – 4.04 (m, 18H, 6x H-6, 6x H-4), 4.04 – 4.38 (m, 20H, 6x H-3, CH_2 hexylspacer, 6x CH_2 cyanoethyl), 4.38 – 5.00 (m, 36H, 6x H-1, 6x H-2, 12x CH_2 Bn), 5.05 (s, 2H, CH_2 Bn spacer), 6.42 (bs, 1H, NH), 7.16 – 7.41 (m, 65H, H_{arom}), 7.42 – 7.89 (m, 6H, N*H*Ac). ^{13}C NMR (100 MHz, $(CD_3)_2CO$) δ= 19.9 – 20.0 (6x CH_2 cyanoethyl), 23.0 – 23.1 (6x CH_3 *Ac*NH), 25.7 (CH_2 hexylspacer), 26.8 (CH_2 hexylspacer), 29.2 – 30.2 (6x CH_2 C-5a), 30.4 (CH_2 hexylspacer), 30.7 (CH_2 hexylspacer), 38.2 – 40.2 (6x CH C-5), 41.3 (CH_2 hexylspacer), 51.0 – 51.4 (6x CH C-2), 62.5 – 63.4 (6x CH_2 C-6), 63.4 – 63.5 (6x CH_2 cyanoethyl), 66.2 (CH_2 Bn spacer), 68.7 (CH_2 hexylspacer), 72.2 – 75.6 (12x CH_2 Bn), 75.6 – 75.8 (6x CH C-1), 76.5 – 77.2 (6x CH C-4), 79.7 – 79.8 (6x CH C-3), 128.1 – 129.1 (CH_{arom}), 139.2 – 140.0 (13x C_q Bn), 170.7 – 171.2 (6x C=O *Ac*NH). ^{31}P NMR (162 MHz, $CD_3)_2CO$) δ= –2.84, –2.77, –2.68, –2.45, –2.42, –2.37, –2.31, –1.94, –1.81, –1.78. HRMS: $[C_{170}H_{207}N_{13}O_{45}P_6 + NH_4]^{+}$ calculated 3356.312, found 3357.010.

1-*O*-epta-((2-Acetamido-3,4-di-*O*-benzyl-2-deoxy-5a-carba-α-D-mannopyranosyl-1-*O*-phosphoryl)2-cyanoethyl)-6-hexyl-benzyl-carbamate (21). Alcohol **20** (0.140 g, 0.042 mmol), was coupled to phosphoramidite **13** (0.8 mL 0.1 M in ACN, 0.84 mmol), oxidized, detritylated using the general procedure as described above. The crude was purified by size exclusion chromatography (Sephadex LH-20, DCM/MeOH 1:1) leading to product **21** (0.139 g, 0.036 mmol) in 86% yield. ^{1}H NMR (500 MHz, $(CD_3)_2CO$) δ= 1.31 – 1.46 (m, 4H, 2x CH_2 hexylspacer), 1.46 – 1.57 (m, 2H, CH_2 hexylspacer), 1.63 – 1.74 (m, 2H, CH_2 hexylspacer), 1.84 – 2.02 (m, 28H, 7x 5a'-H, 7x *Ac*NH), 2.07 – 2.30 (m, 14H, 7x 5a-H, 7x 5-H), 2.82 – 2.97 (m, 14H, 7x CH_2 cyanoethyl), 3.09 – 3.18 (m, 2H, CH_2 hexylspacer), 3.67 – 4.04 (m, 21H, 7x H-6, 7x H-4), 4.04 – 4.38 (m, 23H, 7x H-3, CH_2 hexylspacer, 7x CH_2 cyanoethyl), 4.38 – 5.00 (m, 42H, 7x H-1, 7x H-2, 14x CH_2 Bn), 5.05 (s, 2H, CH_2 Bn spacer), 6.42 (bs, 1H, NH), 7.16 – 7.41 (m, 75H, H_{arom}), 7.42 – 7.89 (m, 7H, N*H*Ac). ^{13}C NMR (125

MHz, $(CD_3)_2CO$) δ= 19.9 – 20.0 (7x CH_2 cyanoethyl), 23.0 – 23.1 (7x CH_3 *Ac*NH), 25.7 (CH_2 hexylspacer), 26.8 (CH_2 hexylspacer), 29.2 – 30.2 (7x CH_2 C-5a), 30.4 (CH_2 hexylspacer), 30.7 (CH_2 hexylspacer), 38.2 – 40.2 (7x CH C-5), 41.3 (CH_2 hexylspacer), 51.0 – 51.4 (7x CH C-2), 62.5 – 63.4 (7x CH_2 C-6), 63.4 – 63.5 (7x CH_2 cyanoethyl), 66.2 (CH_2 Bn spacer), 68.7 (CH_2 hexylspacer), 72.2 – 75.6 (14x CH_2 Bn), 75.6 – 75.8 (7x CH C-1), 76.5 – 77.2 (7x CH C-4), 79.7 – 79.8 (7x CH C-3), 128.1 – 129.1 (CH_{arom}), 139.2 – 140.0 (15x C_q Bn), 170.7 – 171.2 (7x C=O *Ac*NH). ^{31}P NMR (202 MHz, $CD_3)_2CO$) δ= –2.84, –2.77, –2.68, –2.45, –2.42, –2.37, –2.31, –1.94, –1.81, –1.78. HRMS: $[C_{196}H_{238}N_{15}O_{52}P_7 + 2H]^{++}$ calculated 1926,73908, found 1926,73908.

1-*O*-octa-((2-Acetamido-3,4-di-*O*-benzyl-2-deoxy-5a-carba-α-D-mannopyranosyl-1-*O*-phosphoryl)2-cyanoethyl)-6-hexyl-benzyl-carbamate (22). Alcohol **22** (0.105 g, 0.027 mmol), was coupled to phosphoramidite **13** (0.7 mL 0.1 M in ACN, 0.68 mmol), oxidized, detritylated using the general procedure as described above. The crude was purified by size exclusion chromatography (Sephadex LH-20, DCM/MeOH 1:1) leading to product **22** (0.103 g, 0.023 mmol) in 87% yield. ^1H NMR (500 MHz, $(CD_3)_2CO$) δ= 1.31 – 1.46 (m, 4H, 2x CH_2 hexylspacer), 1.46 – 1.57 (m, 2H, CH_2 hexylspacer), 1.63 – 1.74 (m, 2H, CH_2 hexylspacer), 1.84 – 2.02 (m, 32H, 8x 5a'-H, 8x *Ac*NH), 2.07 – 2.30 (m, 16H, 8x 5a-H, 8x 5-H), 2.82 – 2.97 (m, 16H, 8x CH_2 cyanoethyl), 3.09 – 3.18 (m, 2H, CH_2 hexylspacer), 3.67 – 4.04 (m, 24H, 8x H-6, 8x H-4), 4.04 – 4.38 (m, 26H, 8x H-3, CH_2 hexylspacer, 8x CH_2 cyanoethyl), 4.38 – 5.00 (m, 48H, 8x H-1, 8x H-2, 16x CH_2 Bn), 5.05 (s, 2H, CH_2 Bn spacer), 6.42 (bs, 1H, NH), 7.16 – 7.41 (m, 85H, H_{arom}), 7.42 – 7.89 (m, 8H, N*H*Ac). ^{13}C NMR (125 MHz, $(CD_3)_2CO$) δ= 19.9 – 20.0 (8x CH_2 cyanoethyl), 23.0 – 23.1 (8x CH_3 *Ac*NH), 25.7 (CH_2 hexylspacer), 26.8 (CH_2 hexylspacer), 29.2 – 30.2 (8x CH_2 C-5a), 30.4 (CH_2 hexylspacer), 30.7 (CH_2 hexylspacer), 38.2 – 40.2 (8x CH C-5), 41.3 (CH_2 hexylspacer), 51.0 – 51.4 (8x CH C-2), 62.5 – 63.4 (8x CH_2 C-6), 63.4 – 63.5 (8x CH_2 cyanoethyl), 66.2 (CH_2 Bn spacer), 68.7 (CH_2 hexylspacer), 72.2 – 75.6 (16x CH_2 Bn), 75.6 – 75.8 (8x CH C-1), 76.5 – 77.2 (8x CH C-4), 79.7 – 79.8 (8x CH C-3), 128.1 – 129.1 (CH_{arom}), 139.2 – 140.0 (17x C_q Bn), 170.7 – 171.2 (8x C=O *Ac*NH). ^{31}P NMR (202 MHz, $CD_3)_2CO$) δ= –2.84, –2.77, –2.68, –2.45, –2.42, –2.37, –2.31, –1.94, –1.81, –1.78. HRMS: $[C_{222}H_{269}N_{17}O_{59}P_8 + 2H]^{++}$ calculated 2184.33410, found 2184.33410.

1-*O*-(2-Acetamido-2-deoxy-5a-carba-α-D-mannopyranosyl-1-*O*-phosphoryl)-6-hexyl-amine (1). Alcohol **15** (34 µmol) was deprotected using the general procedure described above. The pure oligomer **1** was obtained in 99% yield (14,3 mg, 34,0 µmol). ^1H NMR (500 MHz, D_2O) δ= 1.33 – 1.43 (m, 4H, 2x CH_2 hexylspacer), 1.55 – 1.68 (m, 4H, 2x CH_2 hexylspacer), 1.84 – 1.96 (m, 3H, 5a'-H, 5a-H, 5-H), 1.96 – 2.02 (s, 3H, *Ac*NH), 2.91 – 2.99 (m, 2H, CH_2 hexylspacer), 3.47 – 3.55 (m, 1H, H-4), 3.64 – 3.74 (m, 2H, CH_2 hexylspacer), 3.82 – 3.92 (m, 3H, H-3, H-6), 4.22 – 4.29 (m, 1H, H-1), 4.34 – 4.40 (m, 1H, H-2). ^{13}C NMR (126 MHz, D_2O) δ= 21.9 (CH_3 *Ac*NH), 24.4 (CH_2 hexylspacer), 25.1 (CH_2 hexylspacer), 26.6 (CH_2 hexylspacer), 28.0 (CH_2 C-5a), 29.5 (CH_2 hexylspacer), 38.6 (CH C-5), 39.4 (CH_2 hexylspacer), 53.5 (CH C-2), 61.9 (CH_2 C-6), 66.2 (CH_2 hexylspacer), 70.1 (CH C-1), 70.4 (CH C-4), 71.9 (CH C-3), 174.7 (C=O *Ac*NH). ^{31}P NMR (202 MHz, D_2O) δ= 0.25. HRMS: $[C_{15}H_{31}N_2O_8P + H]^+$ calculated 399.18908, found 399.18908.

1-*O*-di-(2-Acetamido-2-deoxy-5a-carba-α-D-mannopyranosyl-1-*O*-phosphoryl)-6-hexyl-amine (2). Alcohol **16** (21 µmol) was deprotected using the general procedure described above. The pure oligomer **2** was obtained in 76% yield (11.6 mg, 16 µmol). ^1H NMR (500 MHz, D_2O) δ= 1.33 – 1.43 (m, 4H, 2x CH_2 hexyl spacer), 1.57 – 1.69 (m, 4H, 2x CH_2 hexylspacer), 1.72 – 1.96 (m, 6H, 2x 5a'-H, 2x 5a-H, 2x 5-H), 1.96 – 2.06 (s, 6H, 2x *Ac*NH), 2.92 – 3.00 (m, 2H, CH_2 hexylspacer), 3.48 – 3.64 (m, 2H, 2x H-4), 3.65 – 3.76 (m, 2H, CH_2 hexylspacer), 3.82 – 4.14 (m, 6H, 2x H-3, 2x H-6), 4.22 – 4.33 (m, 2H, 2x H-1), 4.36 – 4.44 (m, 2H, 2x H-2).^{31}P NMR (202 MHz, D_2O) δ= 0.23, 0.38. HRMS: $[C_{24}H_{46}N_3O_{15}P_2 + H]^+$ calculated 680.25552, found 680.25552.

1-*O*-tri-(2-Acetamido-2-deoxy-5a-carba-α-D-mannopyranosyl-1-*O*-phosphoryl)-6-hexyl-amine (3). Alcohol **17** (17 µmol) was deprotected using the general procedure described above.

The pure oligomer **3** was obtained in 69% yield (12.0 mg, 11.7 µmol). ^{1}H NMR (500 MHz, D_2O) δ= 1.33 – 1.43 (m, 4H, 2x CH_2 hexylspacer), 1.57 – 1.69 (m, 4H, 2x CH_2 hexylspacer), 1.72 – 2.09 (m, 18H, 3x 5a'-H, 3x 5a-H, 3x 5-H, 3x *Ac*NH), 2.92 – 3.00 (m, 2H, CH_2 hexylspacer), 3.48 – 3.66 (m, 3H, 3x H-4), 3.66 – 3.76 (m, 2H, CH_2 hexylspacer), 3.80 – 4.18 (m, 9H, 3x H-3, 3x H-6), 4.23 – 4.34 (m, 3H, 3x H-1), 4.37 – 4.50 (m, 3H, 3x H-2).^{31}P NMR (202 MHz, D_2O) δ= 0.24, 0.36, 0.39 HRMS: $[C_{33}H_{63}N_4O_{22}P_3 + H]^+$ calculated 961.32196, found 961.32196.

1-*O*-tetra-(2-Acetamido-2-deoxy-5a-carba-α-D-mannopyranosyl-1-*O*-phosphoryl)-6-hexyl-amine (4). Alcohol **18** (8.9 µmol) was deprotected using the general procedure described above. The pure oligomer **4** was obtained in 39% yield (4.6 mg, 3.5 µmol). ^{1}H NMR (500 MHz, D_2O) δ= 1.33 – 1.43 (m, 4H, 2x CH_2 hexylspacer), 1.57 – 1.69 (m, 4H, 2x CH_2 hexylspacer), 1.74 – 2.07 (m, 24H, 4x 5a'-H, 4x 5a-H, 4x 5-H, 4x *Ac*NH), 2.92 – 3.00 (m, 2H, CH_2 hexylspacer), 3.48 – 3.66 (m, 3H, 4x H-4), 3.66 – 3.76 (m, 2H, CH_2 hexylspacer), 3.81 – 4.22 (m, 12H, 4x H-3, 4x H-6), 4.23 – 4.34 (m, 4H, 4x H-1), 4.38 – 4.50 (m, 4H, 4x H-2).^{31}P NMR (202 MHz, D_2O) δ= 0.25, 0.36, 0.40 HRMS: $[C_{42}H_{79}N_5O_{29}P_4 + H]^+$ calculated 1242.38839, found 1242.38830.

1-*O*-penta-(2-Acetamido-2-deoxy-5a-carba-α-D-mannopyranosyl-1-*O*-phosphoryl)-6-hexyl-amine (5). Alcohol **19** (11 µmol) was deprotected using the general procedure described above. The pure oligomer 5 was obtained in 88% yield (15.8 mg, 9.7 µmol). ^{1}H NMR (500 MHz, D_2O) δ= 1.33 – 1.43 (m, 4H, 2x CH_2 hexylspacer), 1.57 – 1.69 (m, 4H, 2x CH_2 hexylspacer), 1.74 – 2.07 (m, 30H, 5x 5a'-H, 5x 5a-H, 5x 5-H, 5x *Ac*NH), 2.92 – 3.00 (m, 2H, CH_2 hexylspacer), 3.47 – 3.68 (m, 5H, 5x H-4), 3.68 – 3.76 (m, 2H, CH_2 hexylspacer), 3.81 – 4.20 (m, 15H, 5x H-3, 5x H-6), 4.23 – 4.34 (m, 5H, 5x H-1), 4.37 – 4.50 (m, 5H, 5x H-2).^{31}P NMR (202 MHz, D_2O) δ= 0.25, 0.37, 0.41, 0.43. HRMS: $[C_{51}H_{95}N_6O_{36}P_5 + H]^+$ calculated 1523.45483, found 1523.45483.

1-*O*-hexa-(2-Acetamido-2-deoxy-5a-carba-α-D-mannopyranosyl-1-*O*-phosphoryl)-6-hexyl-amine (6). Alcohol **20** (21 µmol) was deprotected using the general procedure described above. The pure oligomer **6** was obtained in 83% yield (33.9 mg, 17.5 µmol). ^{1}H NMR (500 MHz, D_2O) δ= 1.33 – 1.43 (m, 4H, 2x CH_2 hexylspacer), 1.57 – 1.69 (m, 4H, 2x CH_2 hexylspacer), 1.71 – 2.08 (m, 36H, 6x 5a'-H, 6x 5a-H, 6x 5-H, 6x *Ac*NH), 2.92 – 3.00 (m, 2H, CH_2 hexylspacer), 3.48 – 3.68 (m, 6H, 6x H-4), 3.68 – 3.76 (m, 2H, CH_2 hexylspacer), 3.81 – 4.21 (m, 18H, 6x H-3, 6x H-6), 4.23 – 4.34 (m, 6H, 6x H-1), 4.37 – 4.50 (m, 6H, 6x H-2). ^{13}C NMR (126 MHz, D_2O) δ= 21.9 (6x CH_3 *Ac*NH), 24.4 (CH_2 hexylspacer), 25.1 (CH_2 hexylspacer), 26.6 (CH_2 hexylspacer), 28.0 (6x CH_2 C-5a), 29.5 (CH_2 hexylspacer), 38.6 (6x CH C-5), 39.4 (CH_2 hexylspacer), 53.5 (6x CH C-2), 61.9 (6x CH_2 C-6), 66.2 (CH_2 hexylspacer), 70.1 (6x CH C-1), 70.4 (6x CH C-4), 71.9 (6x CH C-3), 174.7 (6x C=O *Ac*NH). ^{31}P NMR (202 MHz, D_2O) δ= 0.25, 0.38, 0.42, 0.44, 0.47. HRMS: $[C_{60}H_{111}N_7O_{43}P_6 + 2H]^{++}$ calculated 902.76427, found 902.76427.

1-*O*-epta-(2-Acetamido-2-deoxy-5a-carba-α-D-mannopyranosyl-1-*O*-phosphoryl)-6-hexyl-amine (7). Alcohol **21** (5.9 µmol) was deprotected using the general procedure described above. The pure oligomer **7** was obtained in 77% yield (10.4 mg, 4.6 µmol). ^{1}H NMR (500 MHz, D_2O) δ= 1.33 – 1.43 (m, 4H, 2x CH_2 hexylspacer), 1.57 – 1.69 (m, 4H, 2x CH_2 hexylspacer), 1.73 – 2.08 (m, 42H, 7x 5a'-H, 7x 5a-H, 7x 5-H, 7x *Ac*NH), 2.92 – 3.00 (m, 2H, CH_2 hexylspacer), 3.48 – 3.68 (m, 7H, 7x H-4), 3.68 – 3.76 (m, 2H, CH_2 hexylspacer), 3.81 – 4.22 (m, 21H, 7x H-3, 7x H-6), 4.25 – 4.36 (m, 7H, 7x H-1), 4.37 – 4.53 (m, 7H, 7x H-2).^{31}P NMR (202 MHz, D_2O) δ= 0.25, 0.36, 0.41, 0.44, 0.47. HRMS: $[C_{69}H_{127}N_8O_{50}P_7 + H]^+$ calculated 2085.58771, found 2085.58771.

1-*O*-octa-(2-Acetamido-2-deoxy-5a-carba-α-D-mannopyranosyl-1-*O*-phosphoryl)-6-hexyl-amine (8). Alcohol **22** (23.2 µmol) was deprotected using the general procedure described above. The pure oligomer **8** was obtained in 44% yield (25.9 mg, 10.2 µmol). ^{1}H NMR (500 MHz, D_2O) δ= 1.33 – 1.43 (m, 4H, 2x CH_2 hexylspacer), 1.57 – 1.69 (m, 4H, 2x CH_2 hexylspacer), 1.73 – 2.08 (m, 48H, 8x 5a'-H, 8x 5a-H, 8x 5-H, 8x *Ac*NH), 2.92 – 3.00 (m, 2H, CH_2 hexylspacer), 3.48 – 3.68 (m, 8H,

8x H-4), 3.68 – 3.76 (m, 2H, CH_2 hexylspacer), 3.81 – 4.22 (m, 24H, 8x H-3, 8x H-6), 4.25 – 4.36 (m, 8H, 8x H-1), 4.37 – 4.53 (m, 8H, 8x H-2). ^{13}C NMR (126 MHz, D_2O) δ= 21.9 (8x CH_3 *Ac*NH), 24.4 (CH_2 hexylspacer), 25.1 (CH_2 hexylspacer), 26.6 (CH_2 hexylspacer), 28.0 (8x CH_2 C-5a), 29.5 (CH_2 hexylspacer), 38.6 (8x CH C-5), 39.4 (CH_2 hexylspacer), 53.5 (8x CH C-2), 61.9 (8x CH_2 C-6), 66.2 (CH_2 hexylspacer), 70.1 (8x CH C-1), 70.4 (8x CH C-4), 71.9 (8x CH C-3), 174.7 (8x C=O *Ac*NH). ^{31}P NMR (202 MHz, D_2O) δ= 0.25, 0.37, 0.41, 0.44, 0.48. HRMS: $[C_{78}H_{145}N_9O_{57}P_8 + H]^{++}$ calculated 1183.83071, found 1183.83071.

Stability of carba DP8 compared to Ac and DeOAc MenA avDP18

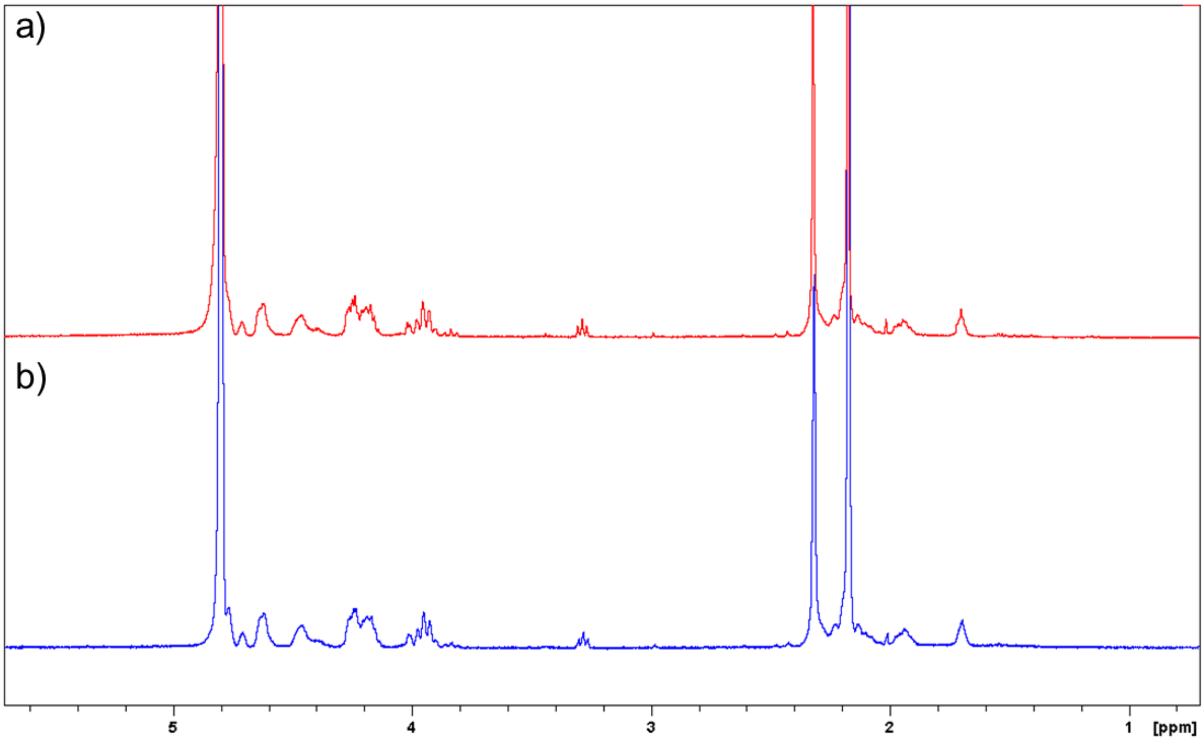

Supplementary Figure 1. ^1H NMR (D_2O, 400 MHz) of carba DP8 at day 0 (a) and 56 (b) in 5 mM NaOAc pH 7 at 37°C.

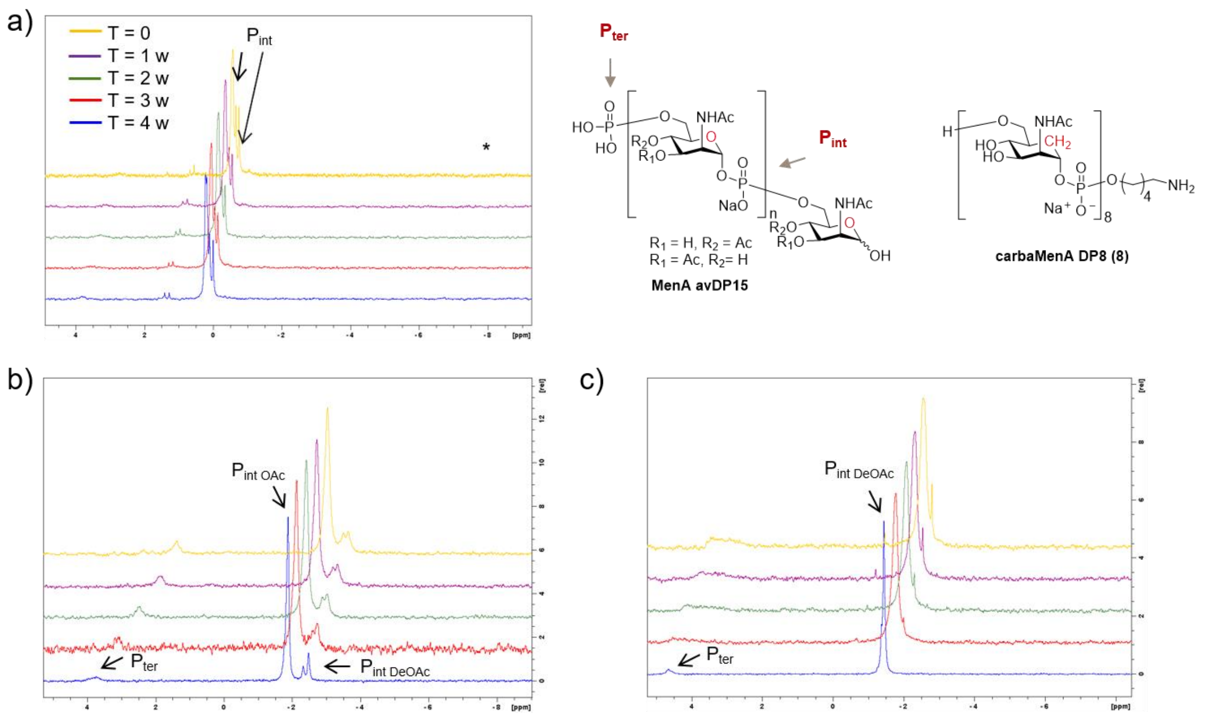

Supplementary Figure 2 ^{31}P NMR (D_2O, 162 MHz) of carba DP8 (a) and MenA avDP~15 with and without acetylation (b and c, respectively) at 37°C. Marked with * is inorganic phosphate contaminant.

In vitro evaluation of carbaMenA vs de-O-acetylated MenA CPS

Competitive ELISA analyses

Nunc MaxiSorp microtitre plates were coated with 100 µl per well of 5 µg mL^{-1} solution of native MenA polysaccharide in PBS buffer (0.15 M NaCl, 10 mM NaH_2PO_4, 2mM KCl, 4 mM KH_2PO_4) pH 8.2 and incubated overnight at 4°C.

The plates were washed tree times with 300 µL per wells with PBS buffer pH 7.2 with 0.05% Tween 20 (TPBS), blocked with 100 µL per well of TPBS buffer with 3% of BSA, incubated 1h at 37°C and washed again. The plate was designed to contain: a blank column with TPBS alone, without serum and inhibitors (b); a column with serum or mAb alone, without inhibitors (b0); the other columns contained both, the serum or the mAb and the inhibitors which included also the native MenA CPS and a not correlated polysaccharide as positive and negative controls respectively. The anti-MenA CPS monoclonal antibody JWA-1 (mIgG2a proprietary GSK, purified by Areta) was 1:5000 diluted (final concentration 35 µg mL^{-1}) in TPBS instead the polyclonal serum was 1:1000 for anti-MenA CPS and 1:800 for the anti-deOAc MenA, in the same buffer. Serial tenfold dilutions of the inhibitors were made in TPBS separately, starting from a concentration of 0.1 mM for native and deOAc MenA CPS and from 1 mM for avDP18, carba analogue molecules and negative control. 50 µL of pre-diluted mAb, were added to the coated plate and immediately mixed with 50 µL of inhibitor pre-diluted solution. The plates were incubated 2 h at 37°C. After washings, 100 µL per well of anti-mouse IgG conjugated to alkaline phosphatases (Sigma Aldrich) at the dilution of 1:10000 in TPBS were added, plates incubated 1h at 37°C and then washed.

ELISA development was done adding 100 µl per well of 1 M ethanolamine pH 9.8 containing 1 mg mL^{-1} of p-NPP (Sigma Aldrich). After 30 min of incubation, the plates were read at 405 nm using

a microplate reader (Spectramax 190). All OD lectures were subtracted of the mean value of the blank column (b). The inhibition percentage was expressed as follows:

$$\% \text{ inhibition} = [(B0 - ODx)/B0] * 100$$

Where B0 is the mean values of the b0 column and ODx is the optical density corresponding to each inhibitor concentration.

IC50 was defined as the inhibitor concentration resulting in 50% inhibition of the main reaction and were calculated by GraphPad Prism 7 software.

Competive ELISA against carba DP8 conjugate was done coating the carba DP8-CRM_{197} conjugate at 2 μg mL^{-1} concentration in term of protein, in pH 7.2 PBS buffer; anti-carba DP8 polyclonal serum was diluted at 1:400 and the inhibitors were diluted at the starting concentration of 0.3 mM in terms of saccharide for the conjugate, 0.1 mM for MenA CPS and negative control and 1 mM for the carba DP8. The experiment was then conducted as described above.

Supplementary Table 1. IC50 of carba DP8 compared to MenA CPS and avDP~15

	**anti MenA mAb**	**anti OAc MenA pAb**	**anti deOAc MenA pAb**
**Compound**	**IC_{50} mM**	**IC_{50} mM**	**IC_{50} mM**
MenA CPS	2.33×10^{-6}	5.59×10^{-6}	1.38×10^{-6}
MenA avDP~15	1.40×10^{-4}	5.03×10^{-3}	1.59×10^{-3}
Carba DP8	1.64×10^{-2}	0.24	1.59×10^{-2}

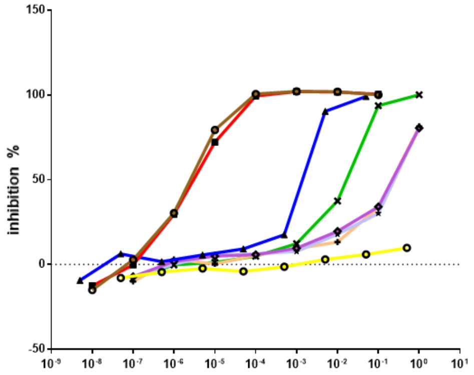

Supplementary Figure 3. Competitive ELISA of anti de-OAc MenA polyclonal serum using carbaMenA oligomers as inhibitors. DeOAc MenA-HSA conjugate was used for coating. MenA CPS and fragments thereof were the positive controls. Laminarin was the negative control. MenA CPS, red curve; MenA deOAc CPS, brown curve; MenA avDP~15, blue curve; carbaMenA DP4, dark purple curve; carbaMenA DP6 light purple curve; carbaMenA DP7, orange curve; carbaMenA DP8 green curve; negative control, yellow curve.

Characterization of the CarbaMenA neoglycoconjugates

MALDI-TOF mass analysis

The number of inserted carbohydrate moieties was determined by matrix-assisted laser desorption ionization time-of-flight mass spectrometry (MALDI-TOF MS; UltraFlex III MALDI-TOF/TOF instrument, BrukerDaltonics) in linear mode and with positive ion detection. The samples for analysis were prepared by mixing 2.5 μL of product and 2.5 μL of Super DHB matrix; 2.5 μL of each mixture was deposited on a samples plate, dried at room temperature for 10 min, and analyzed at the spectrometer.

Sodium Dodecyl Sulfate- Polyacrilammide gel electrophoresis (SDS-Page)

SDS-Page has been performed on pre-casted 3-8% polyacrylamide gels (NuPAGE Invitrogen). The electrophoretic runs have been performed in Tris-Acetate SDS running buffer (NuPAGE Invitrogen) loading 5 μg of protein for each sample, using the electrophoretic chamber with a voltage of 150V for about 40 minutes. Samples were prepared by adding 3 μl of NuPAGE LDS sample buffer. After electrophoretic running, the gel has been washed in H_2O for 3 times and then dye with comassie.

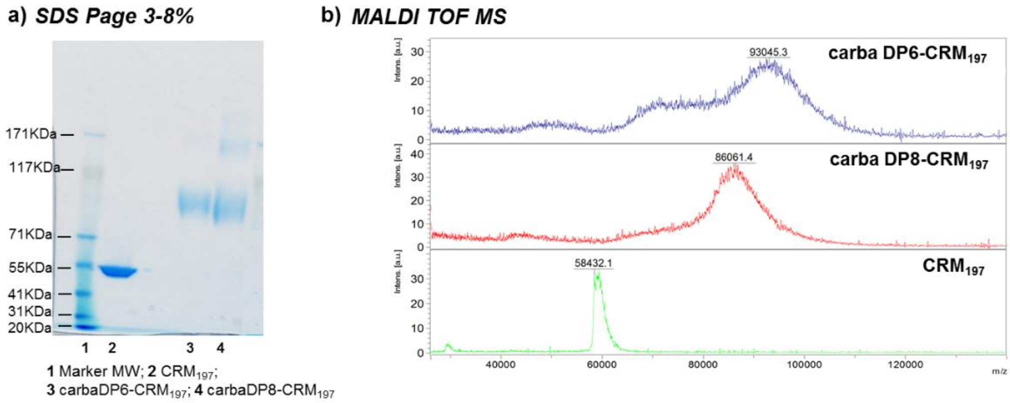

Supplementary Figure 4. a) SDS-page of the carbaMenA conjugates. b) MALDI-TOF MS spectra of the neoglycoconjugates.

Immunogenicity of Carba DP8 against itself

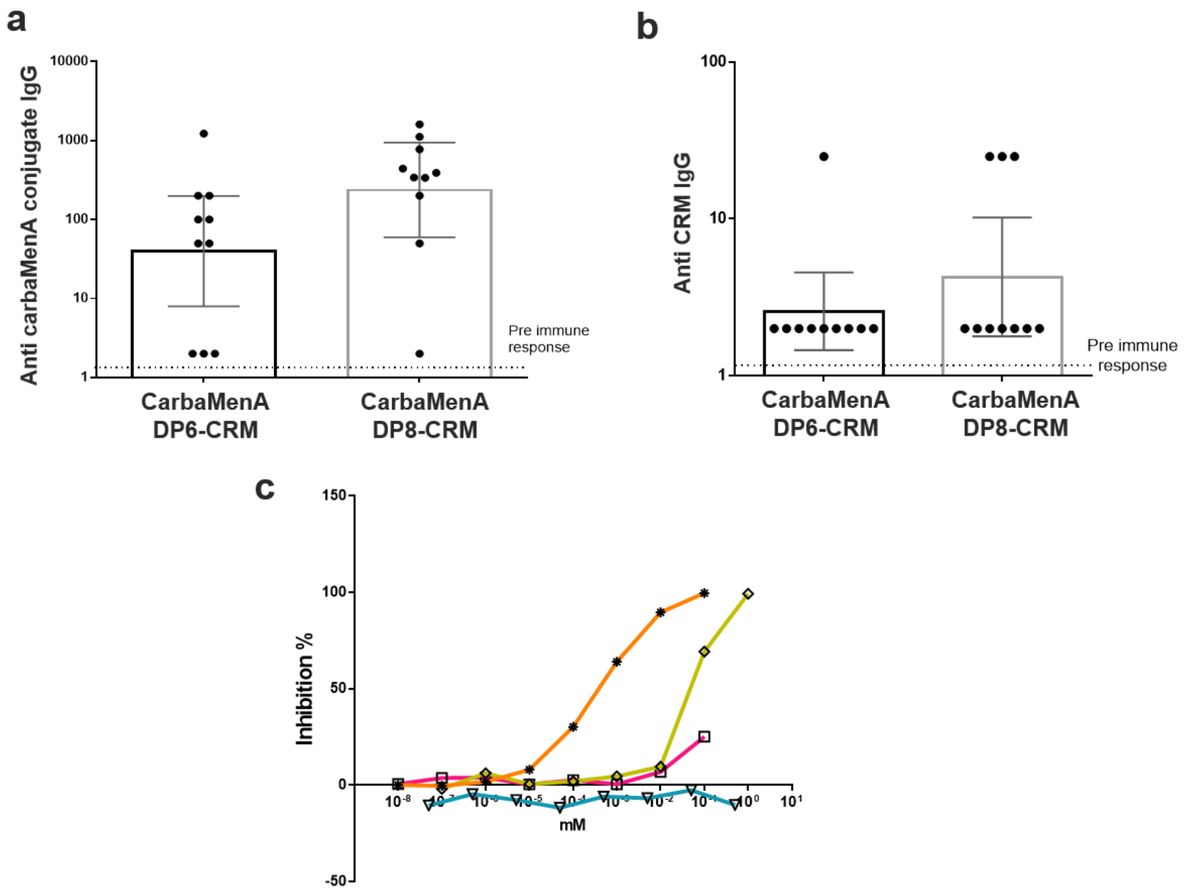

Supplementary Figure 5. Immune response elicited by the neo-glycoconjugates against itself. a-b) Graphs IgG titers reported as Geometric Mean (vertical bars) with the 95% of CI (vertical error bars) (n=10). c) Graph shows competitive ELISA results, coating carbaDP8 conjugate and using pooled serum from immunization with the same biomolecule. MenA CPS, magenta curve; carbaMenA DP8-CRM, orange curve; carbaMenA DP8 green; negative control, blue curve.

Synthesis of Ac-CarbaMenA DP8

Amine protection as Boc derivative (step 1, carbaMenA 8a). 10 mg of dried carba-analogue DP8 was solubilized in H_2O:dioxane 1:1 v/v (240 µL), then $NaHCO_3$ (2.95 eq) and $(Boc)_2O$ (1.13 eq) were added at 4°C. The reactions were then kept under magnetic stirring at room temperature overnight, then the products were purified by Sephadex G10 column (Eluent: H_2O) and fractions containing compound **8a** were dried and used for the next step.

Random O-acetylation (step 2, carbamenA 8b). The dried Boc protected carba-analogues from step 1 were resuspended in acetonitrile (110 µL), then acetic anhydride (3.6 eq for each -OH group in the molecule) and imidazole (1.8 eq) were added. The reactions were kept at 40 °C and the acetylation reaction time was extended until the target acetylation % (~75%) was reached (monitoring by ^{1}H-NMR). Then the crude acetylated compounds were dried. The level of incorporated acetylation in compound **8b** was quantified by ^{1}H NMR as shown in Supplementary Figure 57.

Boc deprotection (step 3, carbaMenA 8c). The dried crude *O*-acetylated carba-analogues from step 2 were solubilized in CH_2Cl_2:TFA 4:1 v/v (530 µL) and the reactions were kept under magnetic stirring at room temperature for 1 h. Then the crude reactions were dried, resolubilized in H_2O and purified by Sephadex G10 column (Eluent: MilliQ H_2O). The final samples were dried under vacuum, reconstituted in 0.6 mL D_2O and transferred to 5 mm NMR tubes. The compound **8c** identity and the level of random *O*-acetylation were assessed by ^{1}H NMR, collecting the spectra by a standard monodimensional pulseprogram at 400 MHz and 25 °C. The acquisition and processing were conducted by TopSpin Bruker software. The relative distribution of the acetyl groups between 3 and 4 positions was determined by ^{31}P NMR spectroscopy (101 MHz, D_2O) as shown in Supplementary Figure 58.

^{1}H NMR (400 MHz, D_2O) δ= 1.40 – 1.44 (m, 4H, 2x CH_2 hexylspacer), 1.63 – 1.70 (m, 4H, 2x CH_2 hexylspacer), 1.95 – 2.16 (m, 80H, , 8x 5a'-H, 8x 5a-H, 8x 5-H, 8x *Ac*NH, 8x Ac), 2.22-2.39 (m, 6H), 2.92 – 3.00 (t, *J* = 7.7 Hz, 2H, CH_2 hexylspacer), 3.49 – 3.74 (m, 2H), 3.81 – 3.99 (m, 18H), 4.01 – 4.27 (m, 5H), 4.29 – 4.44 (m, 8H, 8x H-1), 4.47 – 4.64 (m, 8H, 8x H-2), 5.01-5.27 (m, 12H, H-3,4).
^{31}P NMR (101 MHz, D_2O) δ= 0.03, -0.20, -0.38, -0.61.

SPR mAb competitive binding

Competitive binding assay were performed by SPR using a BIACORE X100 system. SA sensor chip (GE Healthcare, # BR-1000-32) was coated by standard SA-biotin capture method with biotinylated MenA CPS in flow cell (FC) 2 at the concentration of 25 μg mL^{-1} (targeting level of 200 RU). FC1 is used as reference. The immobilized surface density was ~350 resonance units. Binding analysis was performed with samples of anti-MenA CPS specific mAb (RP rec AB (mIgG2a) GSS2 produced by Biovest GmbH) at a fixed concentration (0.5 μg mL^{-1}) pre-incubated with MenA CPS or its fragments or carba-MenA fragments serially diluted (2x). Tested inhibitor concentration vary from 500 μg mL^{-1} to 0.05 ng mL^{-1}. Measurements were conducted in 10mM HEPES (pH 7.2), 150mM NaCl, 3mM EDTA, 0.005% Tween20 at 25°C and at a flow rate of 45 μL min^{-1}. Following mAb binding, conjugate surfaces were regenerated with 3.5 M $MgCl_2$ and a contact time of 120 s. Sensorgram data were analyzed using BIAevaluation software (Biacore).

Conjugation of Ac-CarbaMenA to CRM_{197}

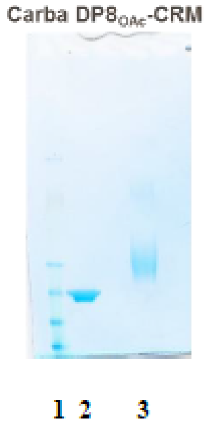

Supplementary Figure 5 SDS Page 3-8% TA: 1. Marker; 2. CRM_{197} (control); 3. Ac-carba DP8-CRM_{197}. Procedure for SDS Page in described in pg 12.

Supplementary Table 2. Summary Table of the conjugates used in this study

Conjugate	Saccharide/Protein w/w (mg mL^{-1})	Saccharide/Protein mol/mol
avDP15 MenA-CRM_{197}	0.53	6.3
avDP8.5 MenA-CRM_{197}	0.34	6.8
Ac-Carba DP8-CRM_{197}	0.13	2.5
Carba DP6-CRM_{197}	0.63	16.9
Carba DP8-CRM_{197}	0.50	10.5

Quantification of conjugated random Ac-CarbaMenA

For quantification of the conjugated carbaMenA, a HPAEC-PAD method was developed. The reported titers were obtained by hydrolyzing the samples with HCl at final concentration 4M at 110°C for 2 h in dry oven. After incubation samples were dried in as Speedvac system and then redissolved with water and filtered 0.45 µm. Quantification was performed by using a standard curve built in the range 0.5-4.0 µg mL^{-1} with CarbaMenA DP5, quantified by ^{1}H NMR 8sing an external maleic acid standard), and treated as samples. The analysis was performed on a ICS5000 system (Dionex-Themo Fisher) equipped with a CarboPac PA1 column with guard . Elution was made with a gradient of sodium acetate in presence of 100mM sodium hydroxide at 1.0mL/min and peak detected in pulse intregrated amperometry by using the quadruple wave form for carbohydrates. Results were elaborated with Chromeleon™ 7.2 Chromatography Data System (CDS) Software (SR4 version).

Supplementary Figures

NMR spectra of synthesized compounds

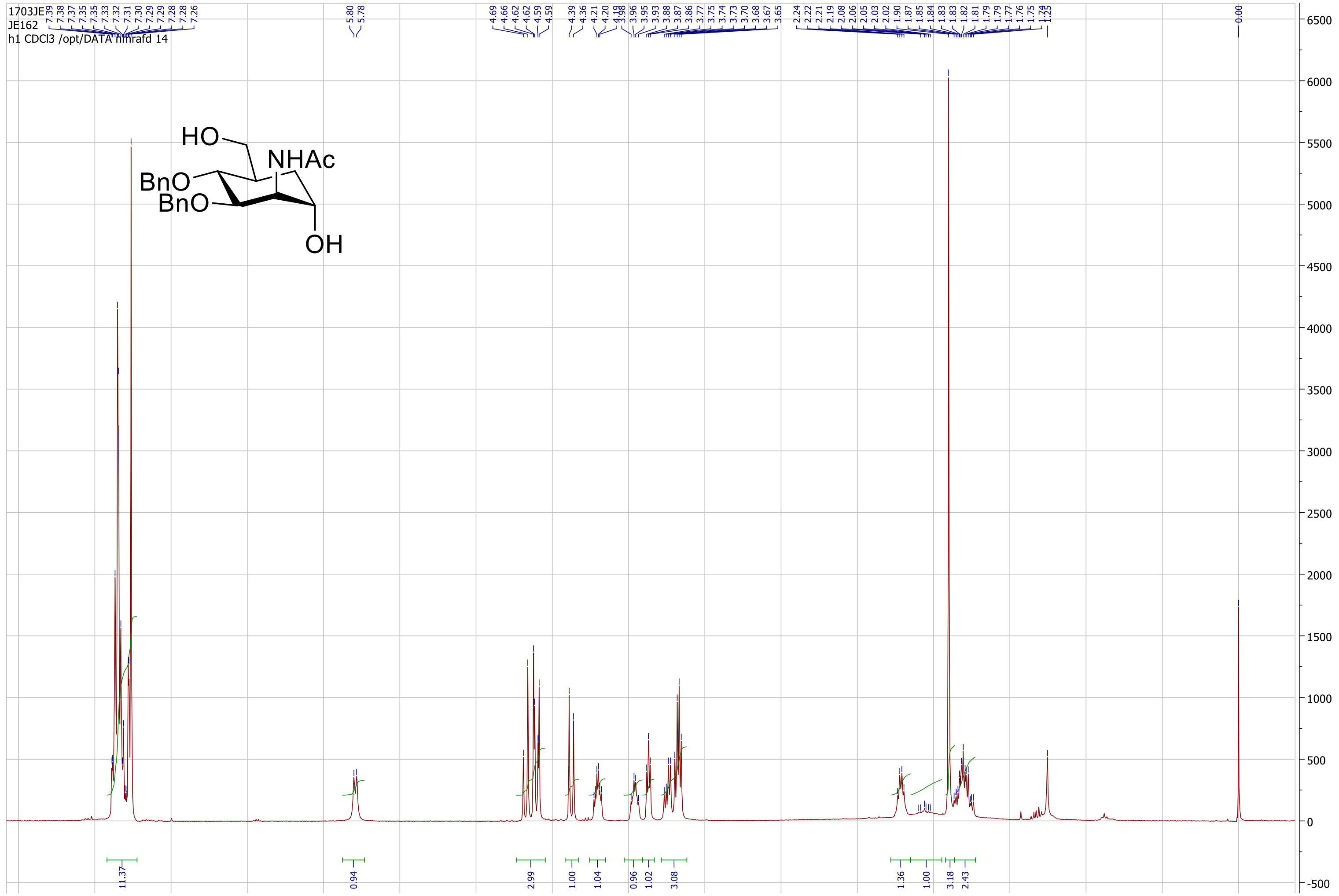

Supplementary Figure 6 2-Acetamido-3,4-di-O-benzyl-2-deoxy-6-O-thexyldimethylsilyl-5a-carba-α-D mannopyranose **11**

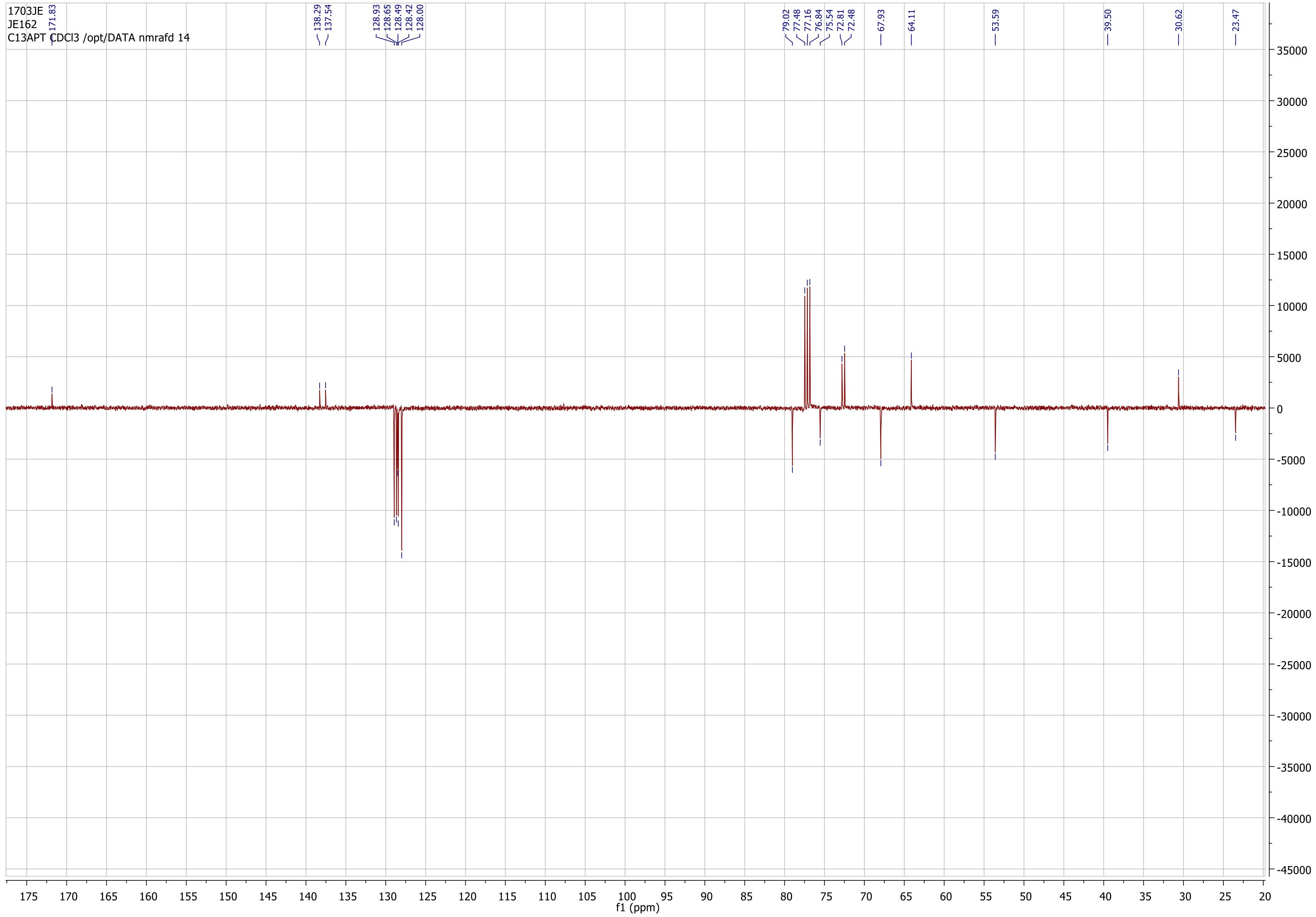

Supplementary Figure 7 2-Acetamido-3,4-di-O-benzyl-2-deoxy-6-O-thexyldimethylsilyl-5a-carba-α-D mannopyranose **11**

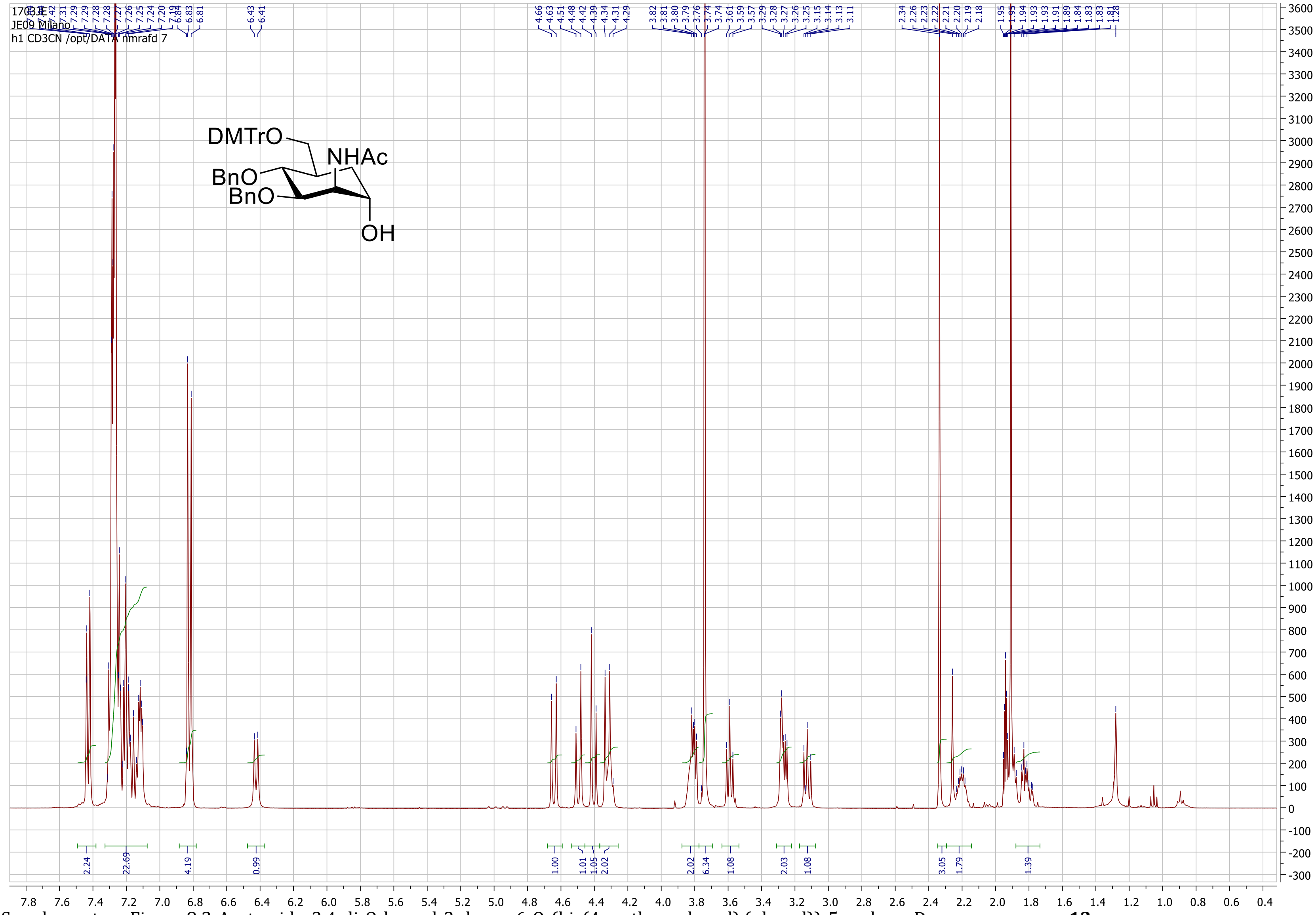

Supplementary Figure 8 2-Acetamido-3,4-di-O-benzyl-2-deoxy-6-O-(bis(4-methoxyphenyl) (phenyl))-5-carba-α-D-mannopyranose **12**

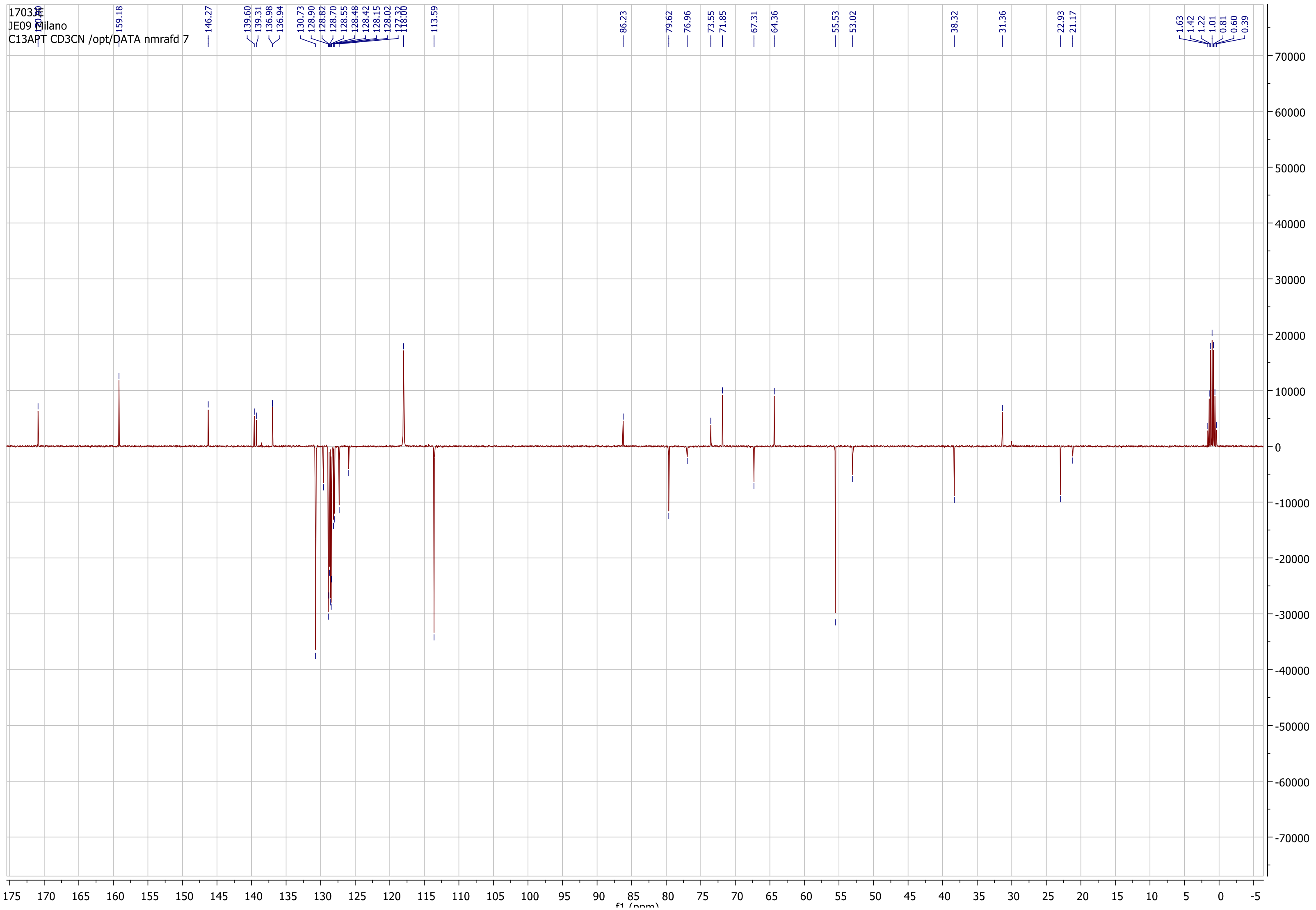

Supplementary Figure 9 2-Acetamido-3,4-di-O-benzyl-2-deoxy-6-O-(bis(4-methoxyphenyl) (phenyl))-5-carba-α-D-mannopyranose **12**

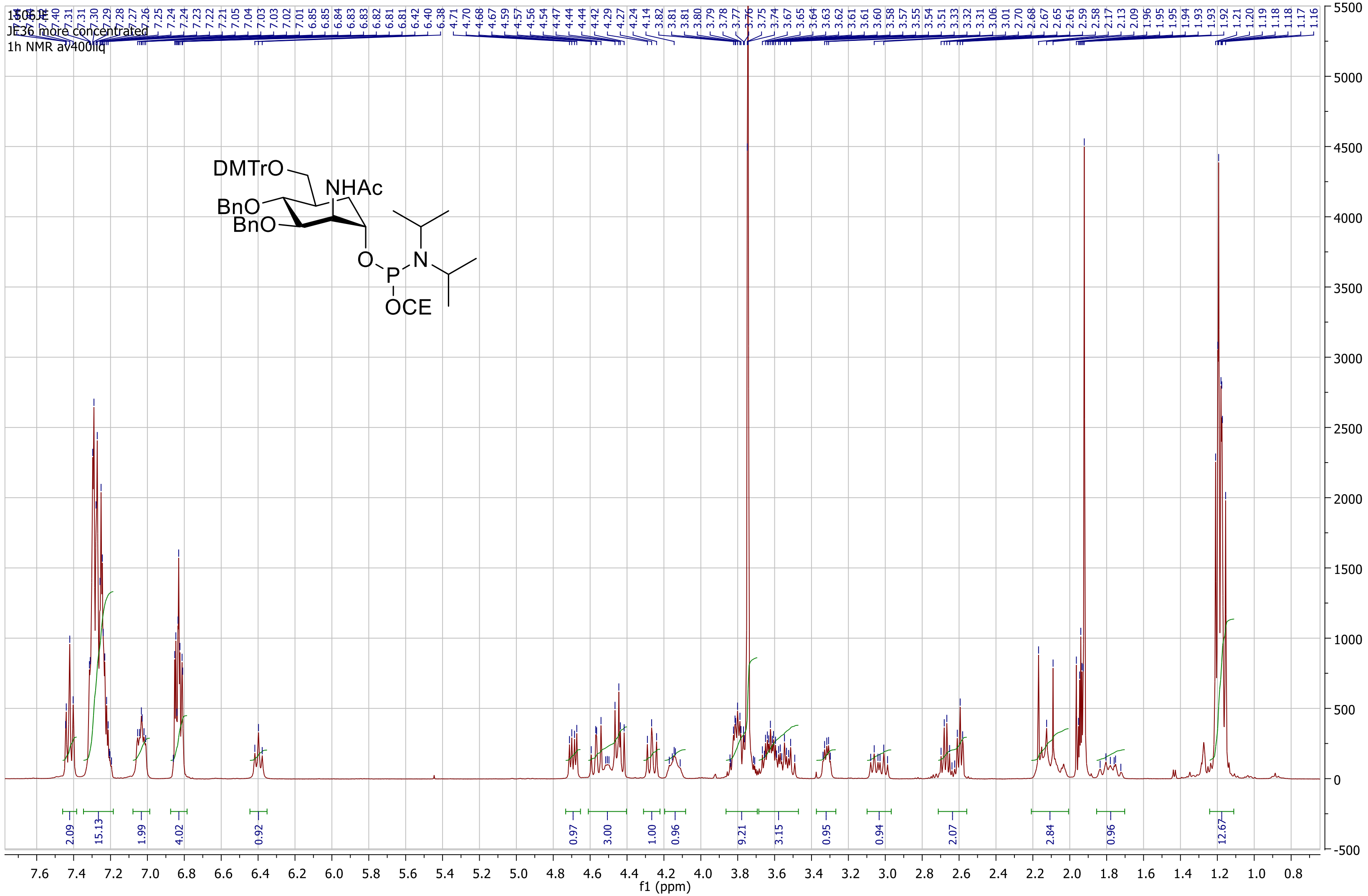

Supplementary Figure 10 1-O-((N,N-Diisopropylamino)-O-2-cyanoethyl-phosphoramidite))-2-Acetamido-3,4-di-O-benzyl-2-deoxy-6-O-(bis(4-methoxyphenyl)(phenyl))-5a-carba-α-D-mannopyranose **13**

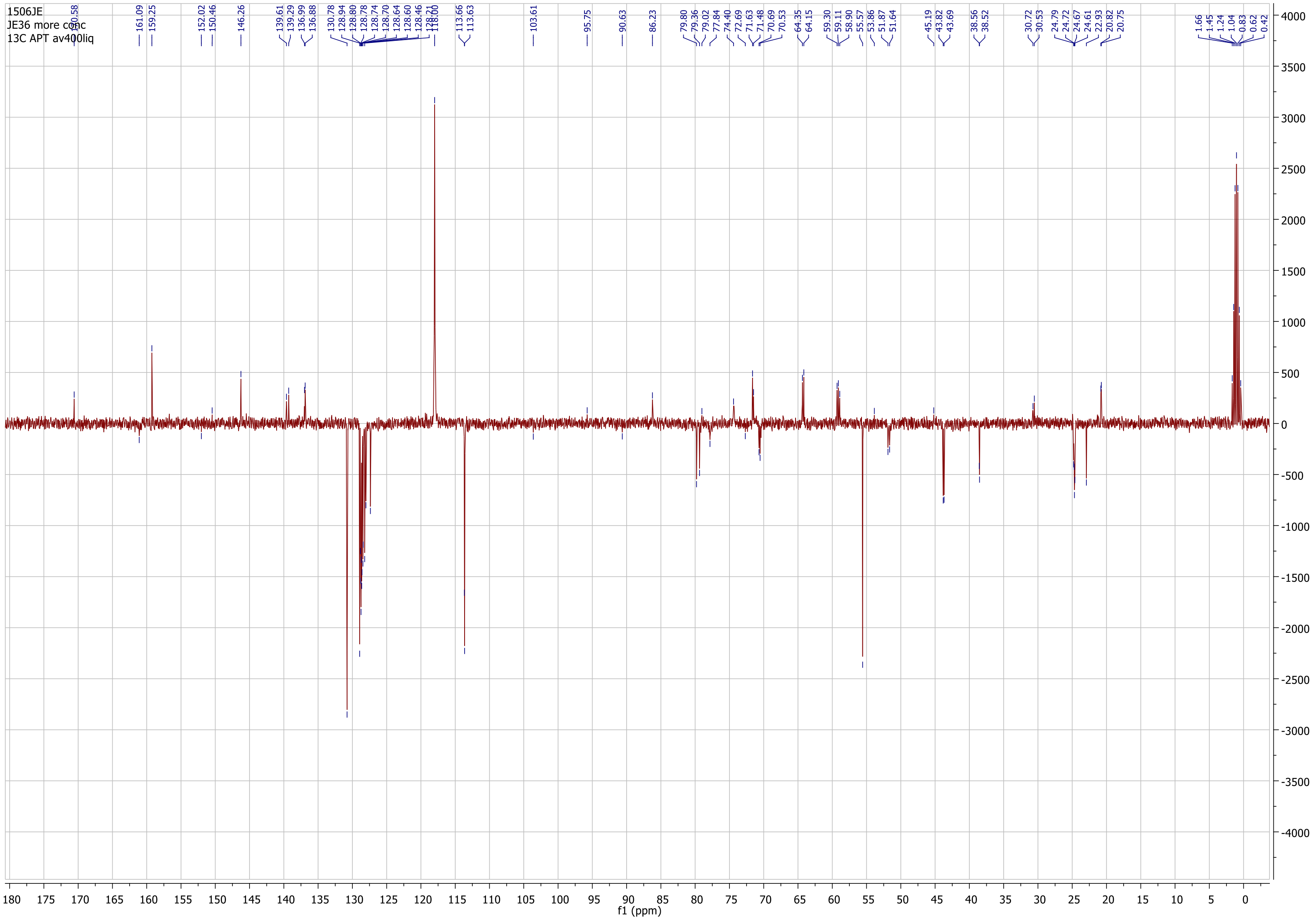

Supplementary Figure 11 1-O-((N,N-Diisopropylamino)-O-2-cyanoethyl-phosphoramidite))-2-Acetamido-3,4-di-O-benzyl-2-deoxy-6-O-(bis(4-methoxyphenyl)(phenyl))-5a-carba-α-D-mannopyranose **13**

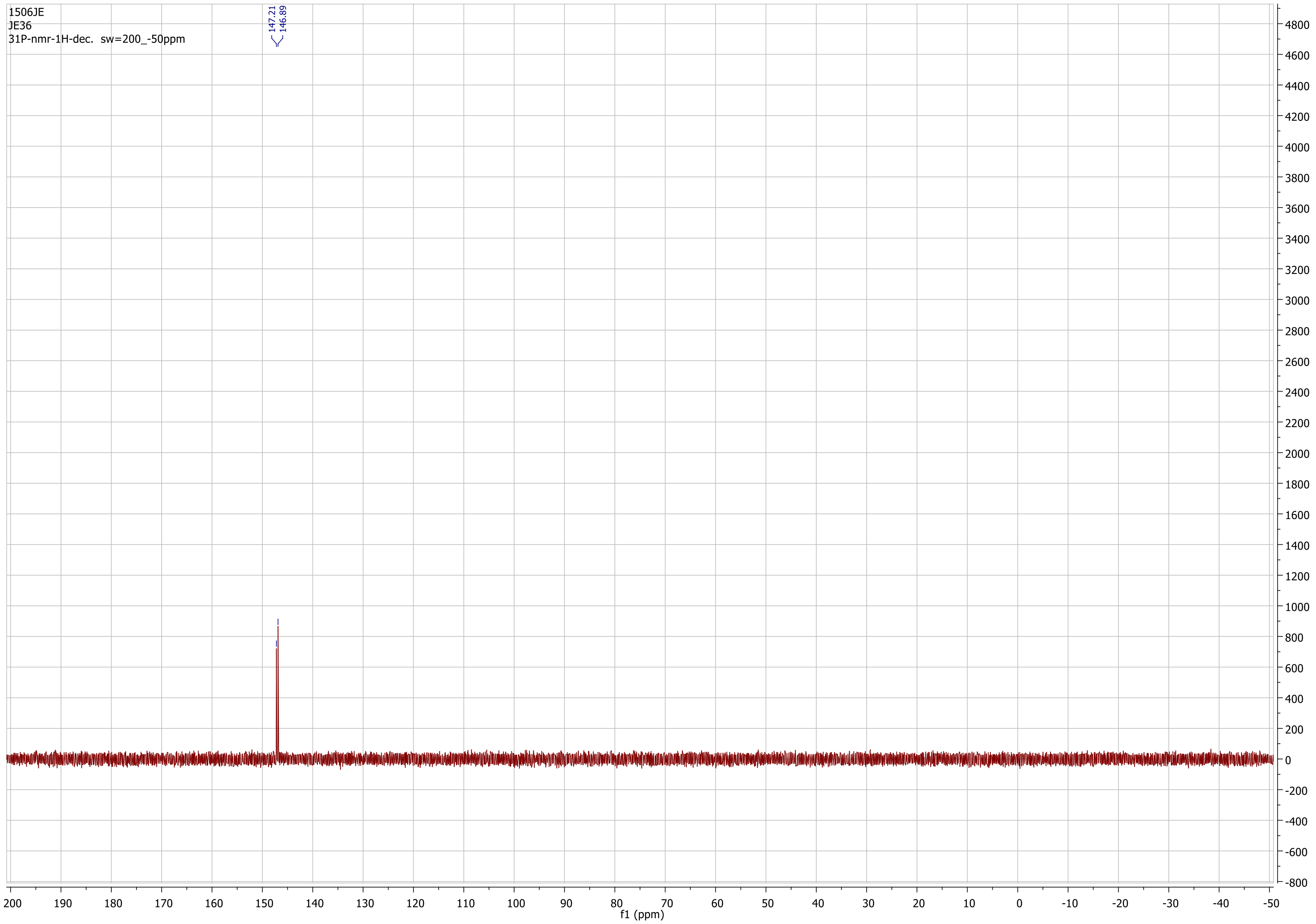

Supplementary Figure 12 1-O-((N,N-Diisopropylamino)-O-2-cyanoethyl-phosphoramidite))-2-Acetamido-3,4-di-O-benzyl-2-deoxy-6-O-(bis(4-methoxyphenyl)(phenyl))-5a-carba-α-D-mannopyranose **13**

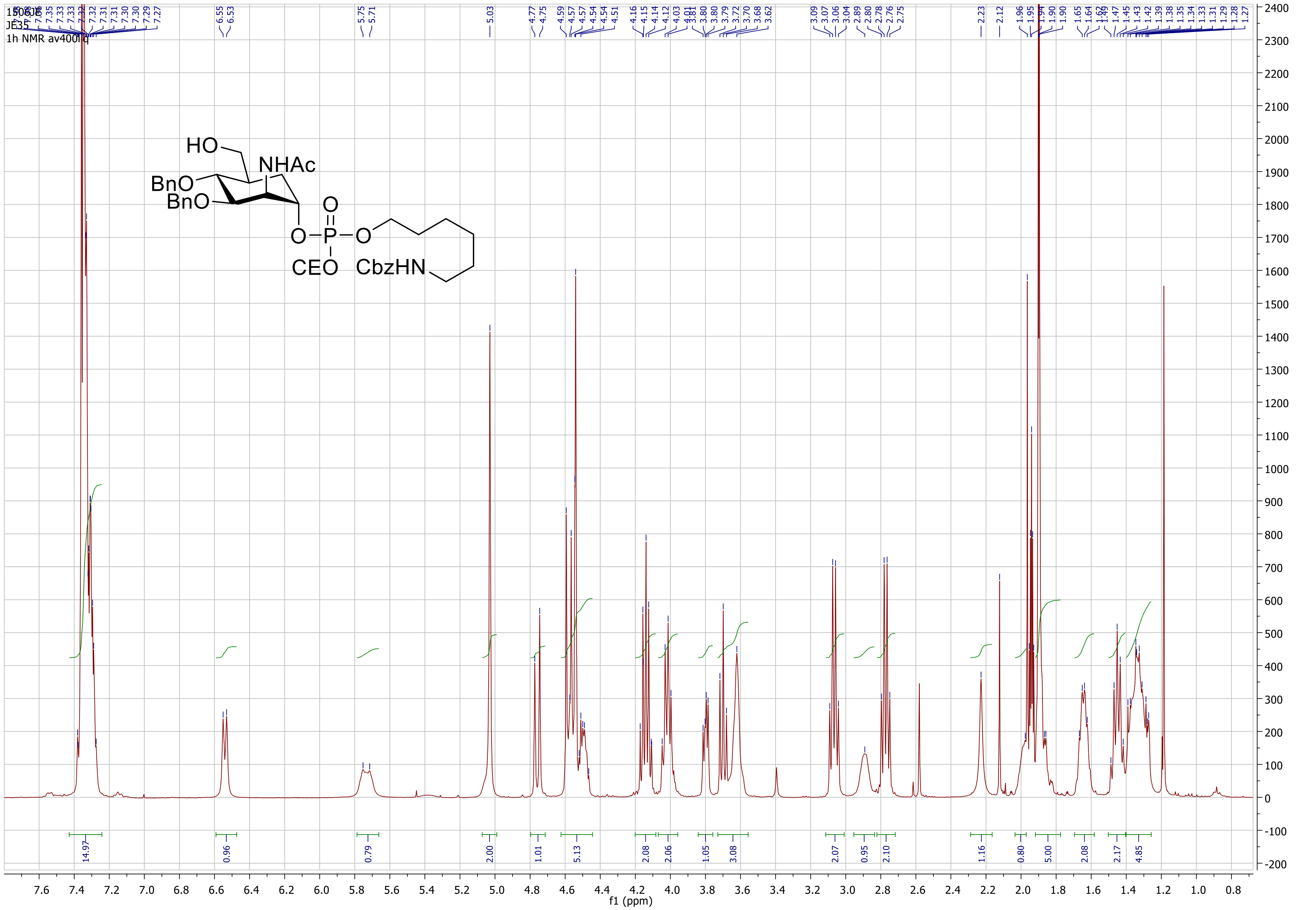

Supplementary Figure 13 1-O-((2-Acetamido-3,4-di-O-benzyl-2-deoxy-5a-carba-α-D-mannopyranosyl-1-O-phosphoryl)2-cyanoethyl)-6-hexyl-benzyl-carbamate **15**

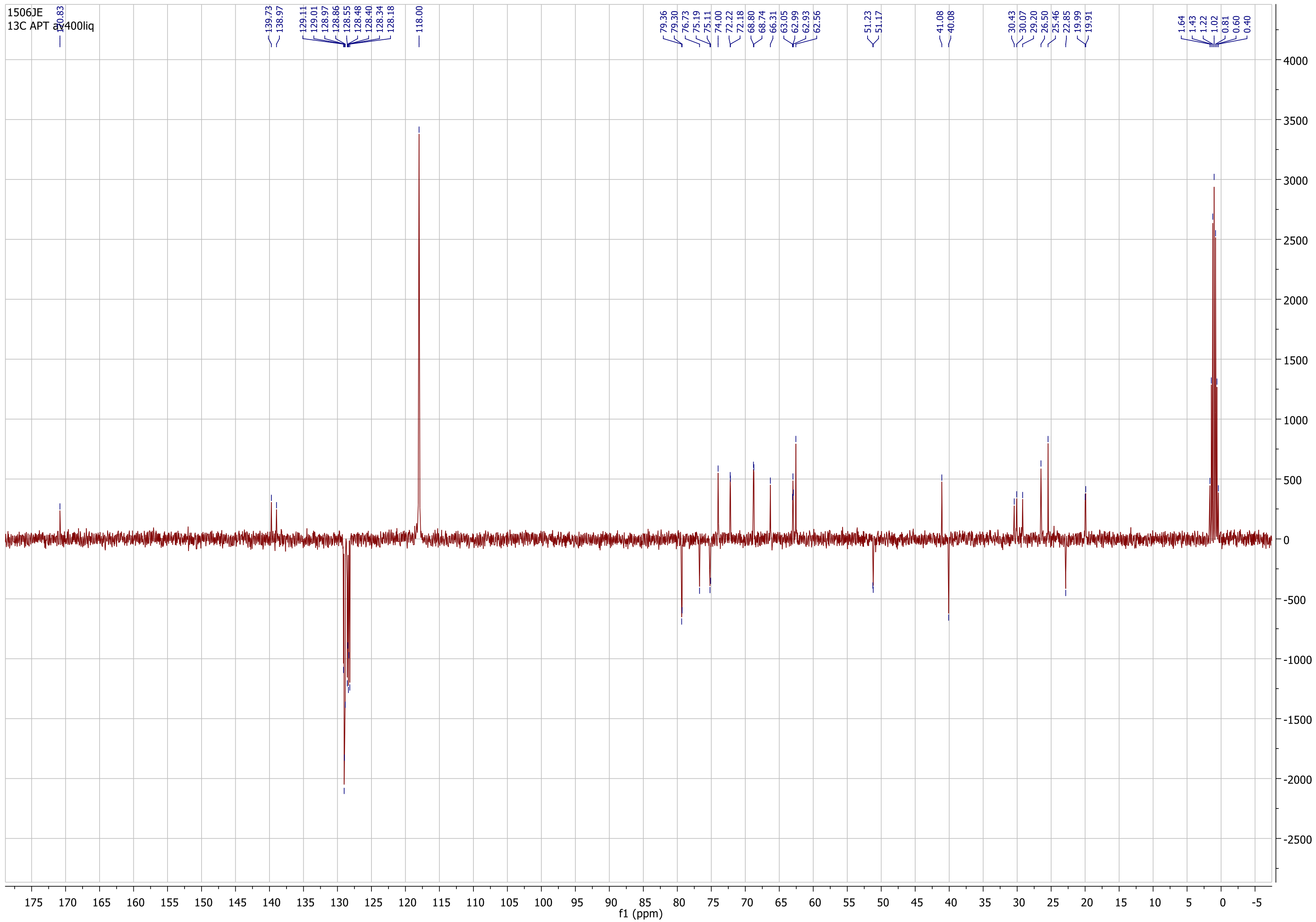

Supplementary Figure 14 1-O-((2-Acetamido-3,4-di-O-benzyl-2-deoxy-5a-carba-α-D-mannopyranosyl-1-O-phosphoryl)2-cyanoethyl)-6-hexyl-benzyl-carbamate **15**

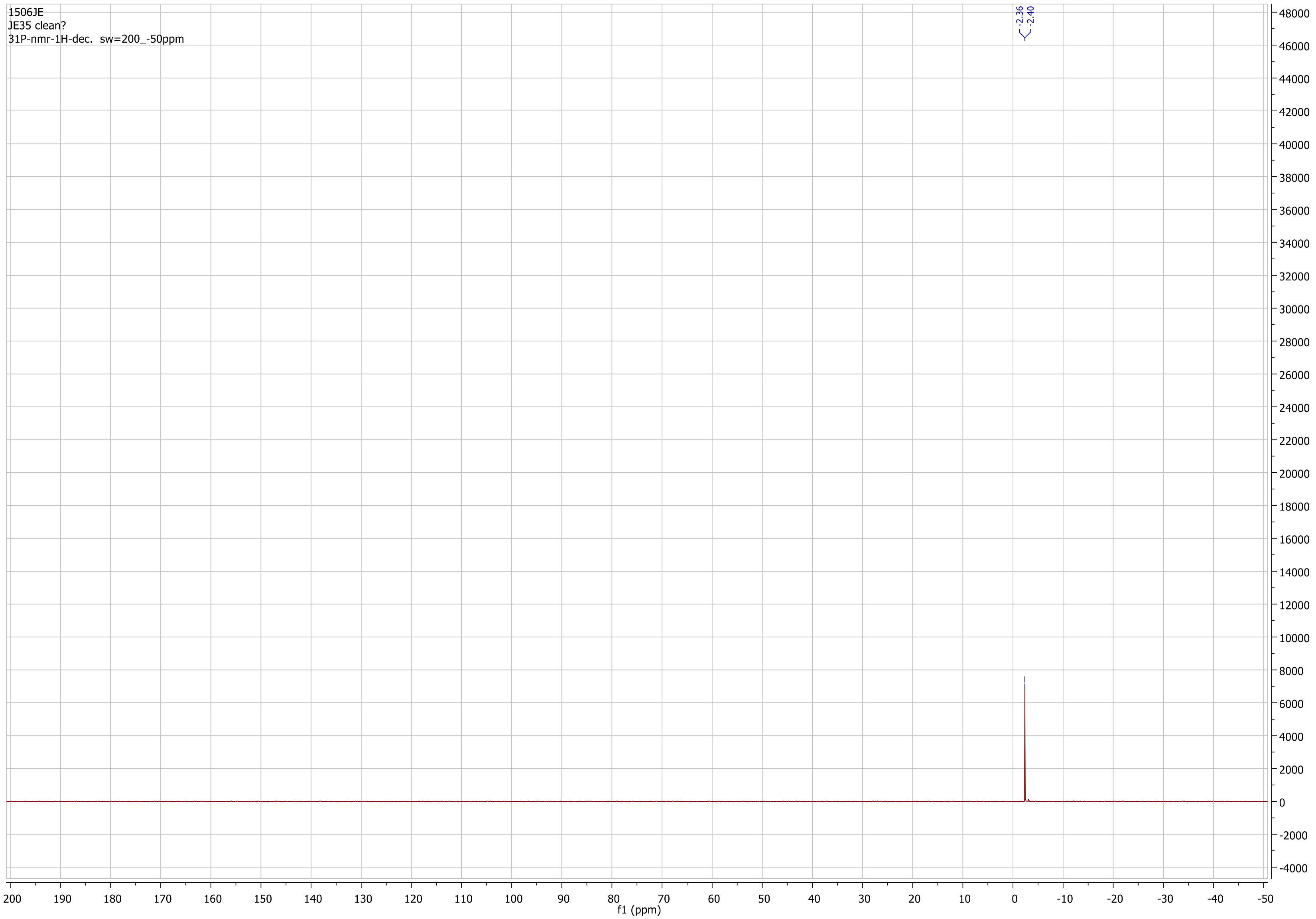

Supplementary Figure 15 1-O-((2-Acetamido-3,4-di-O-benzyl-2-deoxy-5a-carba-α-D-mannopyranosyl-1-O-phosphoryl)2-cyanoethyl)-6-hexyl-benzyl-carbamate **15**

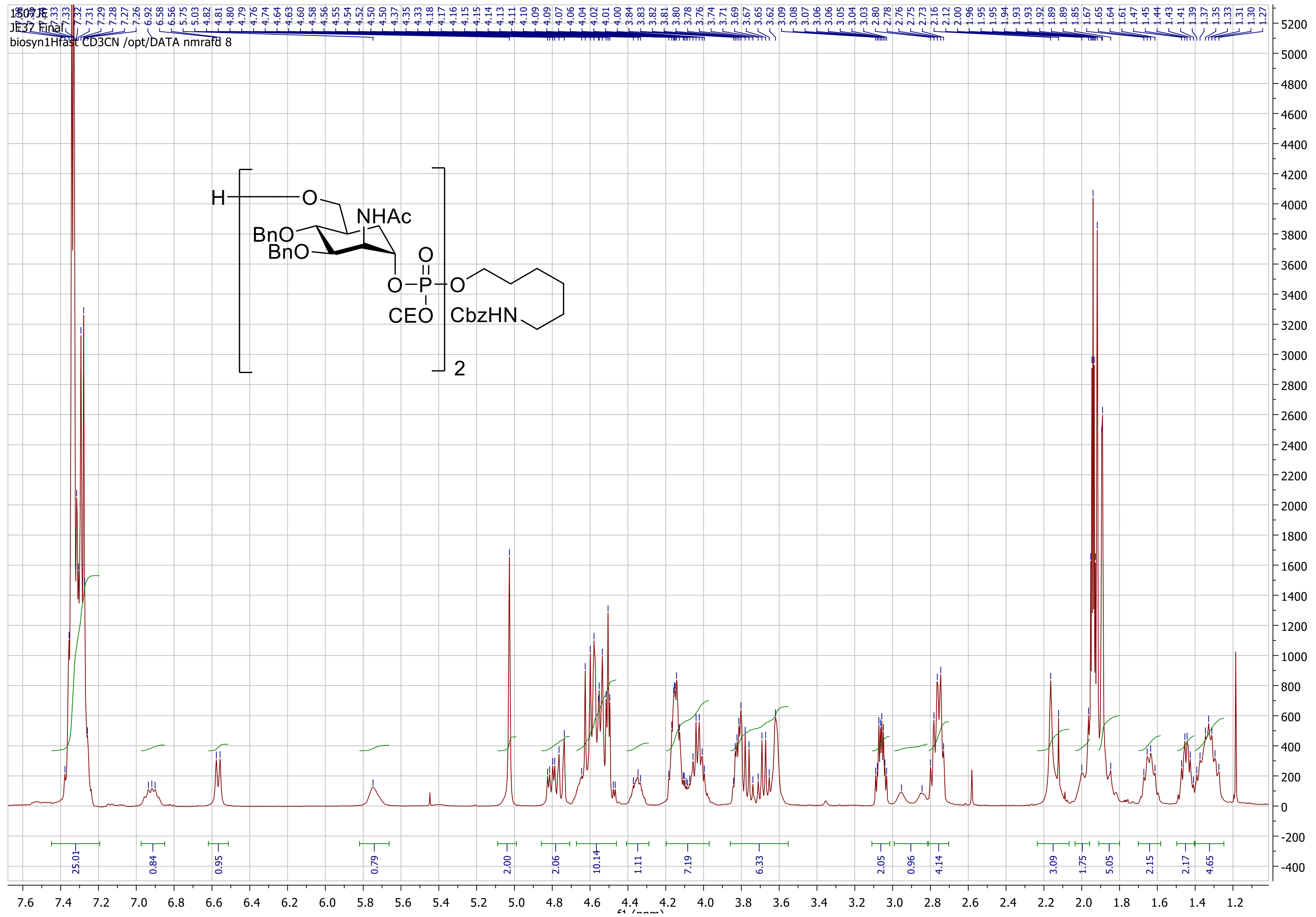

Supplementary Figure 16 1-O-di-((2-Acetamido-3,4-di-O-benzyl-2-deoxy-5a-carba-α-D-mannopyranosyl-1-O-phosphoryl)2-cyanoethyl)-6-hexyl-benzylcarbamate **16**

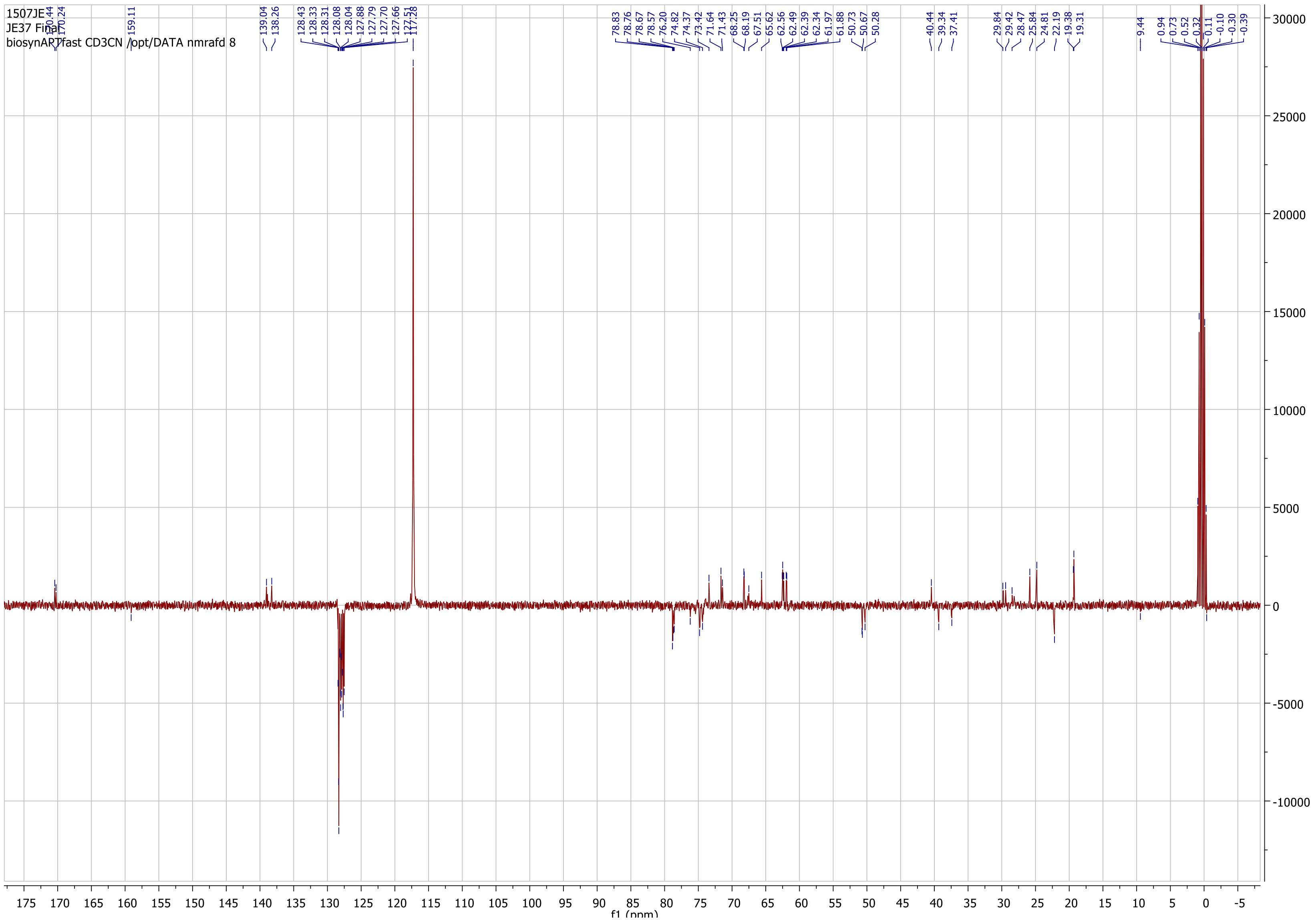

Supplementary Figure 17 1-O-di-((2-Acetamido-3,4-di-O-benzyl-2-deoxy-5a-carba-α-D-mannopyranosyl-1-O-phosphoryl)2-cyanoethyl)-6-hexyl-benzylcarbamate **16**

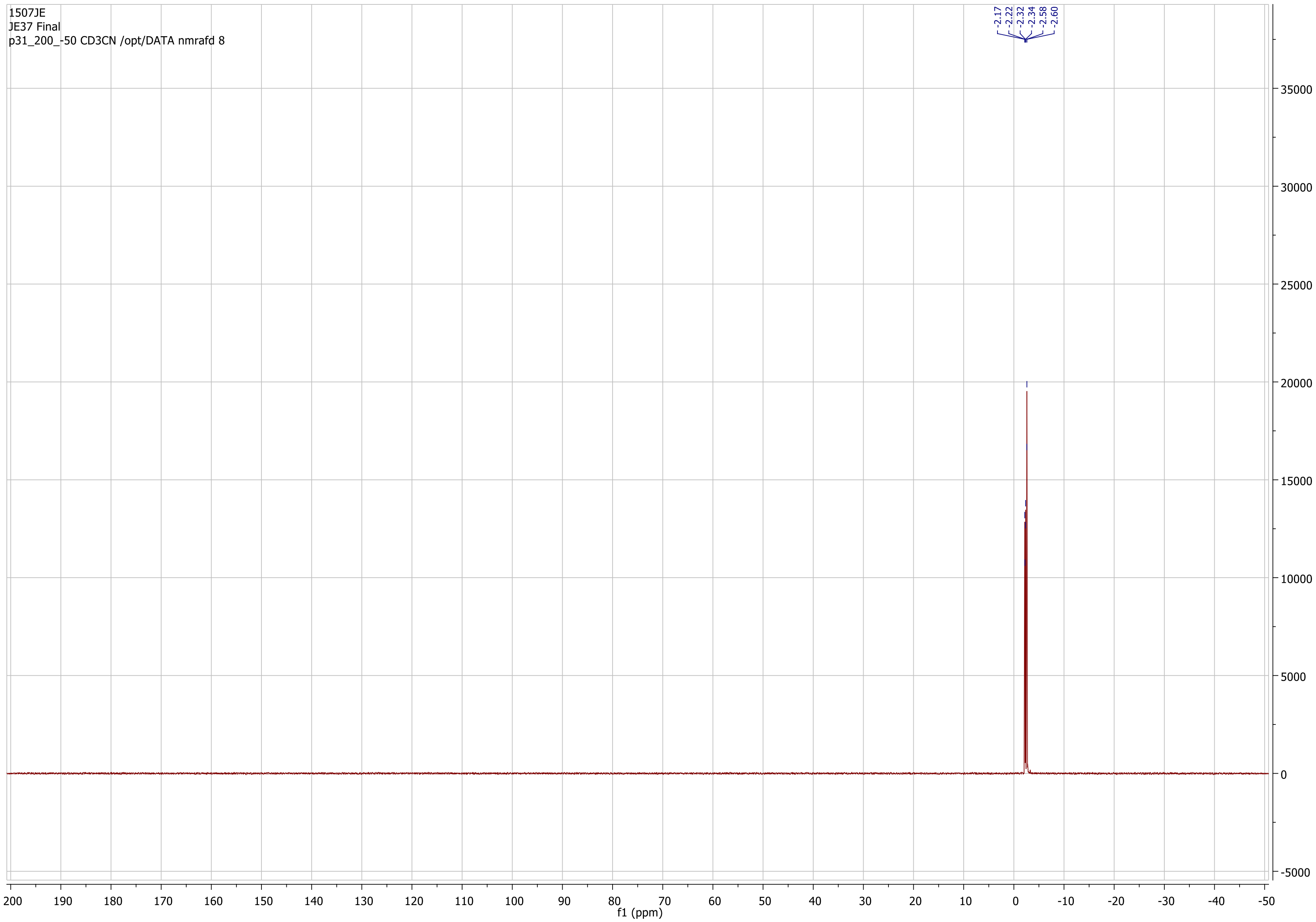

Supplementary Figure 18 1-O-di-((2-Acetamido-3,4-di-O-benzyl-2-deoxy-5a-carba-α-D-mannopyranosyl-1-O-phosphoryl)2-cyanoethyl)-6-hexyl-benzylcarbamate **16**

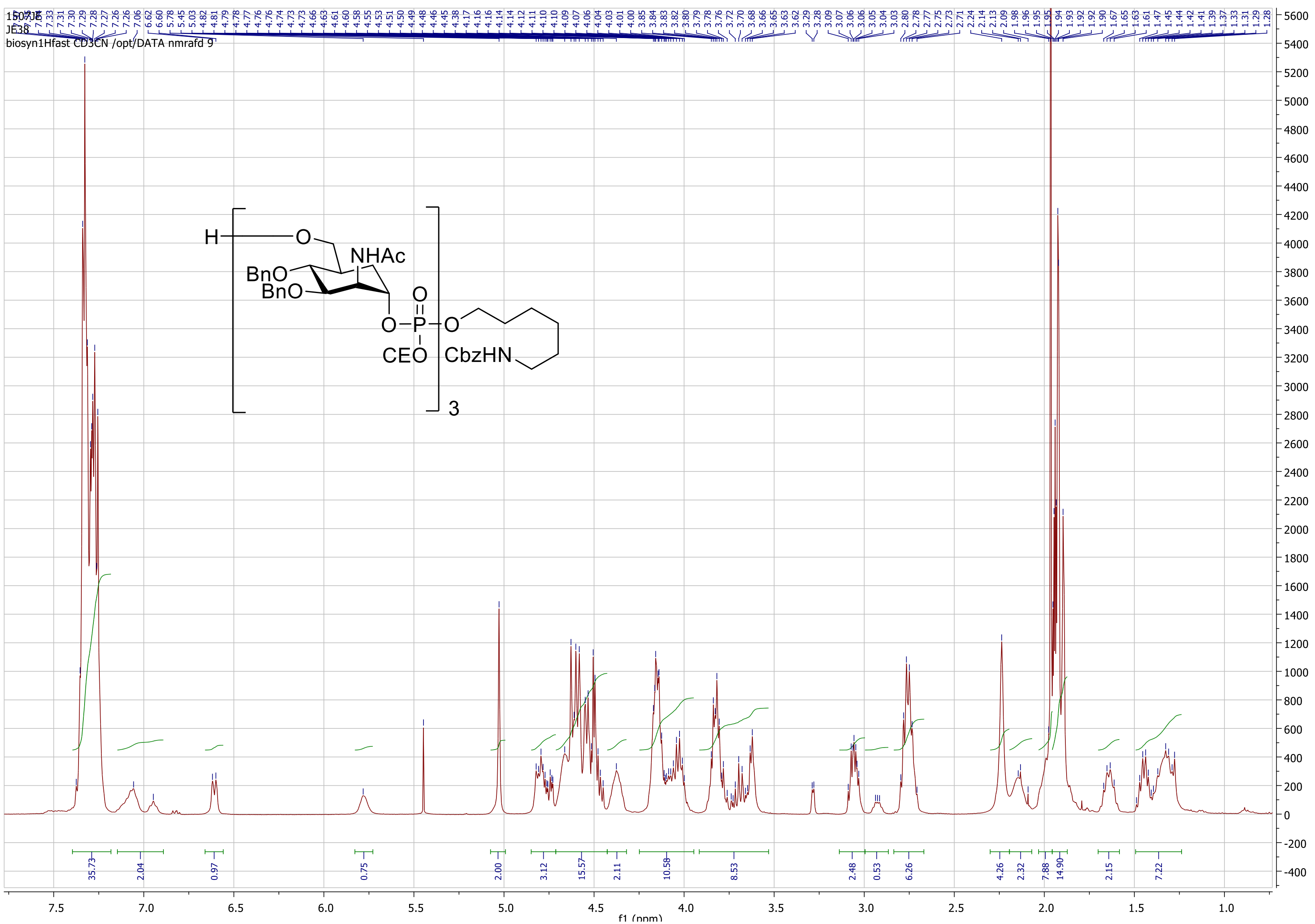

Supplementary Figure 19 1-O-tri-((2-Acetamido-3,4-di-O-benzyl-2-deoxy-5a-carba-α-D-mannopyranosyl-1-O-phosphoryl)2-cyanoethyl)-6-hexyl-benzylcarbamate **17**

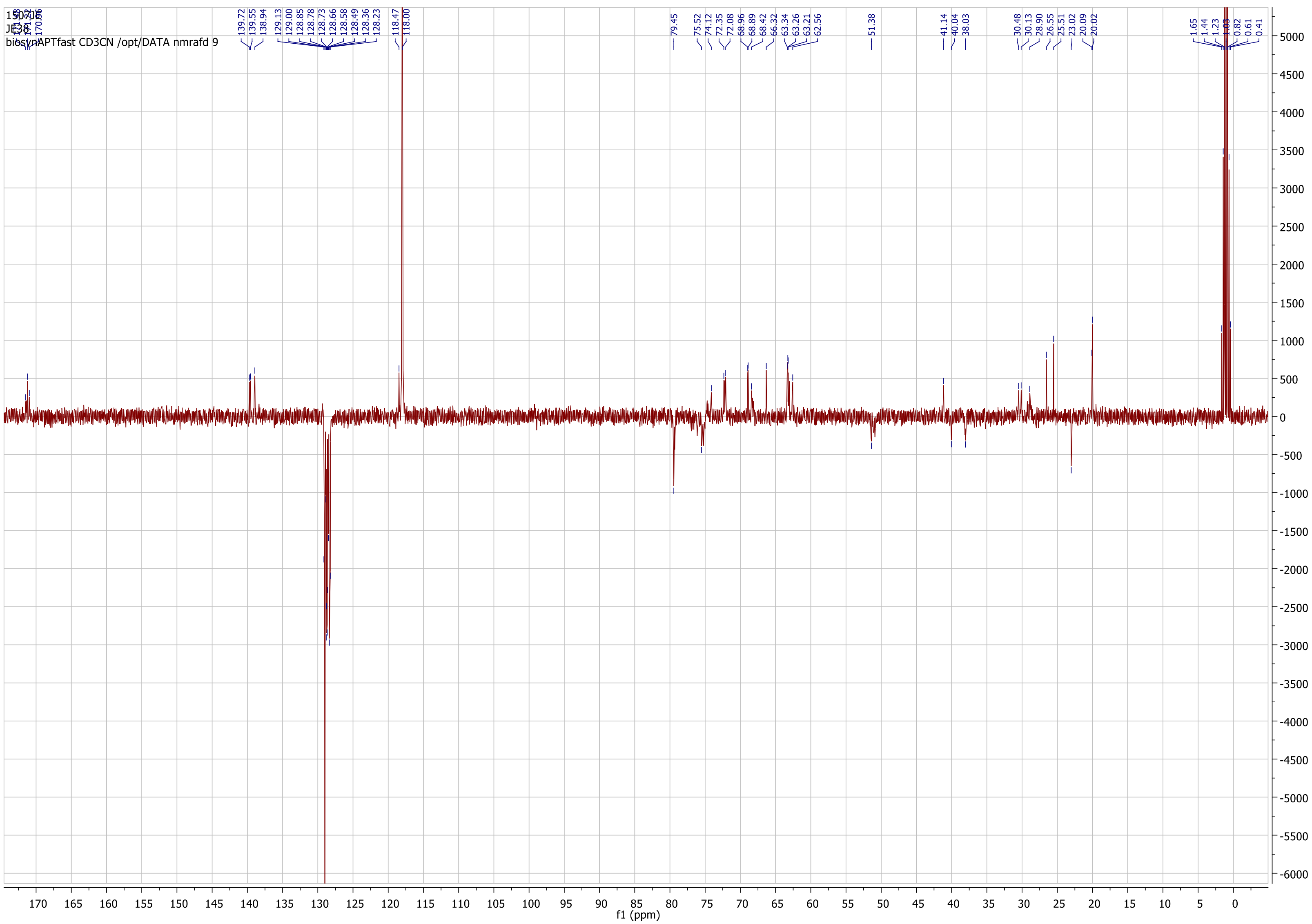

Supplementary Figure 20 1-O-tri-((2-Acetamido-3,4-di-O-benzyl-2-deoxy-5a-carba-α-D-mannopyranosyl-1-O-phosphoryl)2-cyanoethyl)-6-hexyl-benzylcarbamate **17**

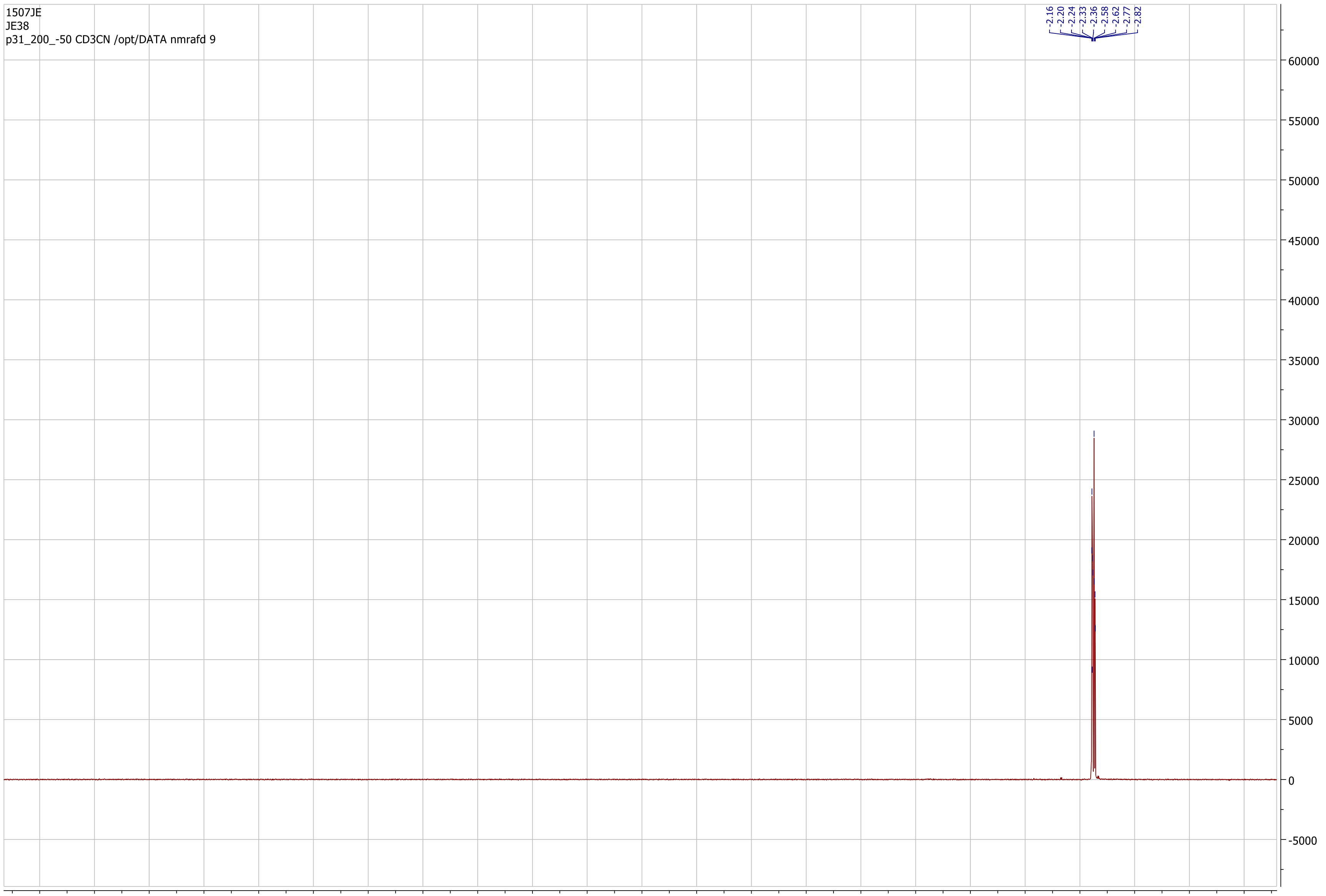

Supplementary Figure 21 1-O-tri-((2-Acetamido-3,4-di-O-benzyl-2-deoxy-5a-carba-α-D-mannopyranosyl-1-O-phosphoryl)2-cyanoethyl)-6-hexyl-benzylcarbamate**17**

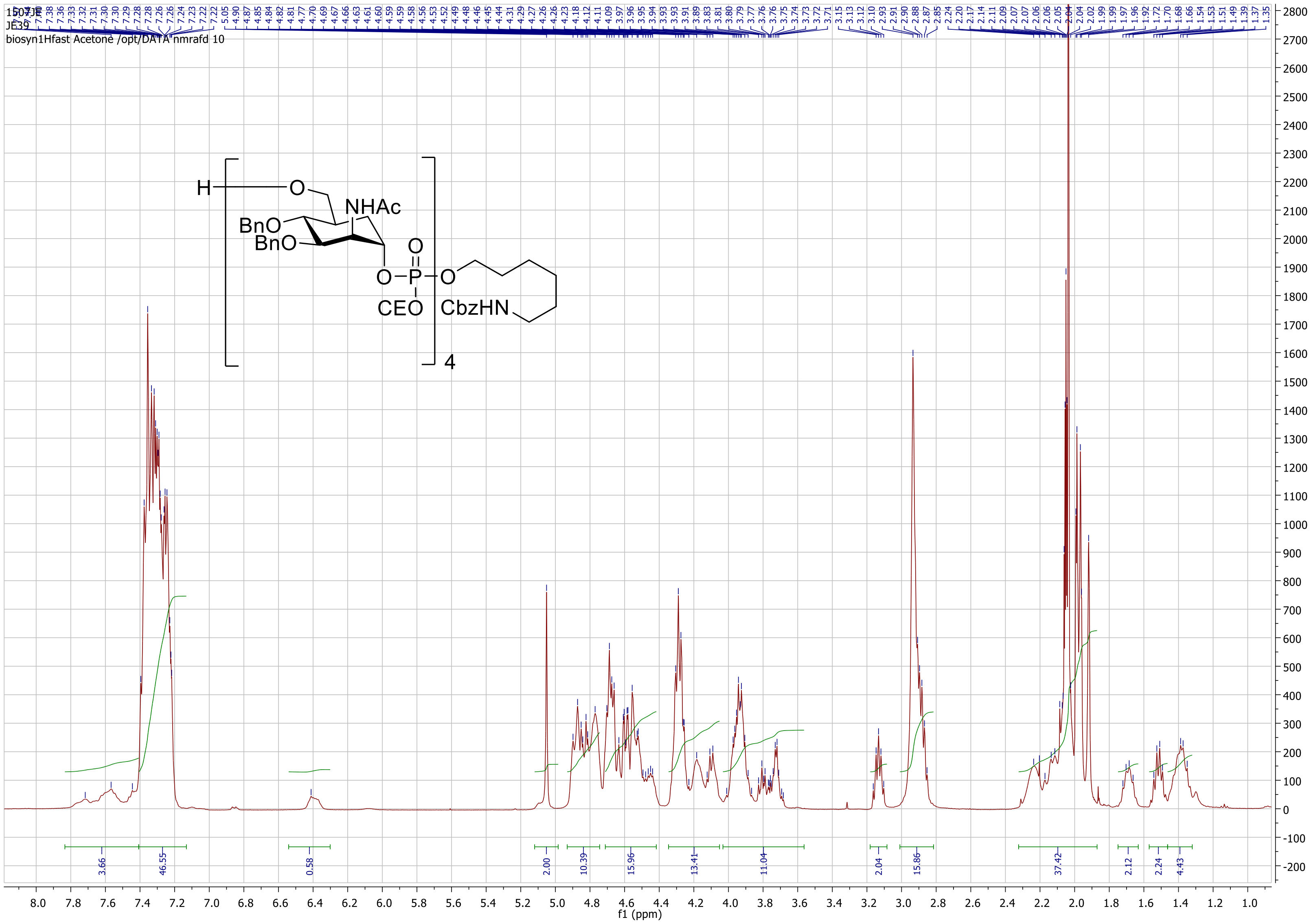

Supplementary Figure 22 1-O-tetra-((2-Acetamido-3,4-di-O-benzyl-2-deoxy-5a-carba-α-D-mannopyranosyl-1-O-phosphoryl)2-cyanoethyl)-6-hexyl-benzyl-carbamate **18**

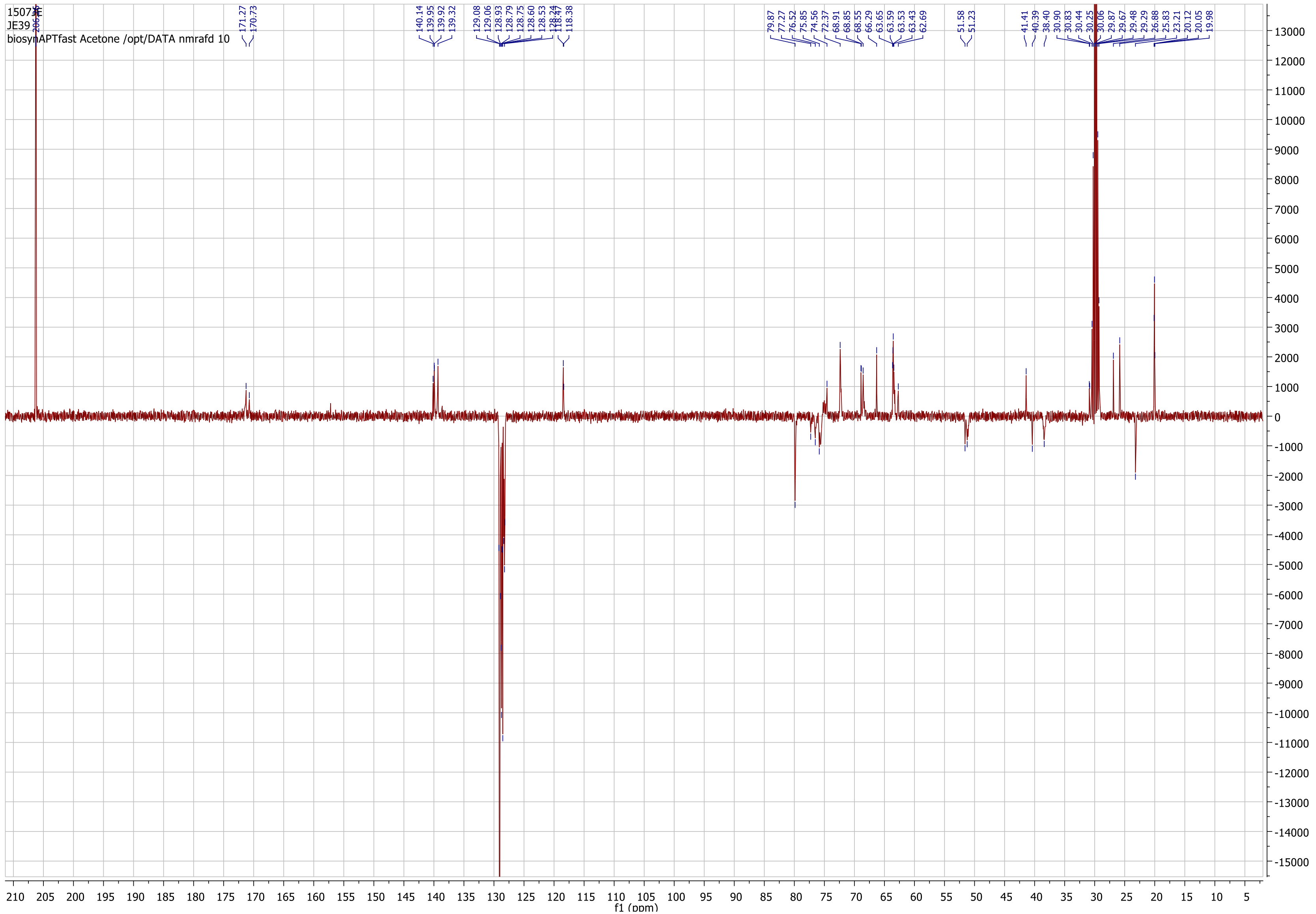

Supplementary Figure 23 1-O-tetra-((2-Acetamido-3,4-di-O-benzyl-2-deoxy-5a-carba-α-D-mannopyranosyl-1-O-phosphoryl)2-cyanoethyl)-6-hexyl-benzyl-carbamate **18**

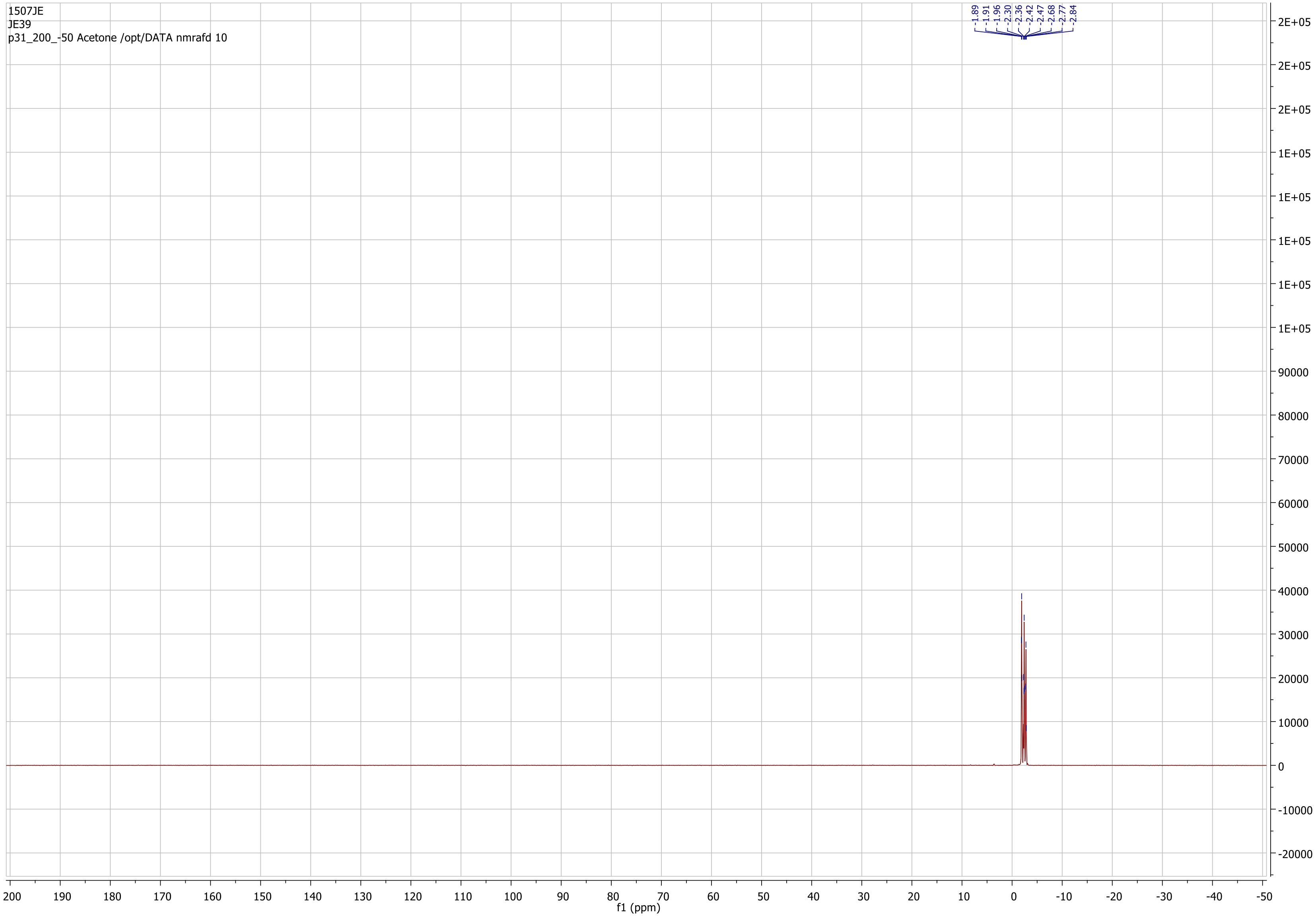

Supplementary Figure 24 1-O-tetra-((2-Acetamido-3,4-di-O-benzyl-2-deoxy-5a-carba-α-D-mannopyranosyl-1-O-phosphoryl)2-cyanoethyl)-6-hexyl-benzyl-carbamate **18**

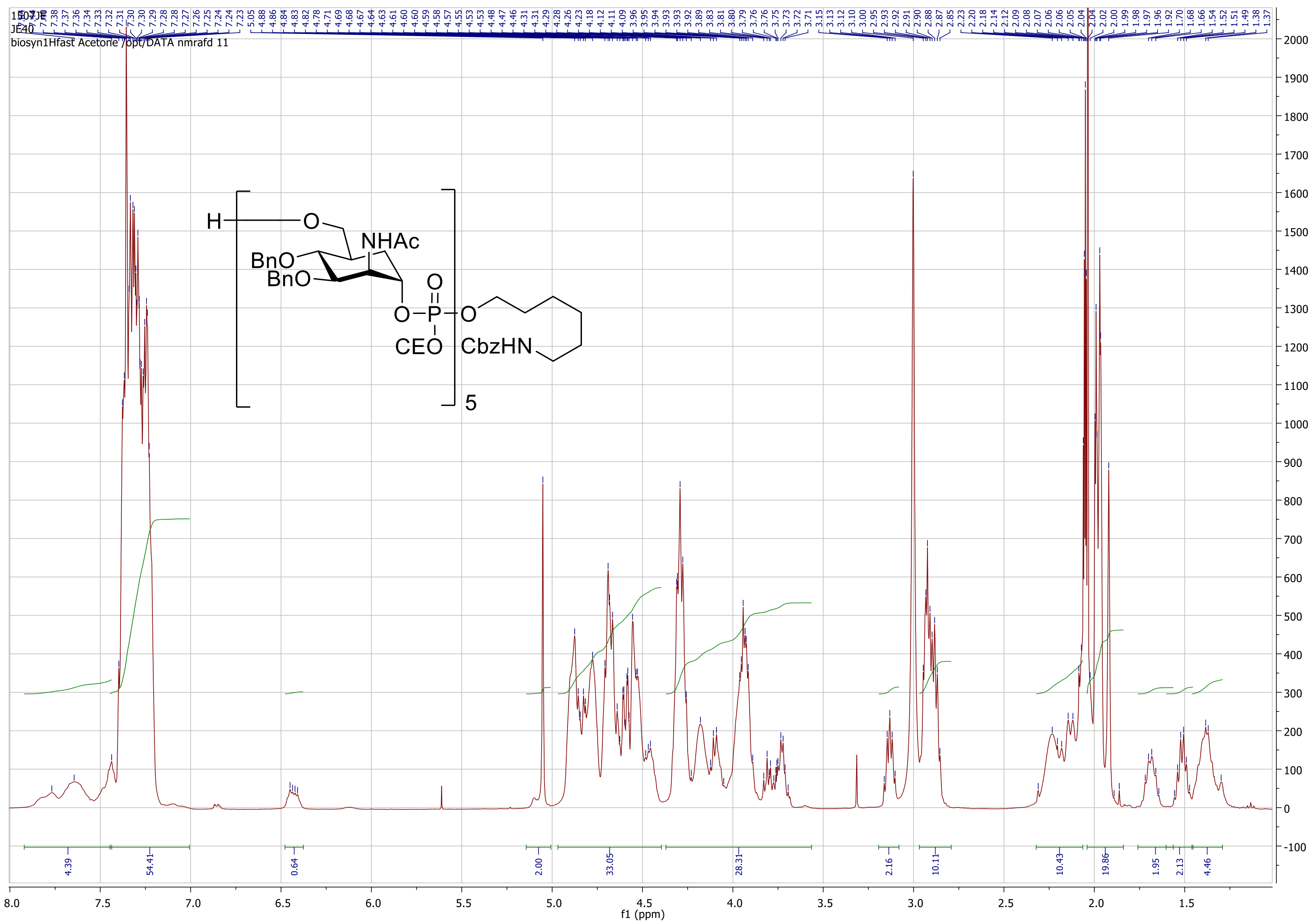

Supplementary Figure 25 1-O-penta-((2-Acetamido-3,4-di-O-benzyl-2-deoxy-5a-carba-α-D-mannopyranosyl-1-O-phosphoryl)2-cyanoethyl)-6-hexyl-benzyl-carbamate **19**

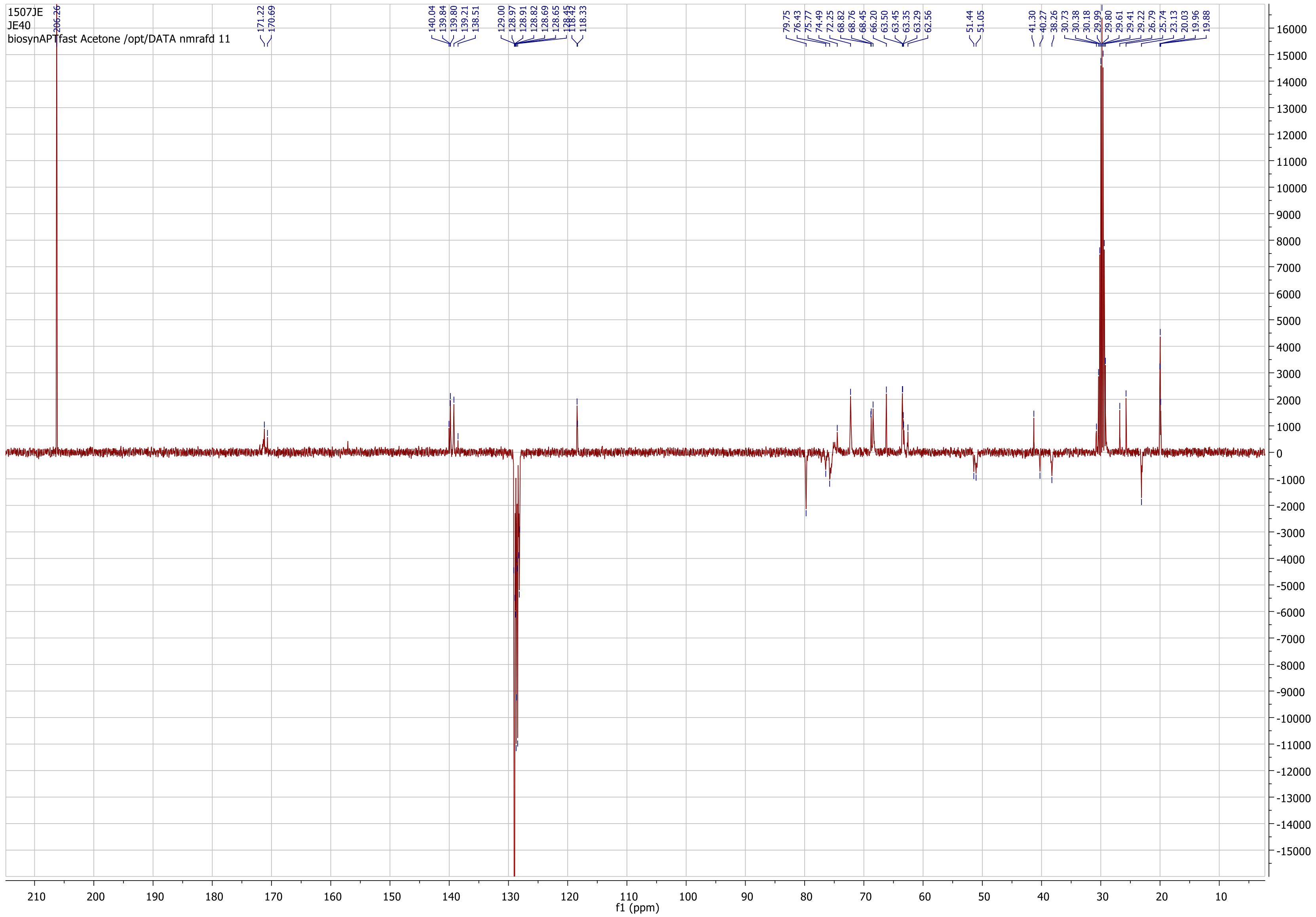

Supplementary Figure 26 1-O-penta-((2-Acetamido-3,4-di-O-benzyl-2-deoxy-5a-carba-α-D-mannopyranosyl-1-O-phosphoryl)2-cyanoethyl)-6-hexyl-benzyl-carbamate 19

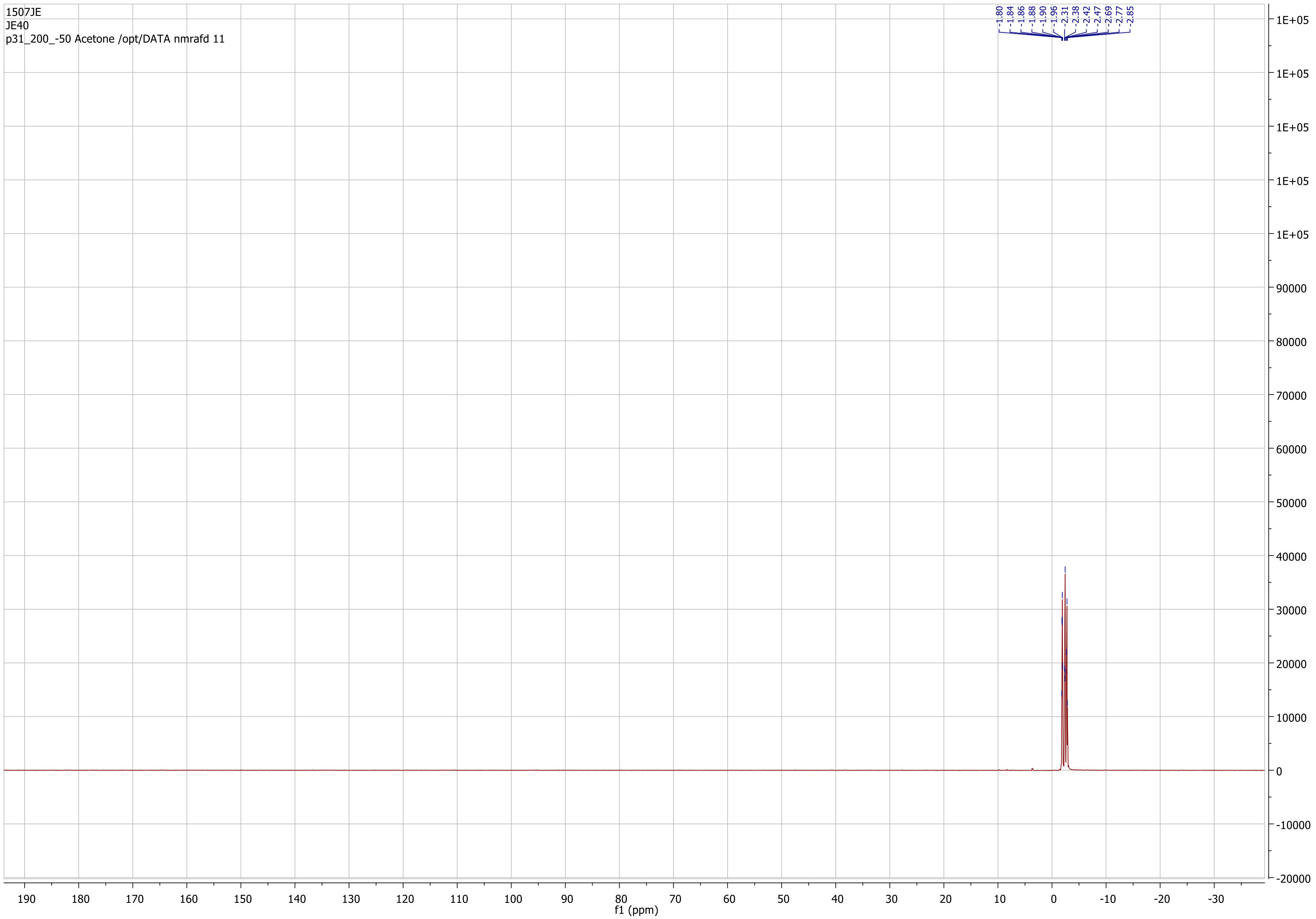

Supplementary Figure 27 1-O-penta-((2-Acetamido-3,4-di-O-benzyl-2-deoxy-5a-carba-α-D-mannopyranosyl-1-O-phosphoryl)2-cyanoethyl)-6-hexyl-benzyl-carbamate **19**

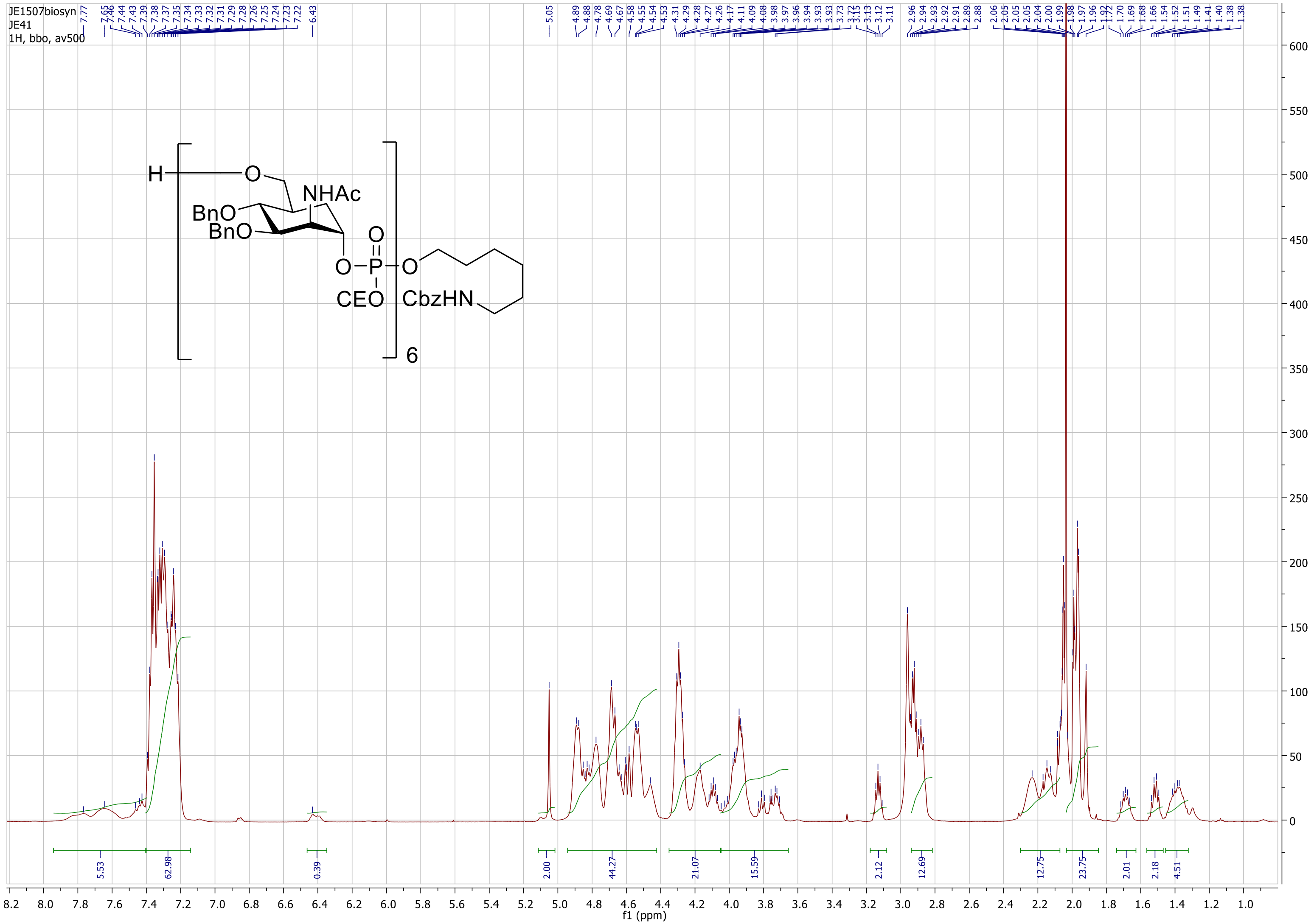

Supplementary Figure 28 1-O-hexa-((2-Acetamido-3,4-di-O-benzyl-2-deoxy-5a-carba-α-D-mannopyranosyl-1-O-phosphoryl)2-cyanoethyl)-6-hexyl-benzyl-carbamate **20**

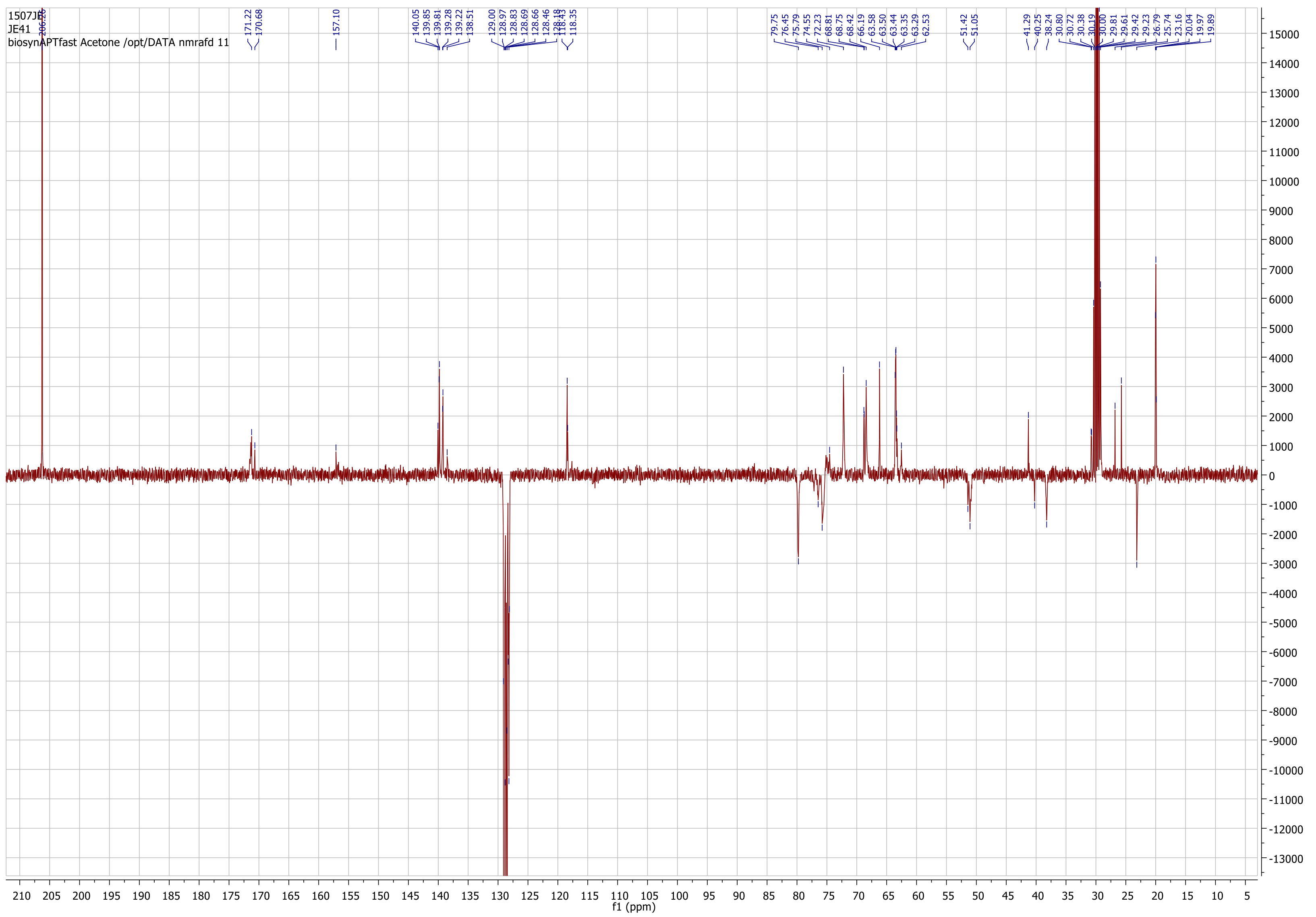

Supplementary Figure 29 1-O-hexa-((2-Acetamido-3,4-di-O-benzyl-2-deoxy-5a-carba-α-D-mannopyranosyl-1-O-phosphoryl)2-cyanoethyl)-6-hexyl-benzyl-carbamate **20**

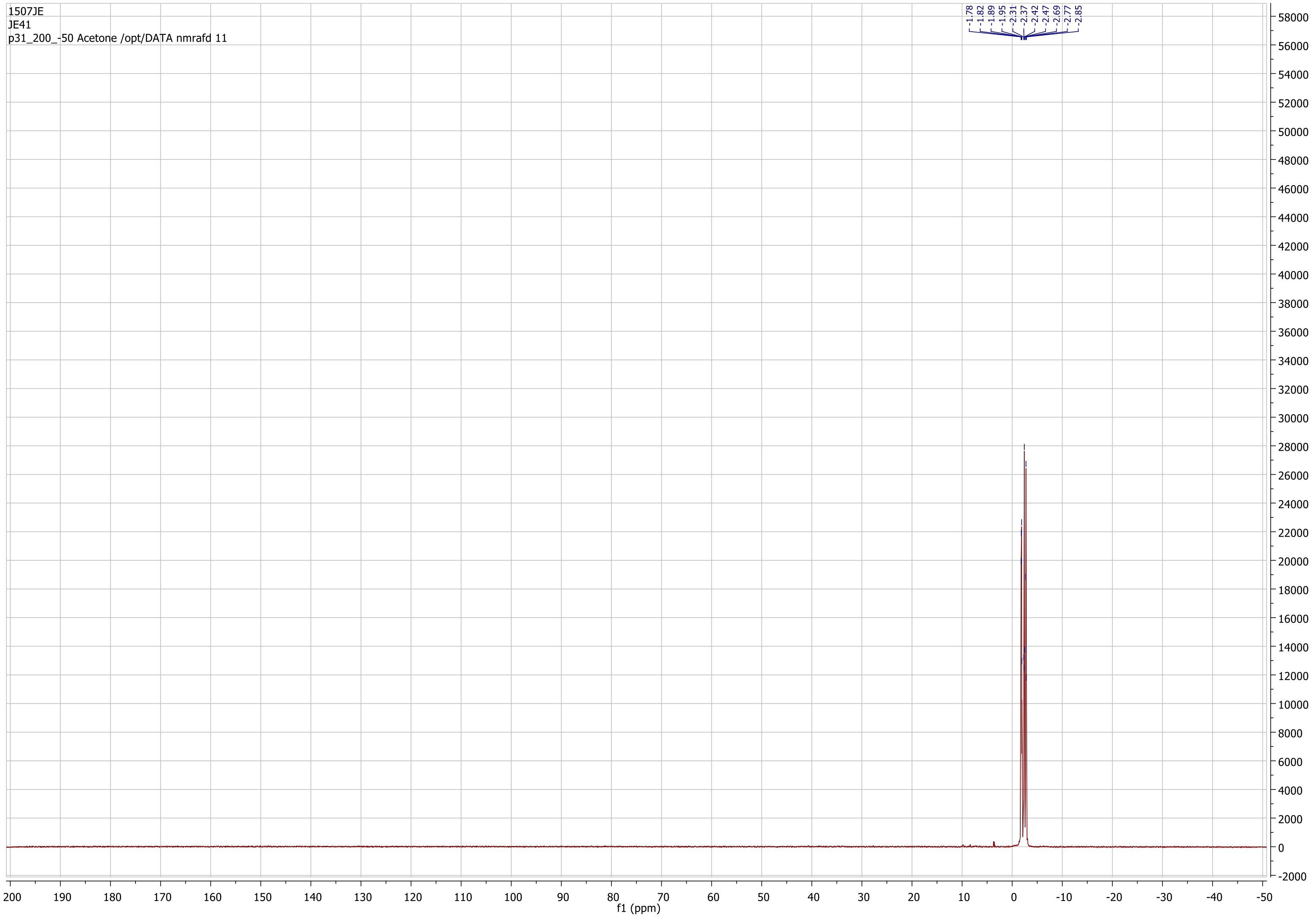

Supplementary Figure 30 1-O-hexa-((2-Acetamido-3,4-di-O-benzyl-2-deoxy-5a-carba-α-D-mannopyranosyl-1-O-phosphoryl)2-cyanoethyl)-6-hexyl-benzyl-carbamate **20**

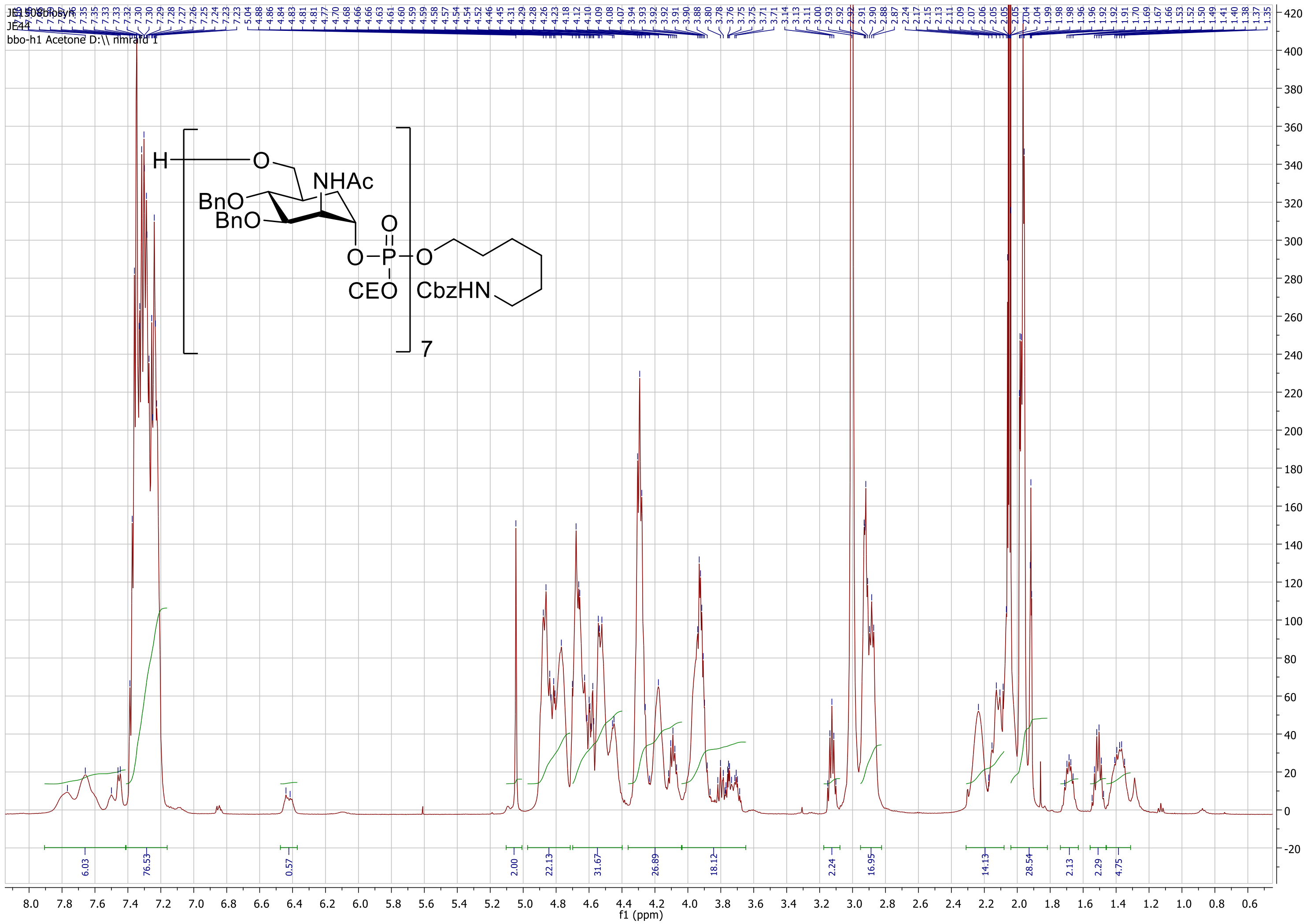

Supplementary Figure 31 1-O-epta-((2-Acetamido-3,4-di-O-benzyl-2-deoxy-5a-carba-α-D-mannopyranosyl-1-O-phosphoryl)2-cyanoethyl)-6-hexyl-benzyl-carbamate **21**

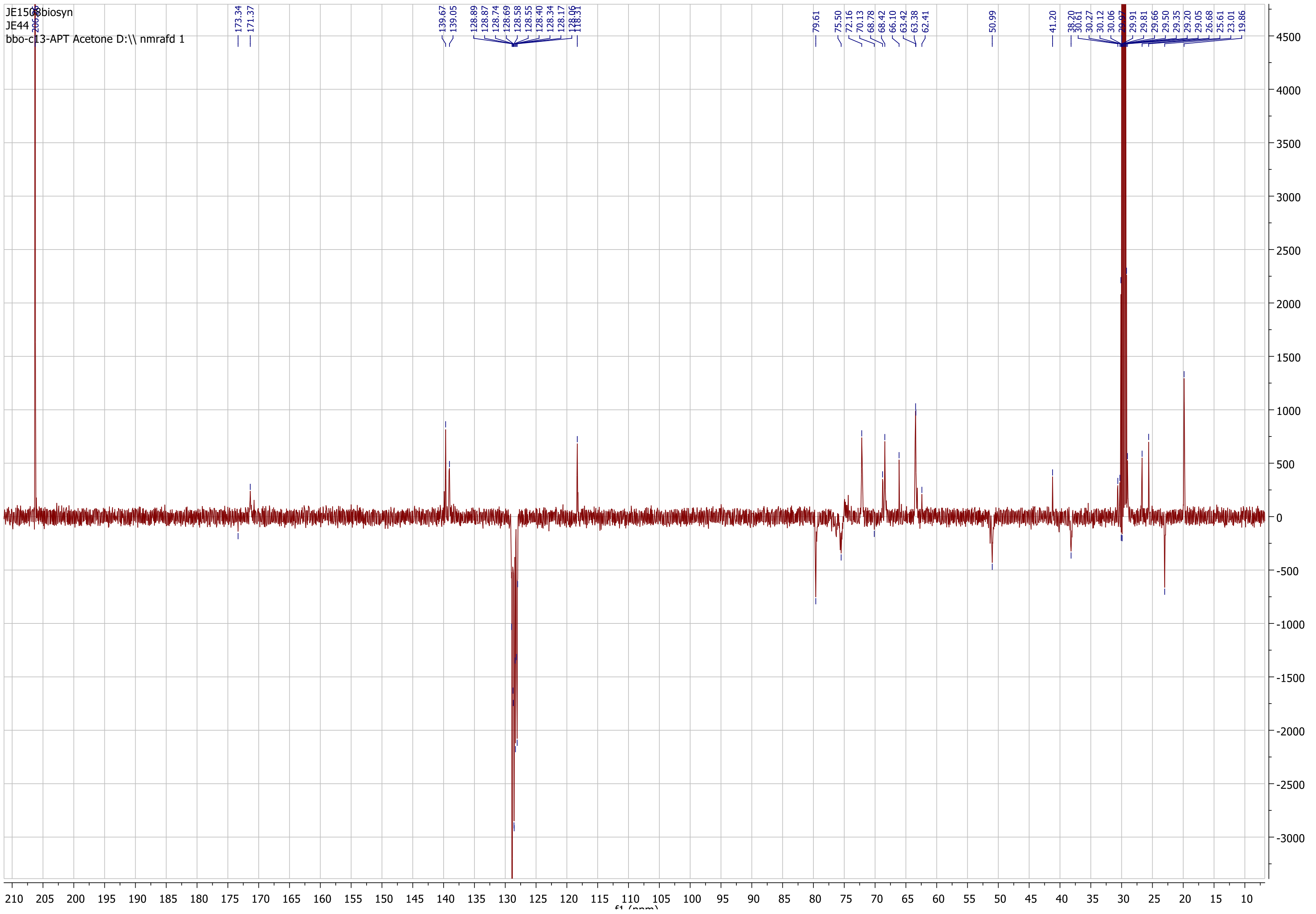

Supplementary Figure 32 1-O-epta-((2-Acetamido-3,4-di-O-benzyl-2-deoxy-5a-carba-α-D-mannopyranosyl-1-O-phosphoryl)2-cyanoethyl)-6-hexyl-benzyl-carbamate **21**

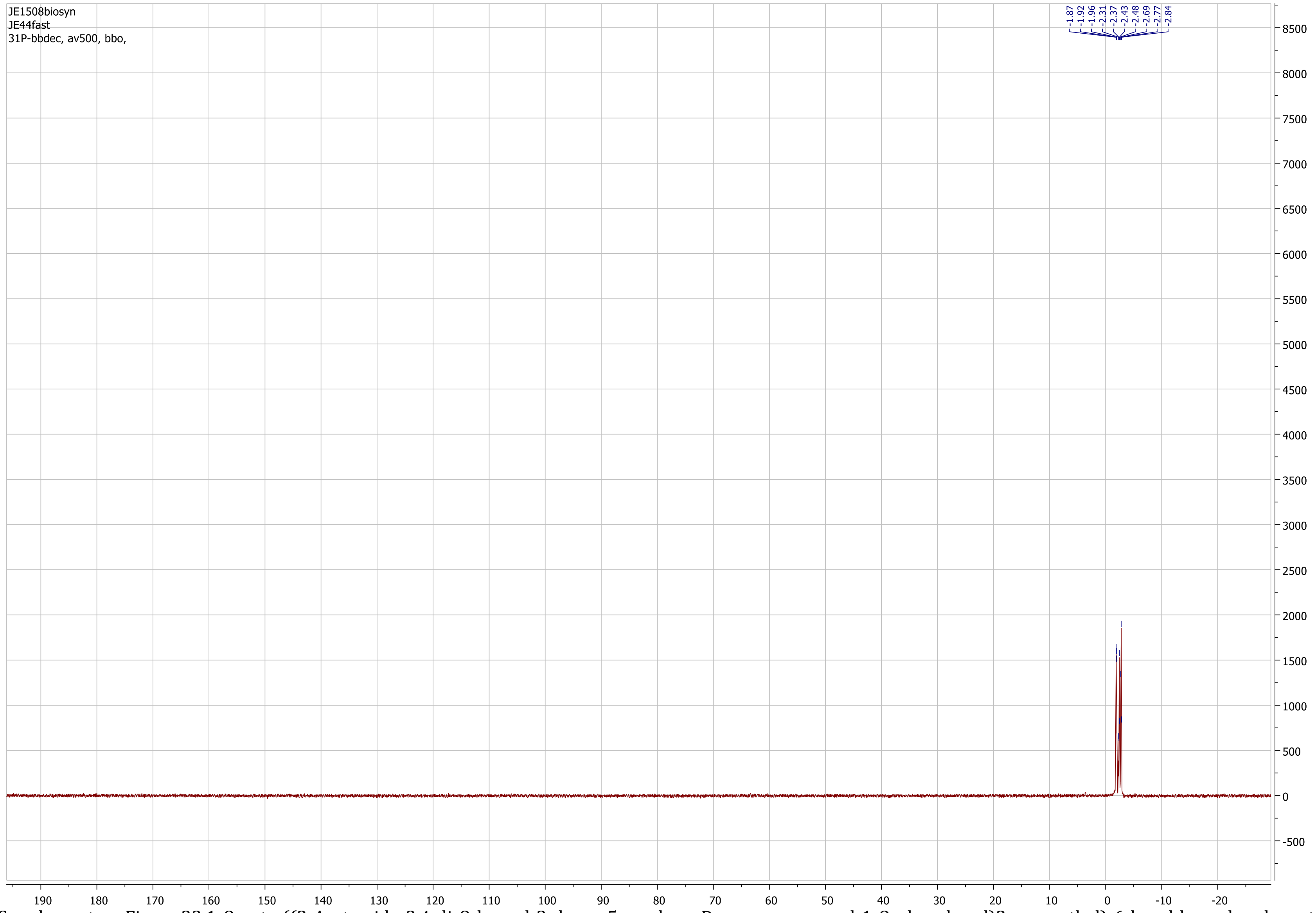

Supplementary Figure 33 1-O-epta-((2-Acetamido-3,4-di-O-benzyl-2-deoxy-5a-carba-α-D-mannopyranosyl-1-O-phosphoryl)2-cyanoethyl)-6-hexyl-benzyl-carbamate **21**

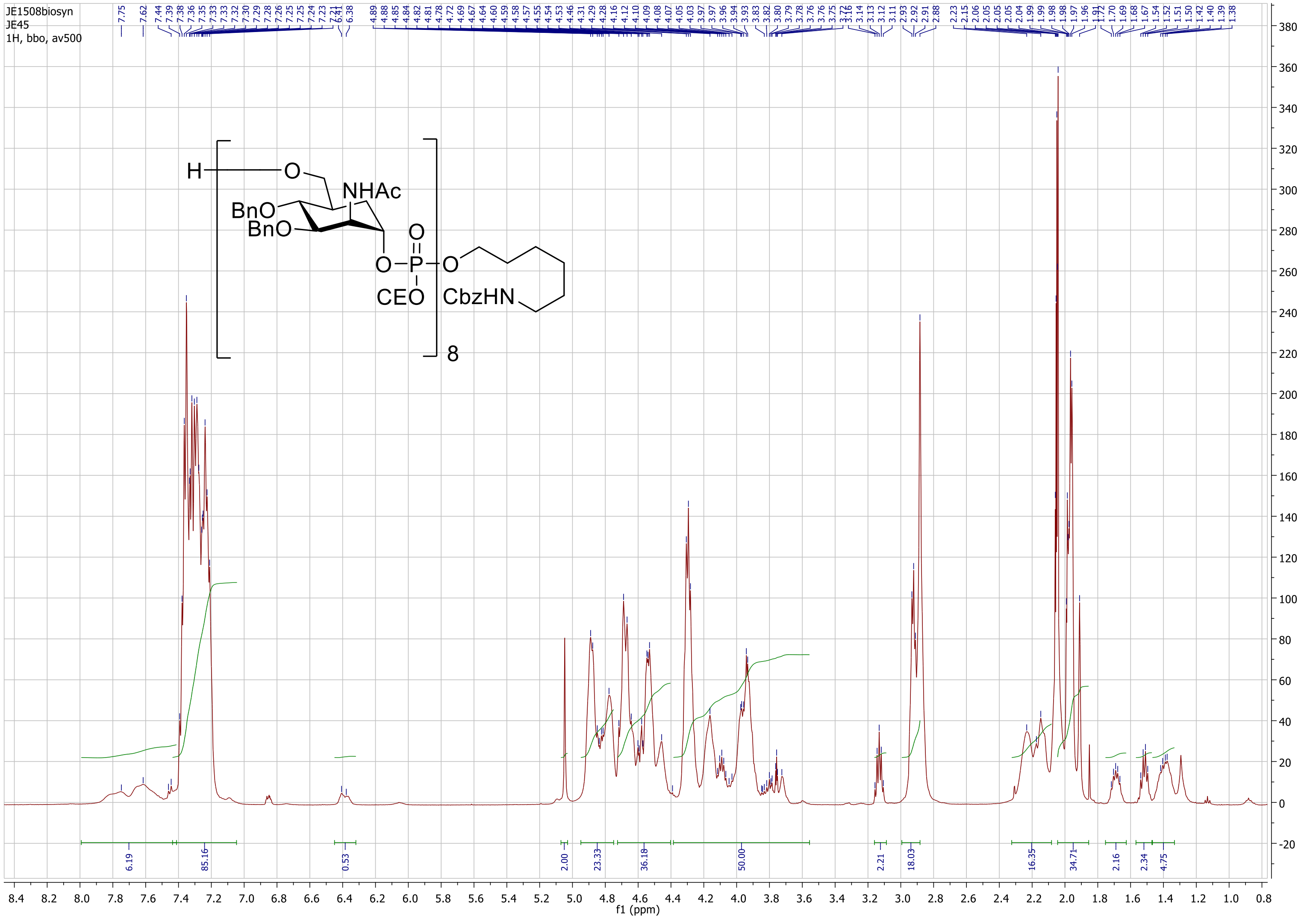

Supplementary Figure 34 1-O-octa-((2-Acetamido-3,4-di-O-benzyl-2-deoxy-5a-carba-α-D-mannopyranosyl-1-O-phosphoryl)2-cyanoethyl)-6-hexyl-benzyl-carbamate **22**

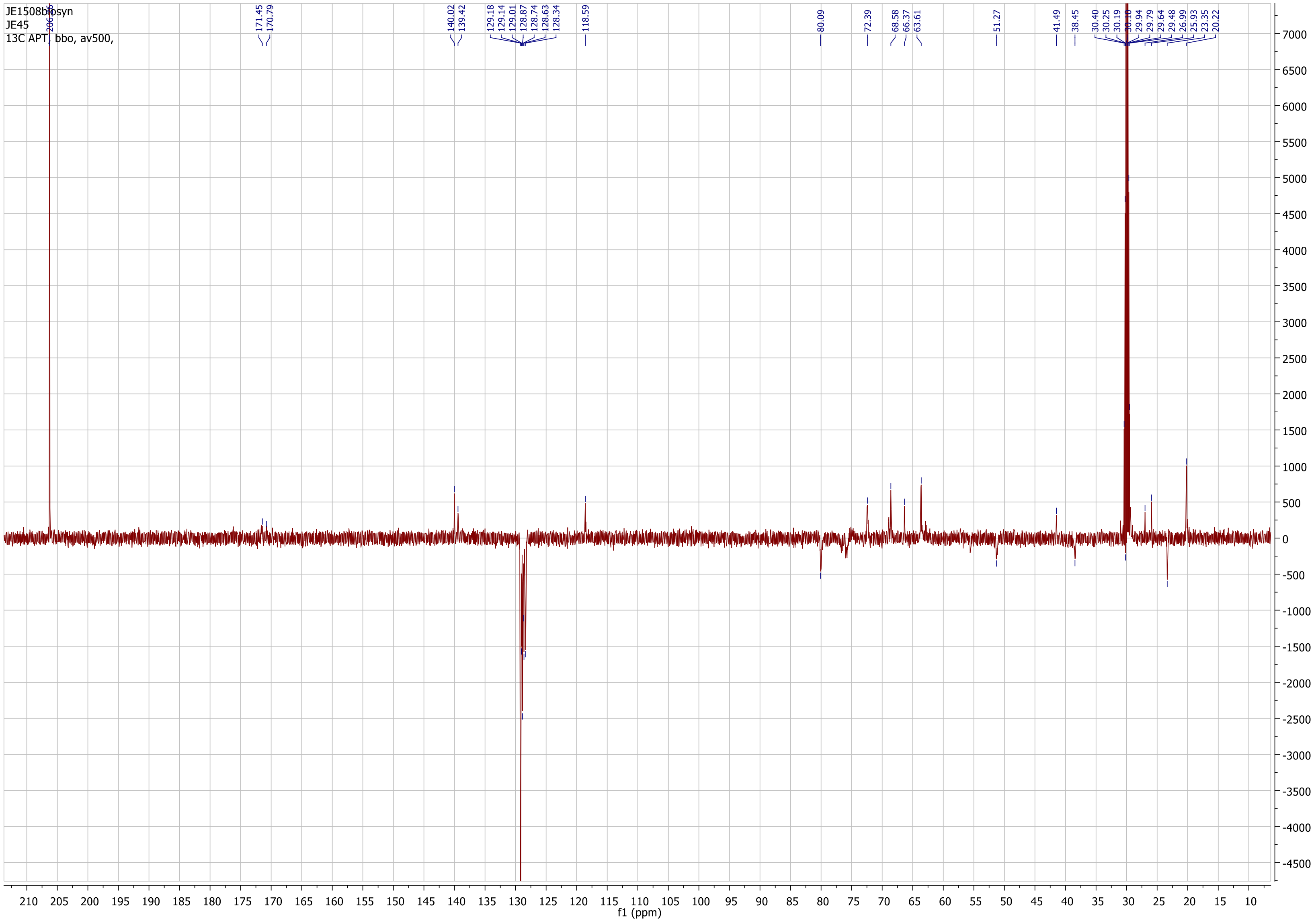

Supplementary Figure 35 1-O-octa-((2-Acetamido-3,4-di-O-benzyl-2-deoxy-5a-carba-α-D-mannopyranosyl-1-O-phosphoryl)2-cyanoethyl)-6-hexyl-benzyl-carbamate **22**

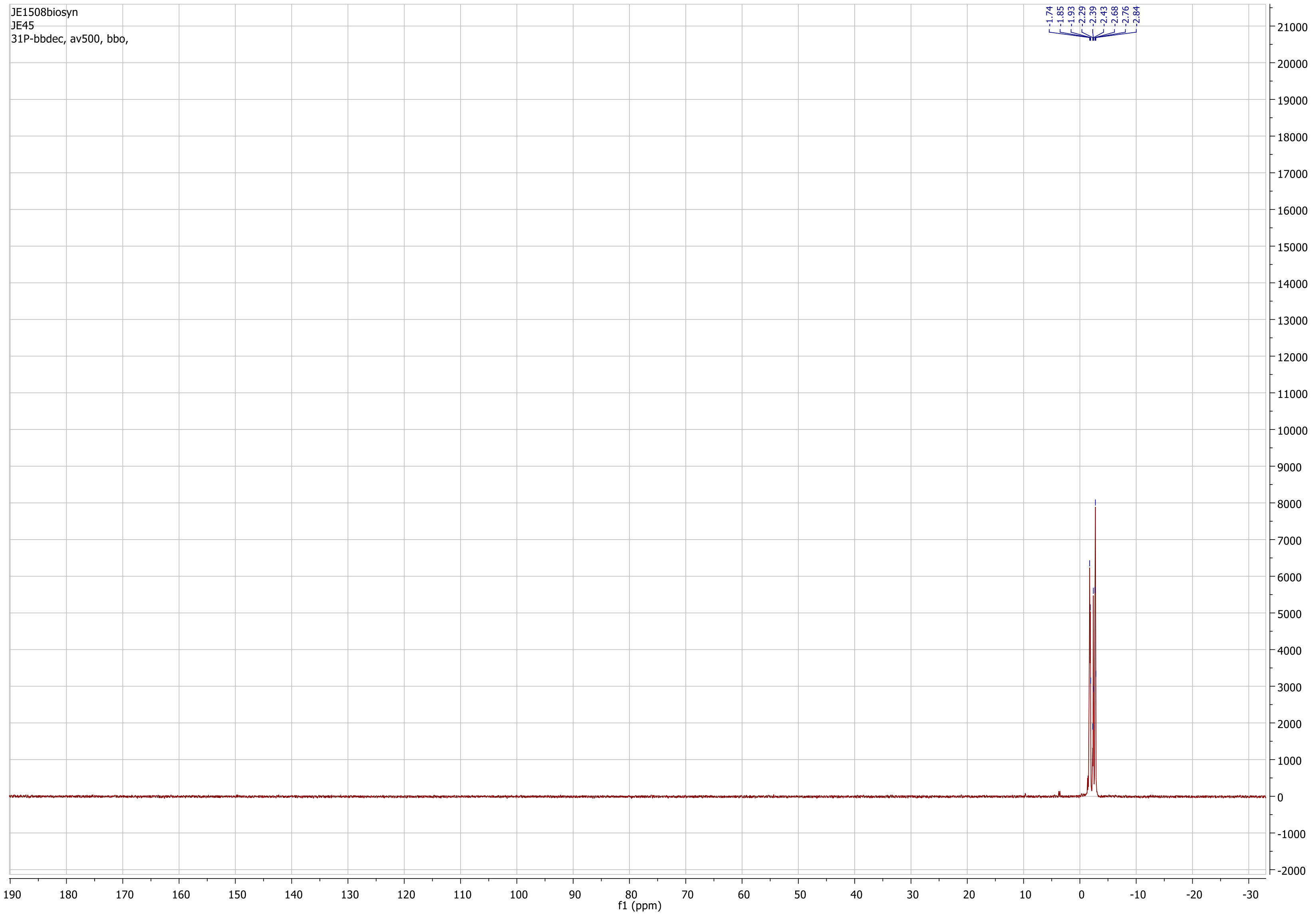

Supplementary Figure 36 1-O-octa-((2-Acetamido-3,4-di-O-benzyl-2-deoxy-5a-carba-α-D-mannopyranosyl-1-O-phosphoryl)2-cyanoethyl)-6-hexyl-benzyl-carbamate **22**

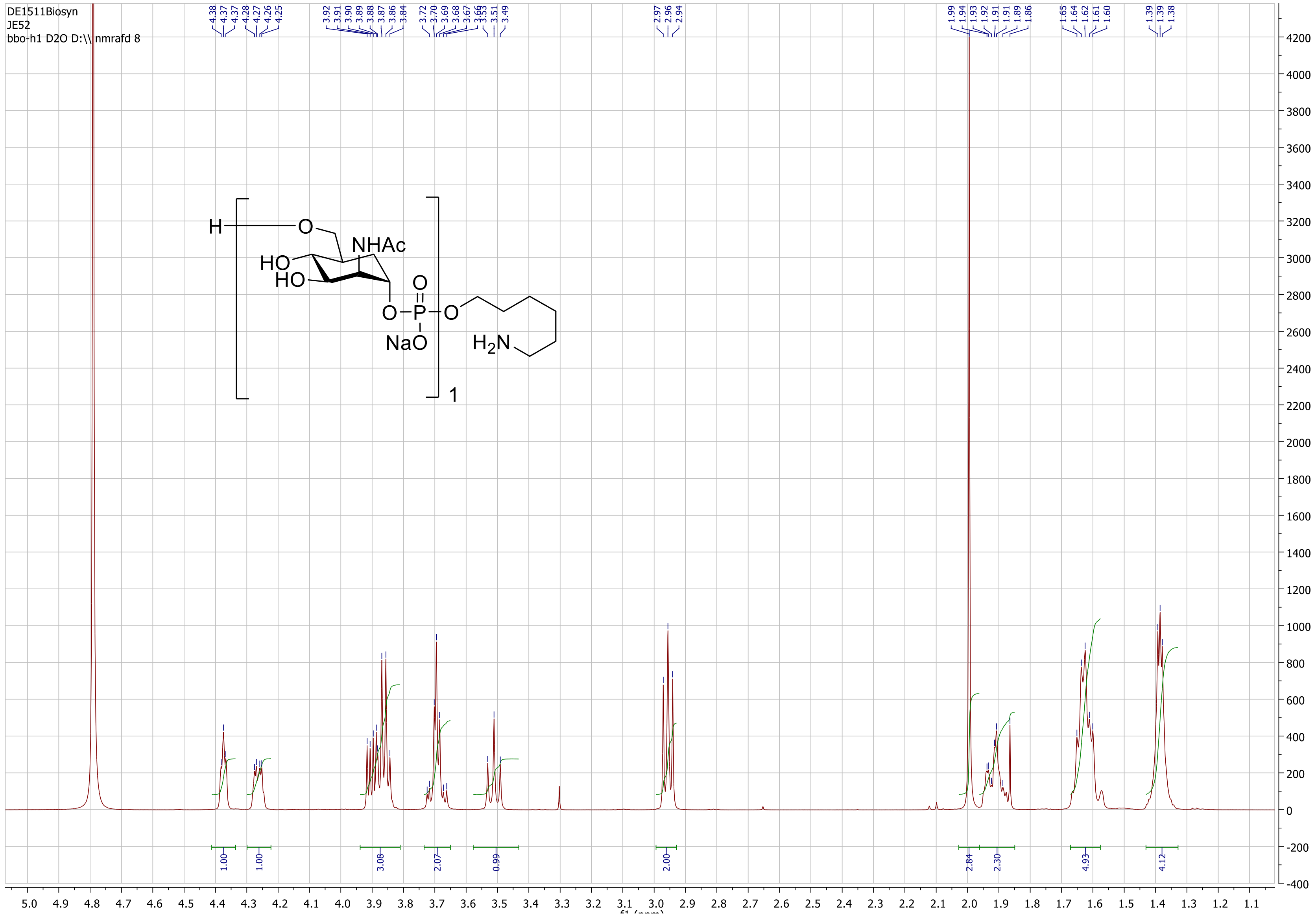

Supplementary Figure 37 1-O-(2-Acetamido-2-deoxy-5a-carba-α-D-mannopyranosyl-1-O-phosphoryl)-6-hexyl-amine **1**

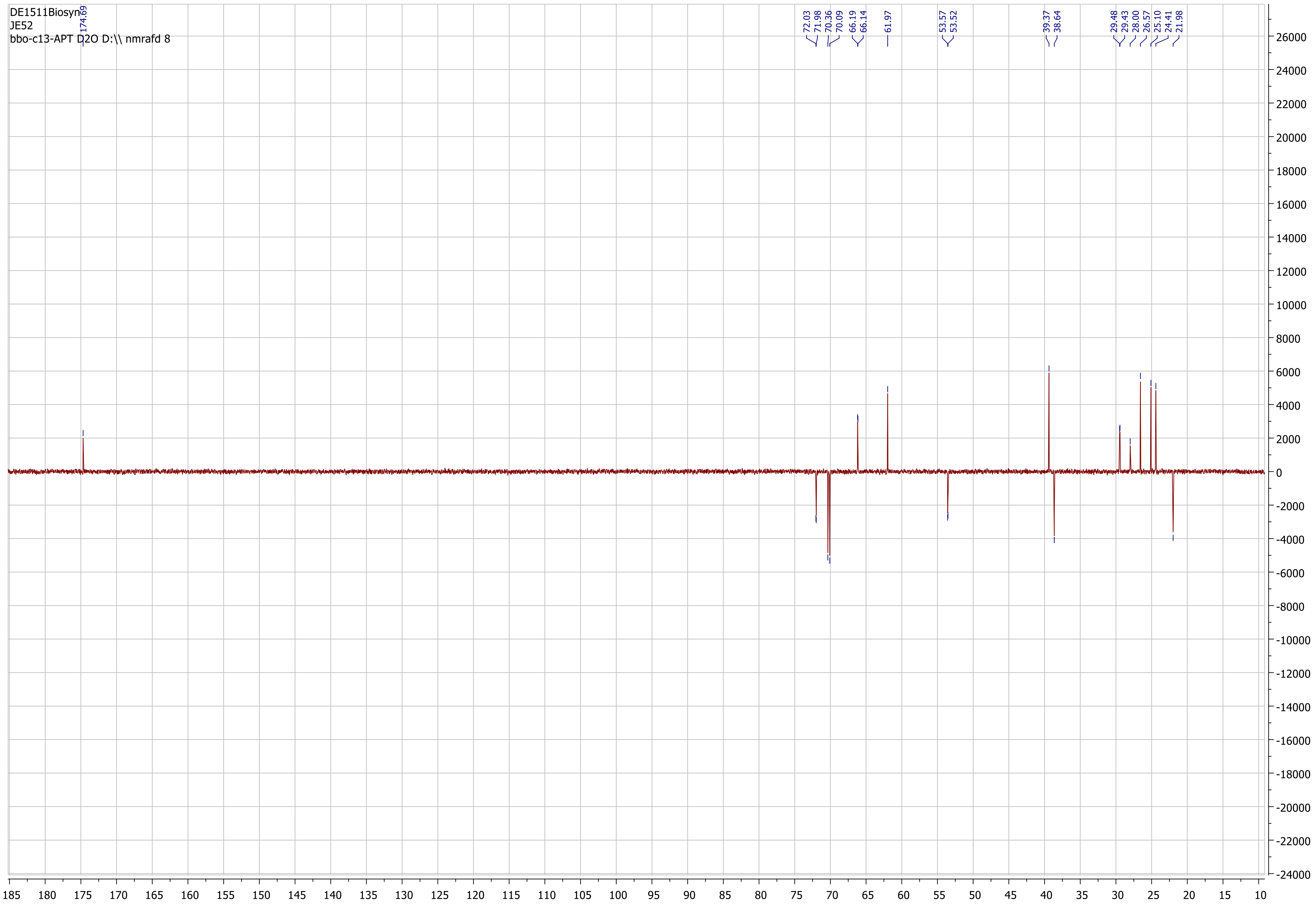

Supplementary Figure 38 1-O-(2-Acetamido-2-deoxy-5a-carba-α-D-mannopyranosyl-1-O-phosphoryl)-6-hexyl-amine **1**

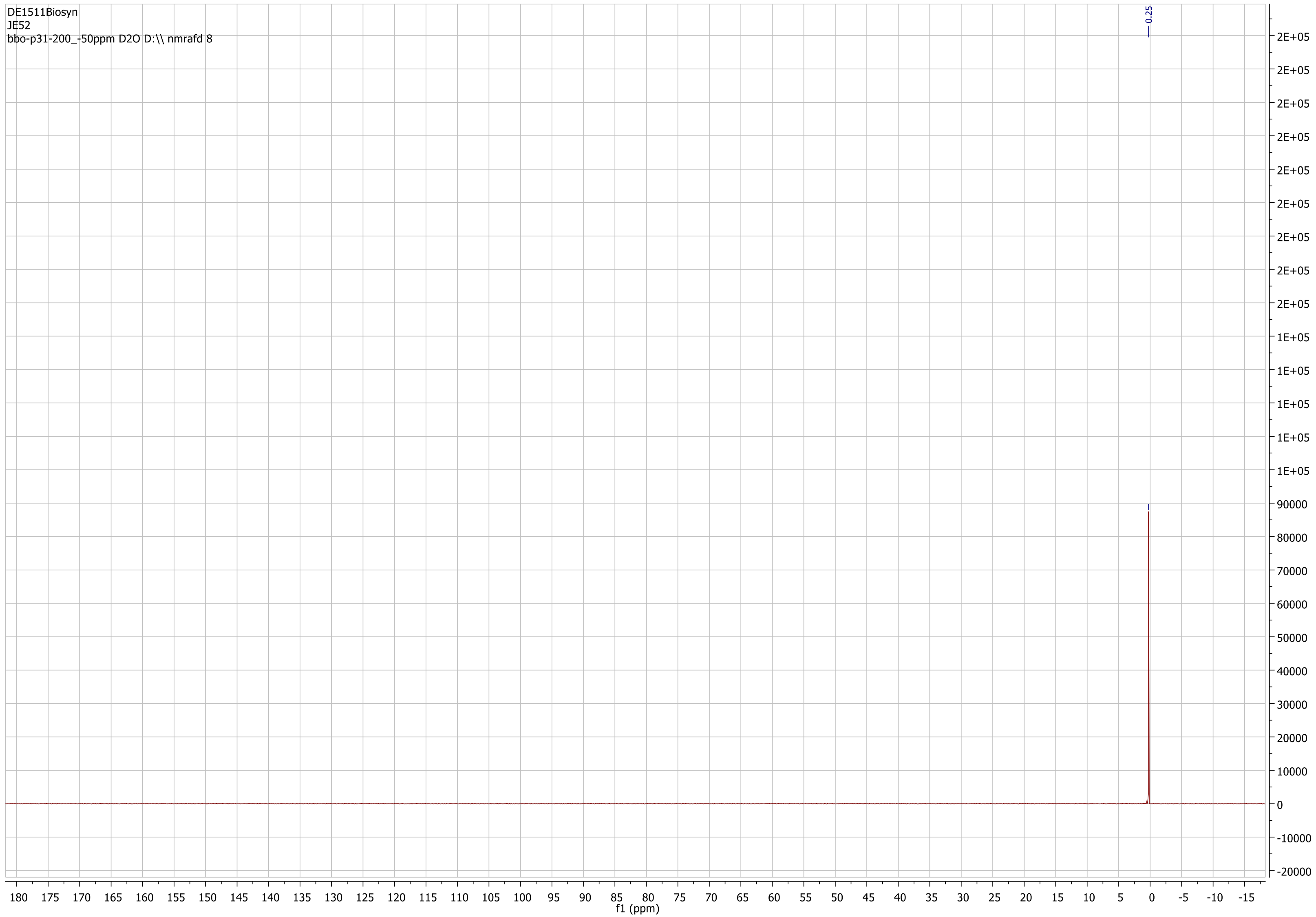

Supplementary Figure 39 1-O-(2-Acetamido-2-deoxy-5a-carba-α-D-mannopyranosyl-1-O-phosphoryl)-6-hexyl-amine **1**

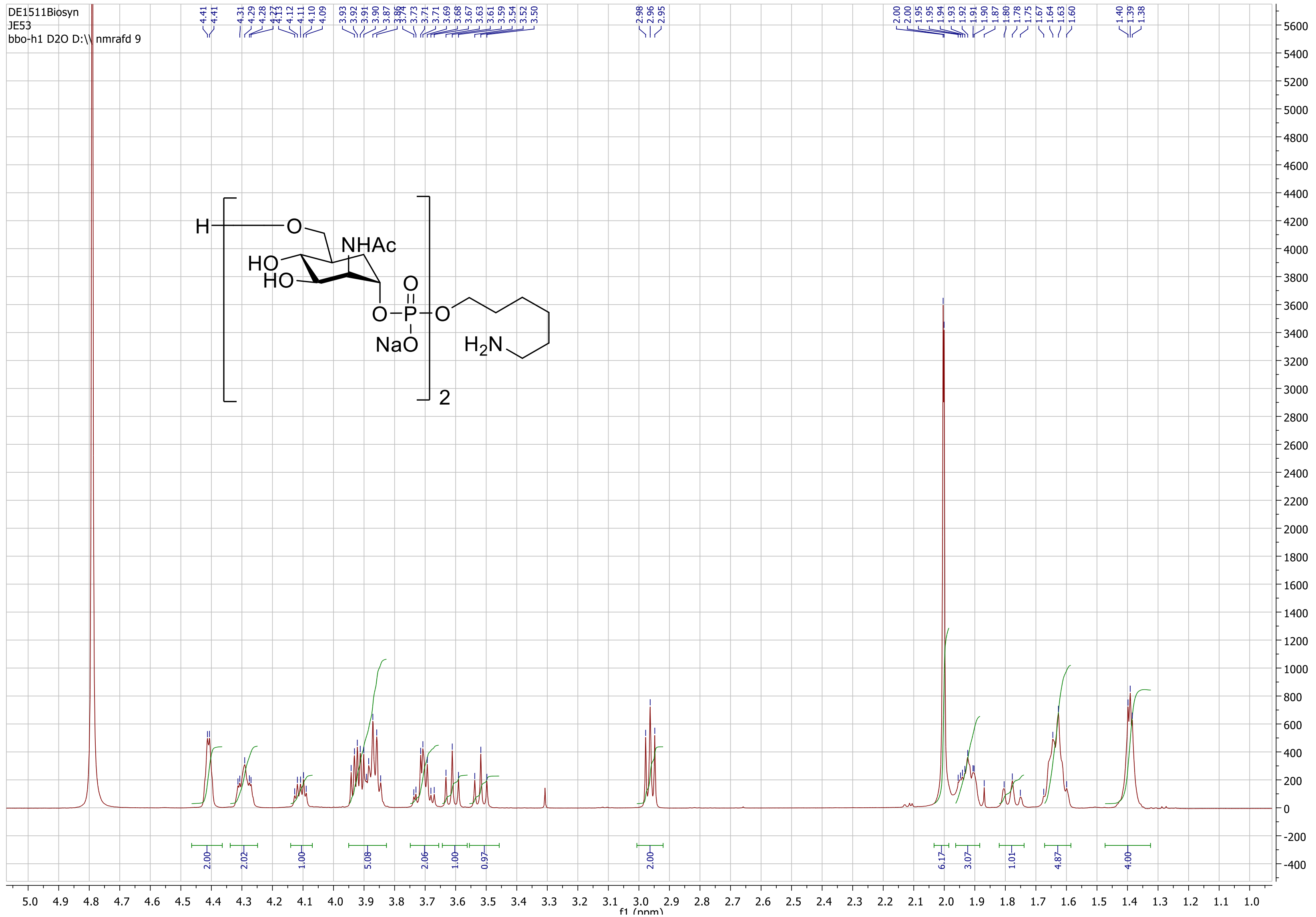

Supplementary Figure 40 1-O-di-(2-Acetamido-2-deoxy-5a-carba-α-D-mannopyranosyl-1-O-phosphoryl)-6-hexyl-amine **2**

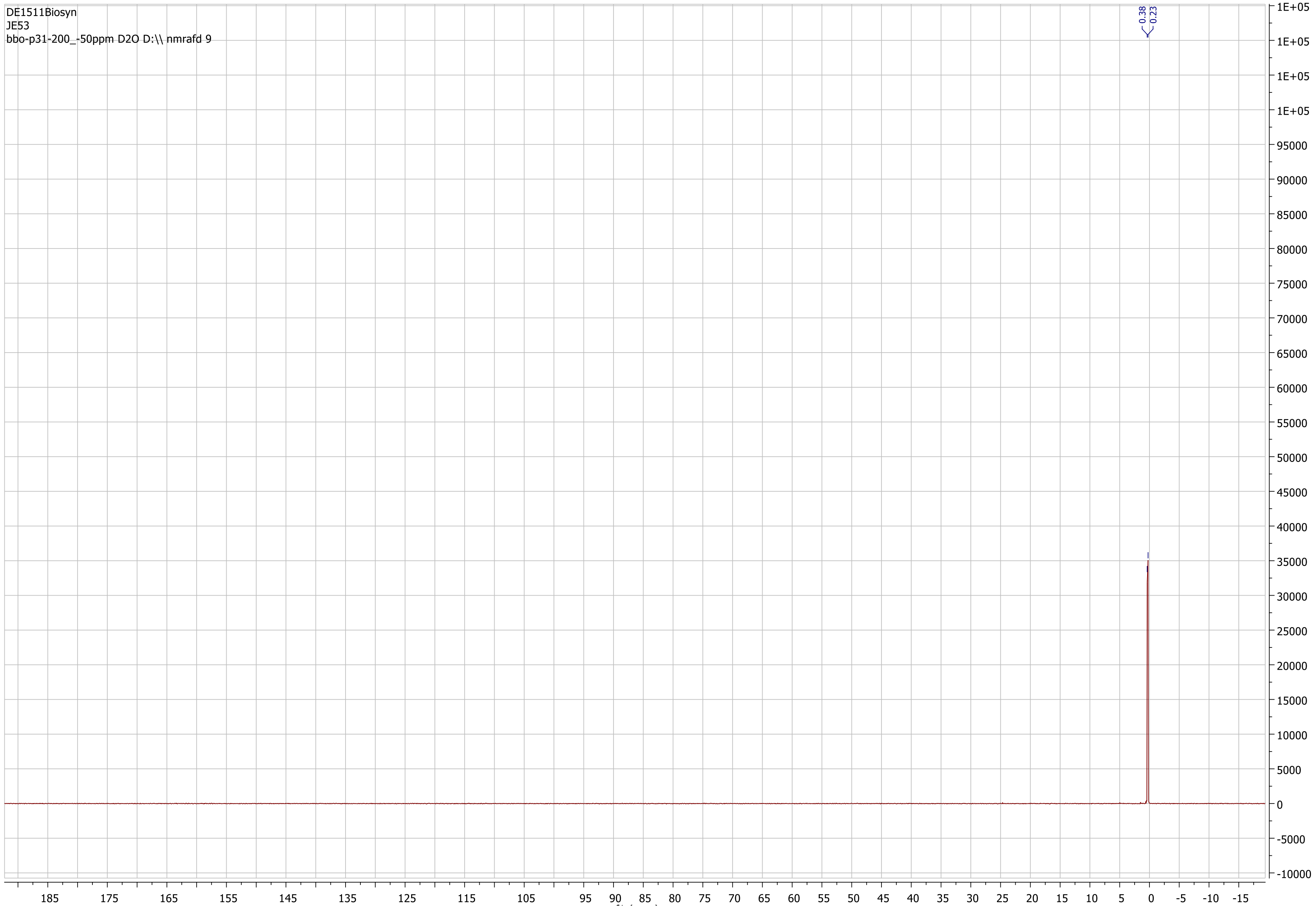

*Supplementary Figure 41 1-O-di-(2-Acetamido-2-deoxy-5a-carba-α-D-mannopyranosyl-1-O-phosphoryl)-6-hexyl-amine **2***

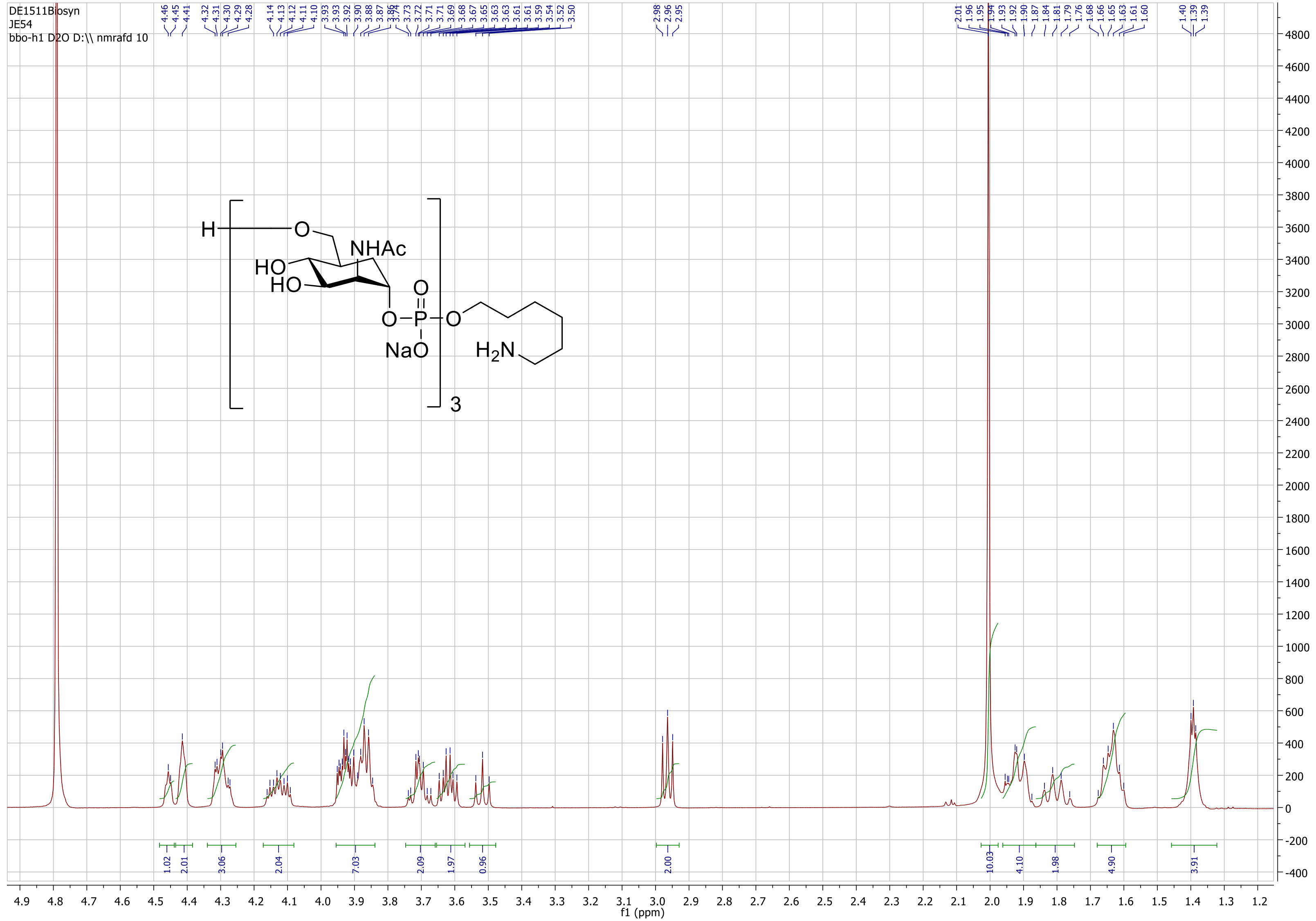

Supplementary Figure 42 1-O-tri-(2-Acetamido-2-deoxy-5a-carba-α-D-mannopyranosyl-1-O-phosphoryl)-6-hexyl-amine **3**

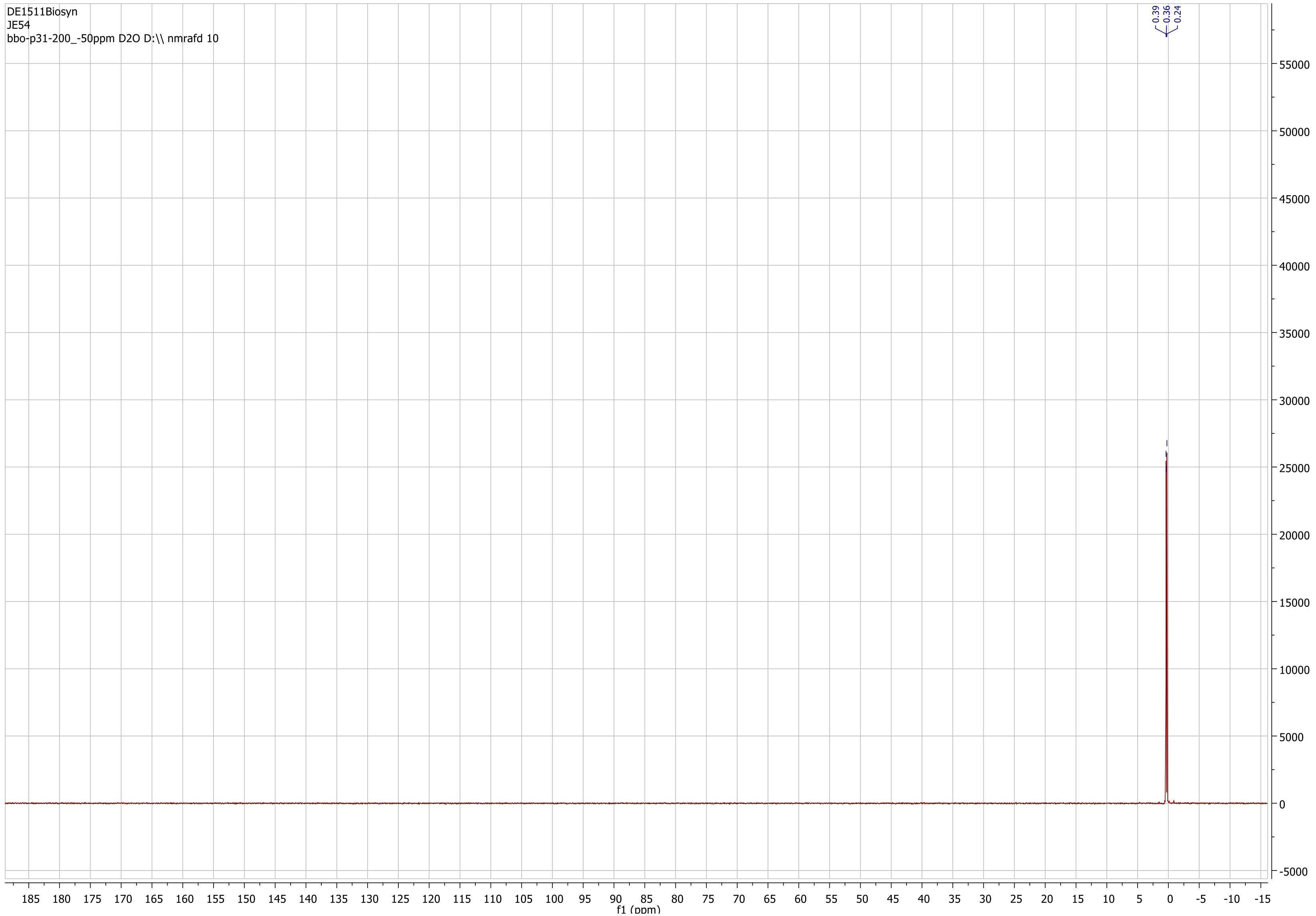

Supplementary Figure 43 1-O-tri-(2-Acetamido-2-deoxy-5a-carba-α-D-mannopyranosyl-1-O-phosphoryl)-6-hexyl-amine **3**

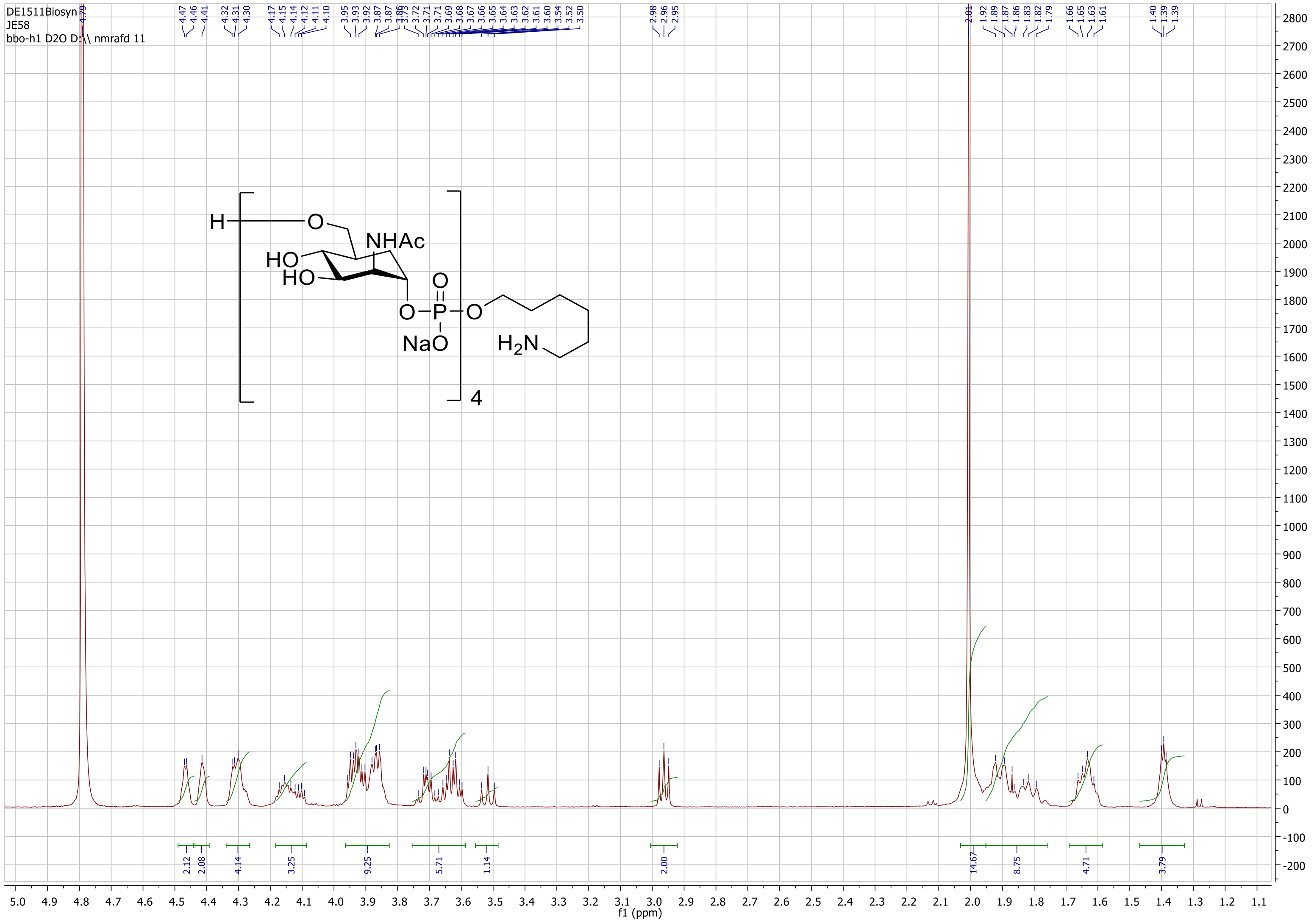

Supplementary Figure 44 1-O-tetra-(2-Acetamido-2-deoxy-5a-carba-α-D-mannopyranosyl-1-O-phosphoryl)-6-hexyl-amine **4**

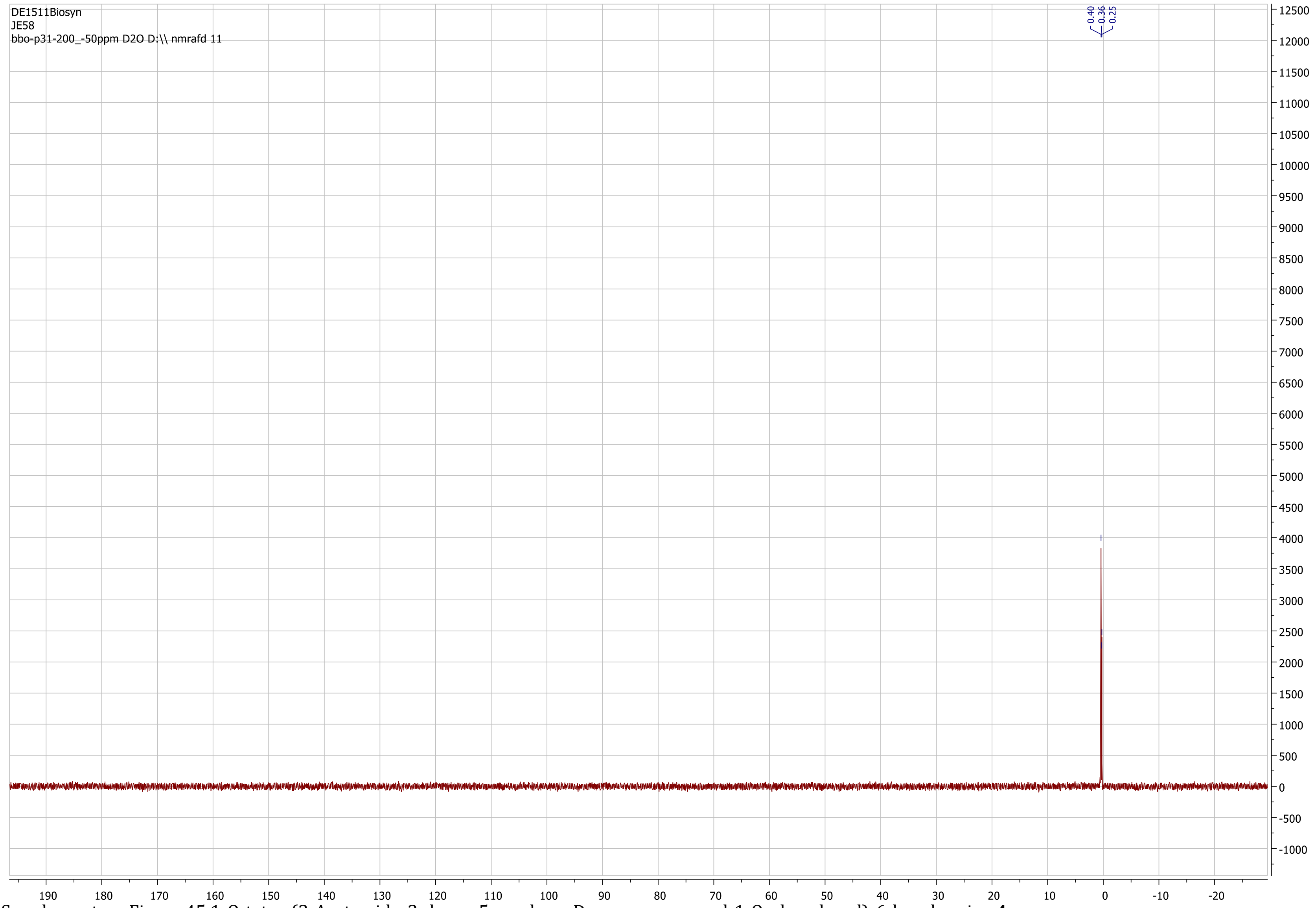

Supplementary Figure 45 1-O-tetra-(2-Acetamido-2-deoxy-5a-carba-α-D-mannopyranosyl-1-O-phosphoryl)-6-hexyl-amine **4**

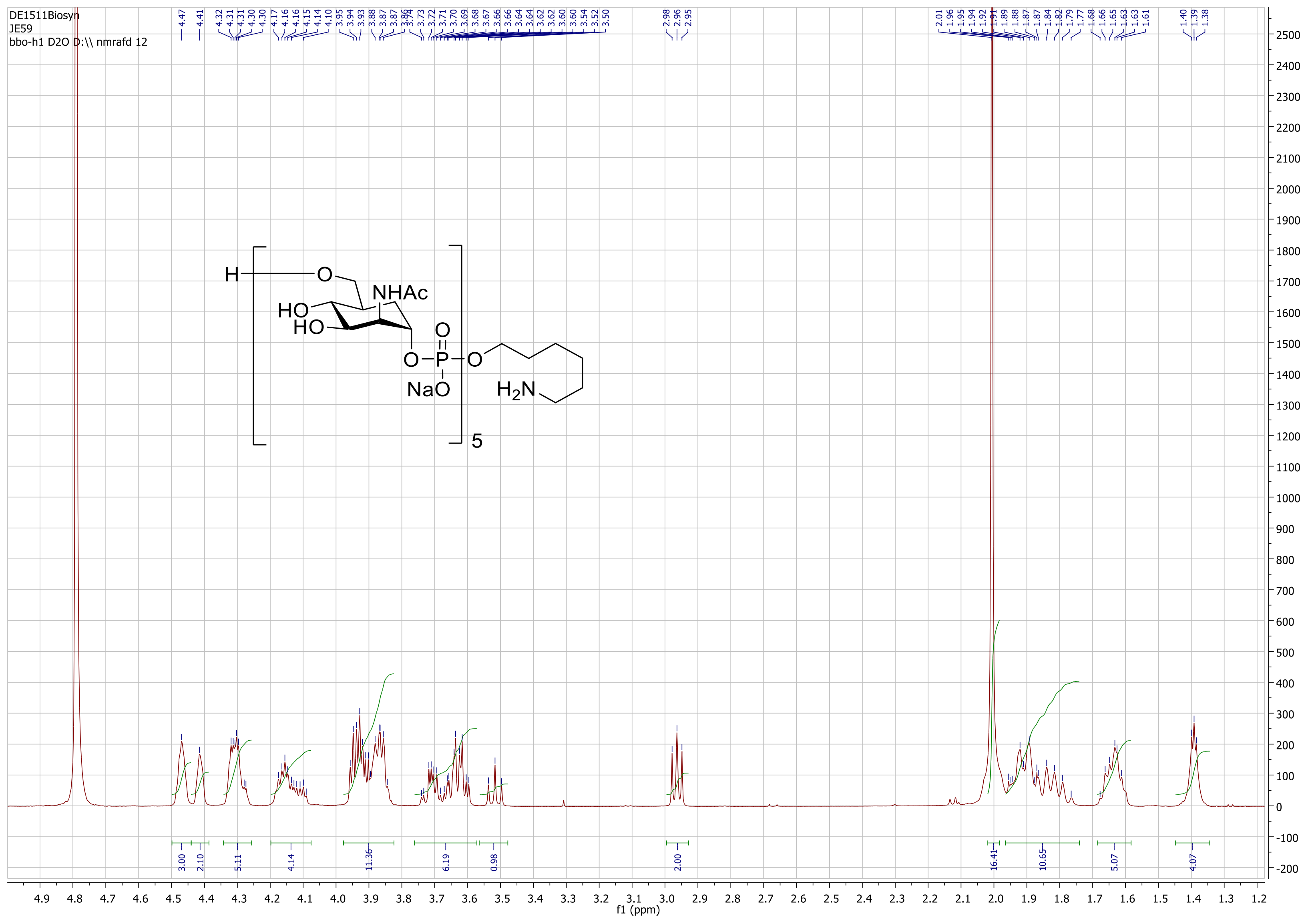

Supplementary Figure 46 1-O-penta-(2-Acetamido-2-deoxy-5a-carba-α-D-mannopyranosyl-1-O-phosphoryl)-6-hexyl-amine **5**

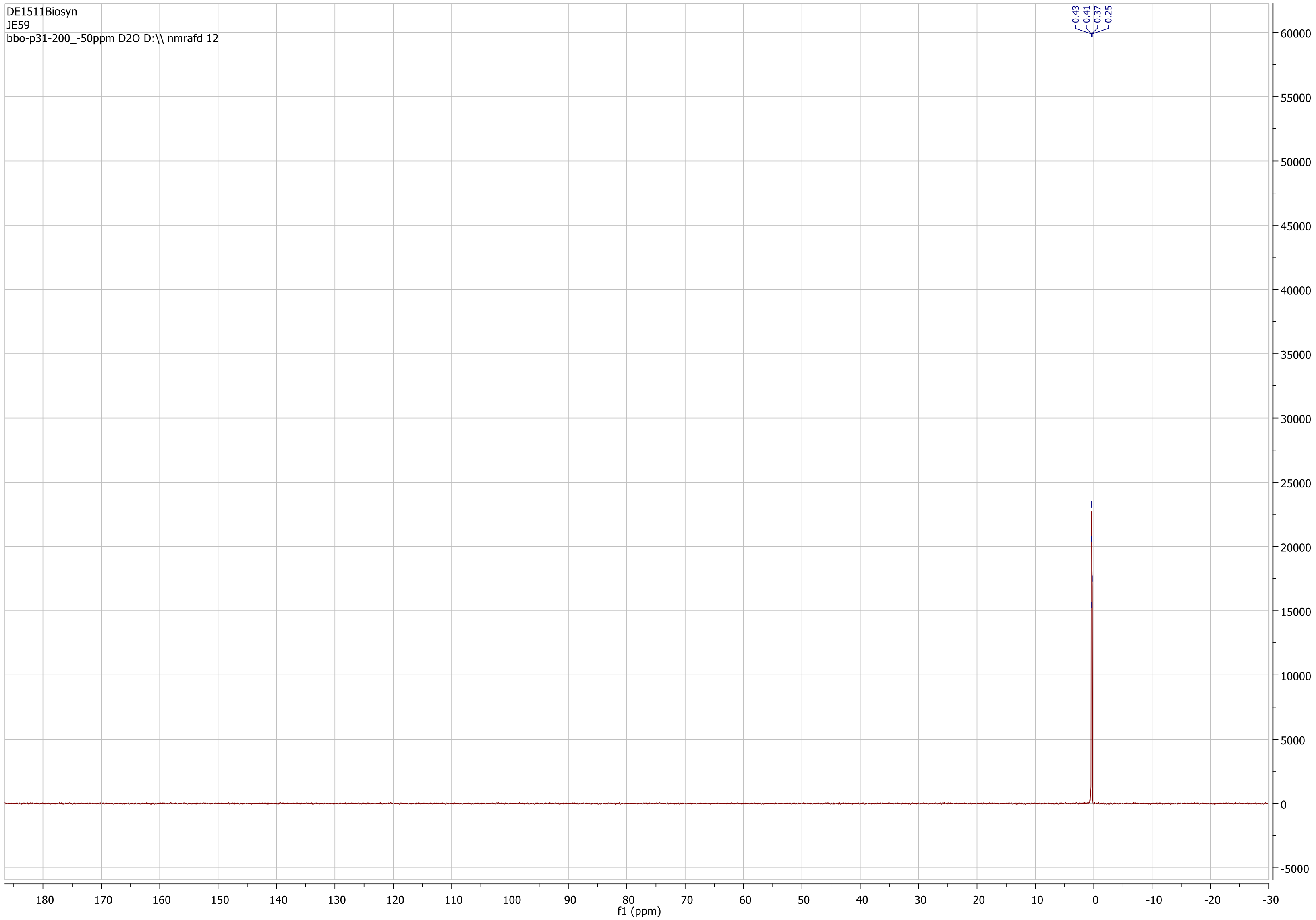

Supplementary Figure 47 1-O-penta-(2-Acetamido-2-deoxy-5a-carba-α-D-mannopyranosyl-1-O-phosphoryl)-6-hexyl-amine **5**

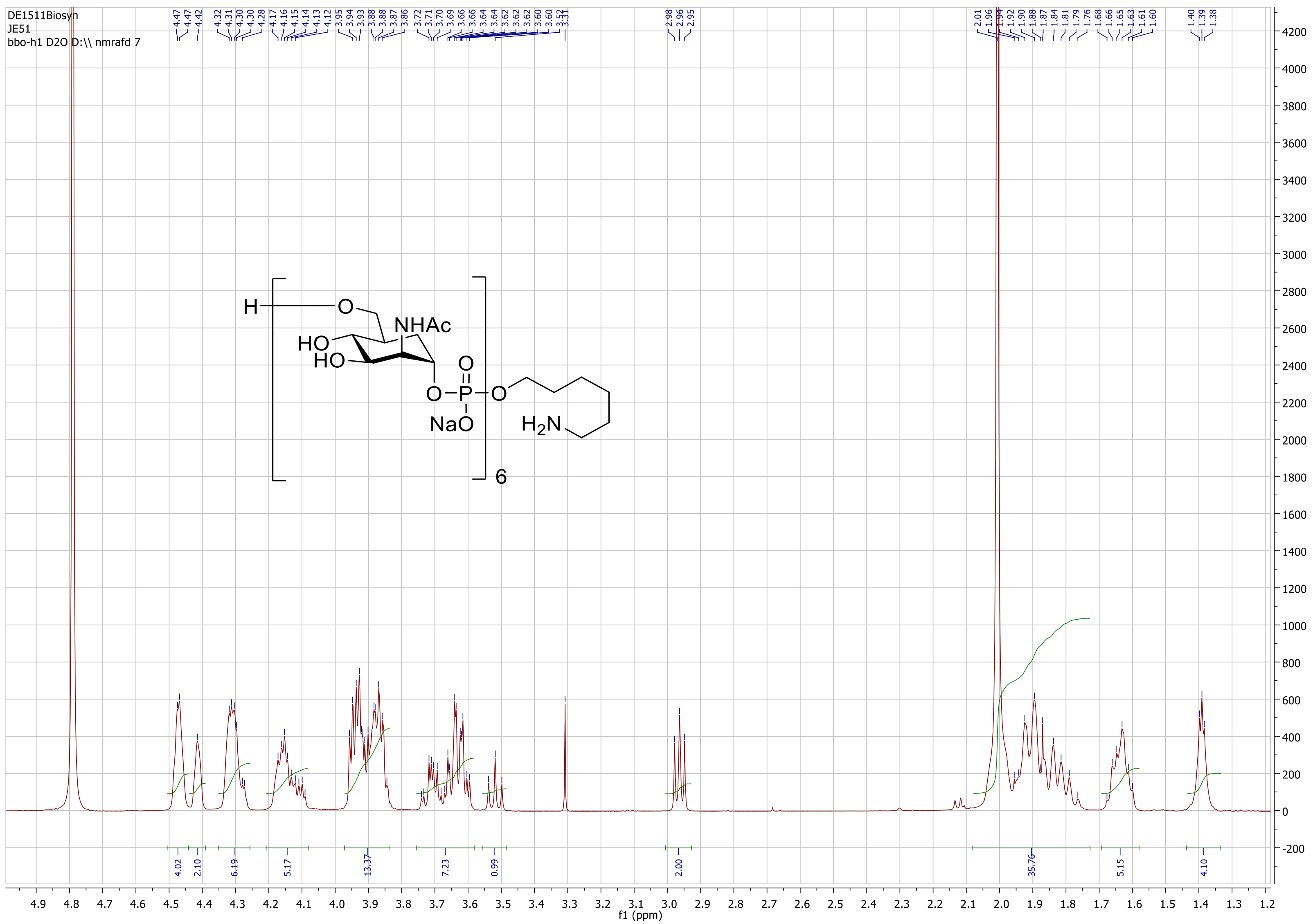

Supplementary Figure 48 1-O-hexa-(2-Acetamido-2-deoxy-5a-carba-α-D-mannopyranosyl-1-O-phosphoryl)-6-hexyl-amine **6**

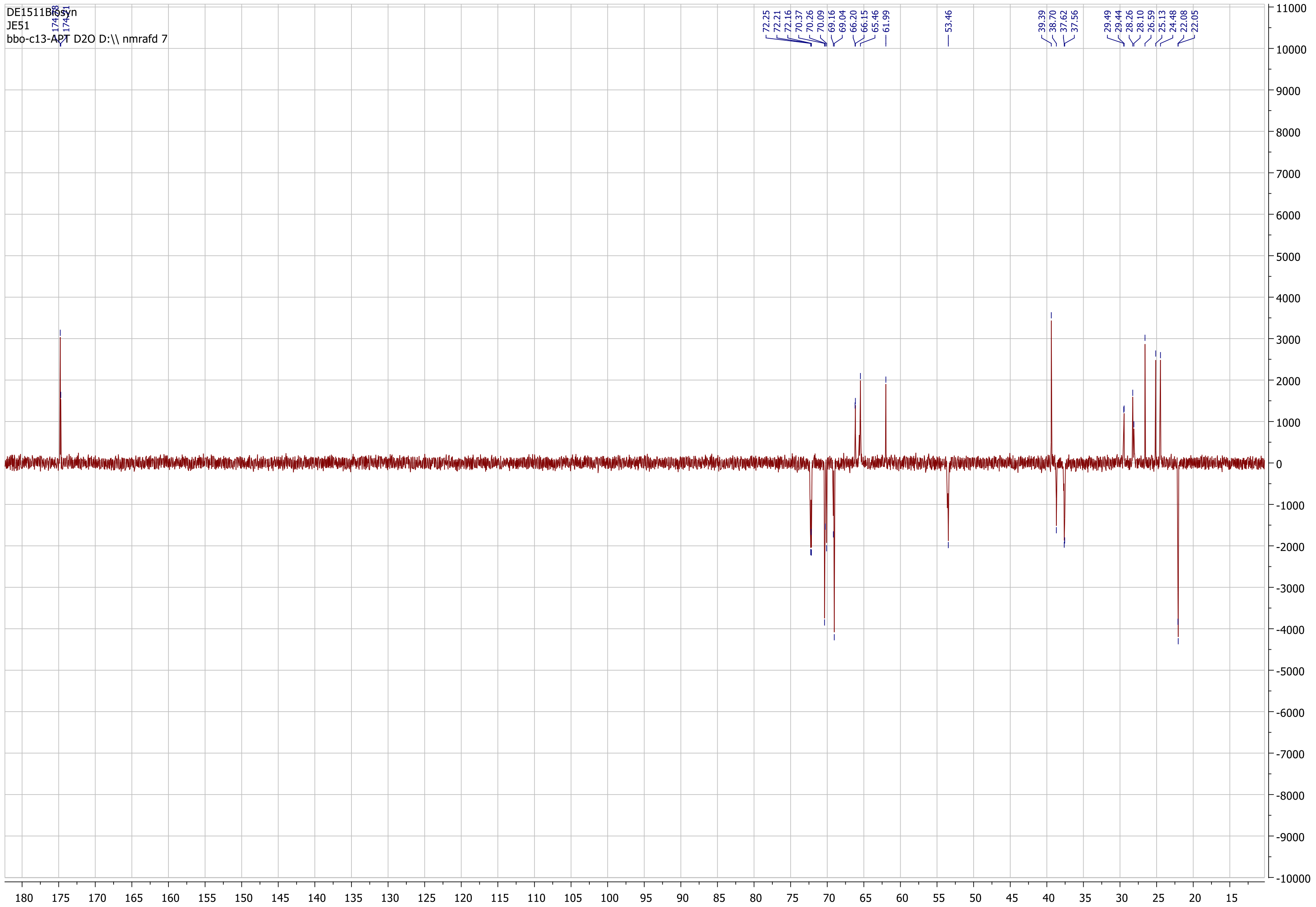

Supplementary Figure 49 1-O-hexa-(2-Acetamido-2-deoxy-5a-carba-α-D-mannopyranosyl-1-O-phosphoryl)-6-hexyl-amine **6**

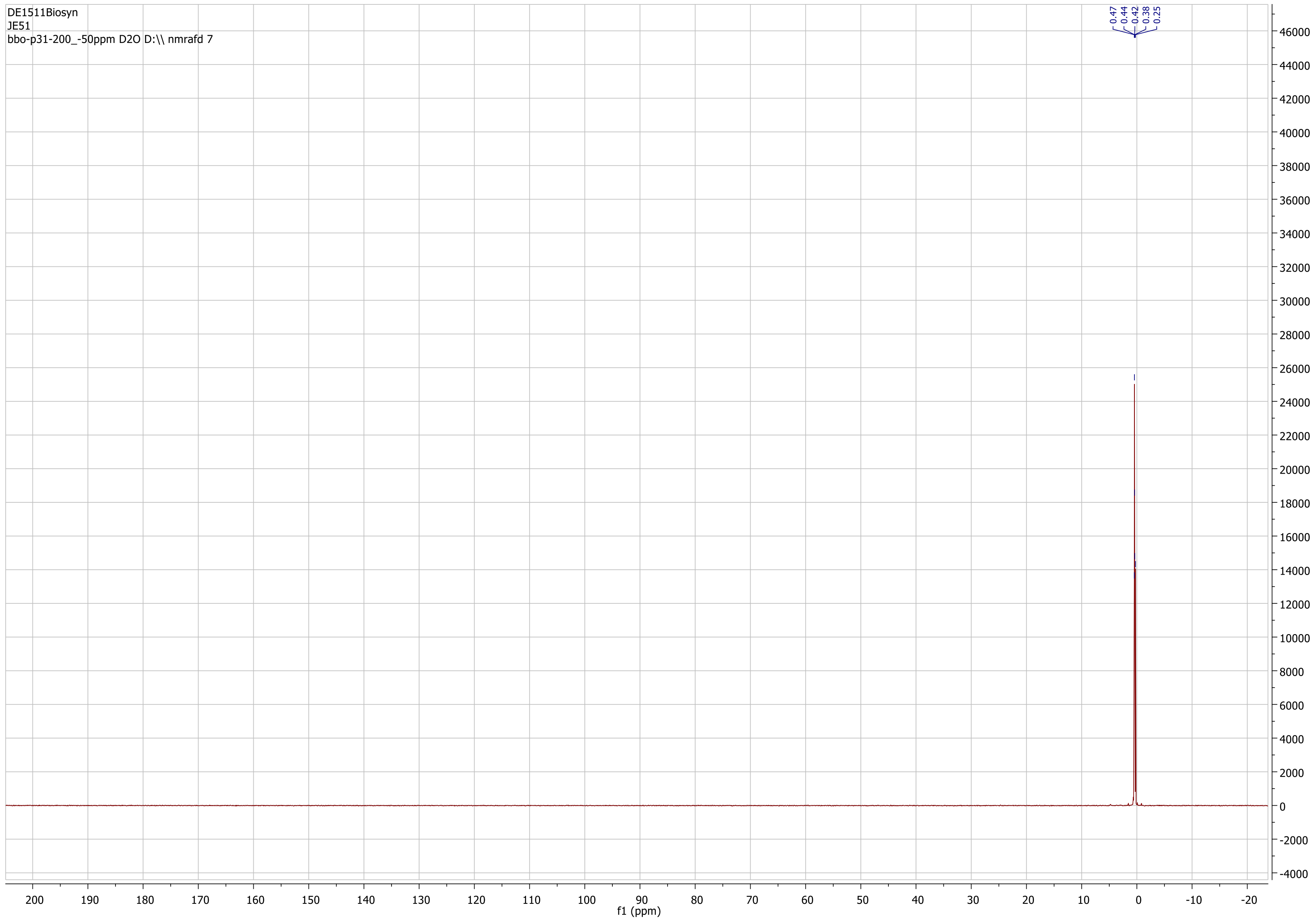

Supplementary Figure 50 1-O-hexa-(2-Acetamido-2-deoxy-5a-carba-α-D-mannopyranosyl-1-O-phosphoryl)-6-hexyl-amine **6**

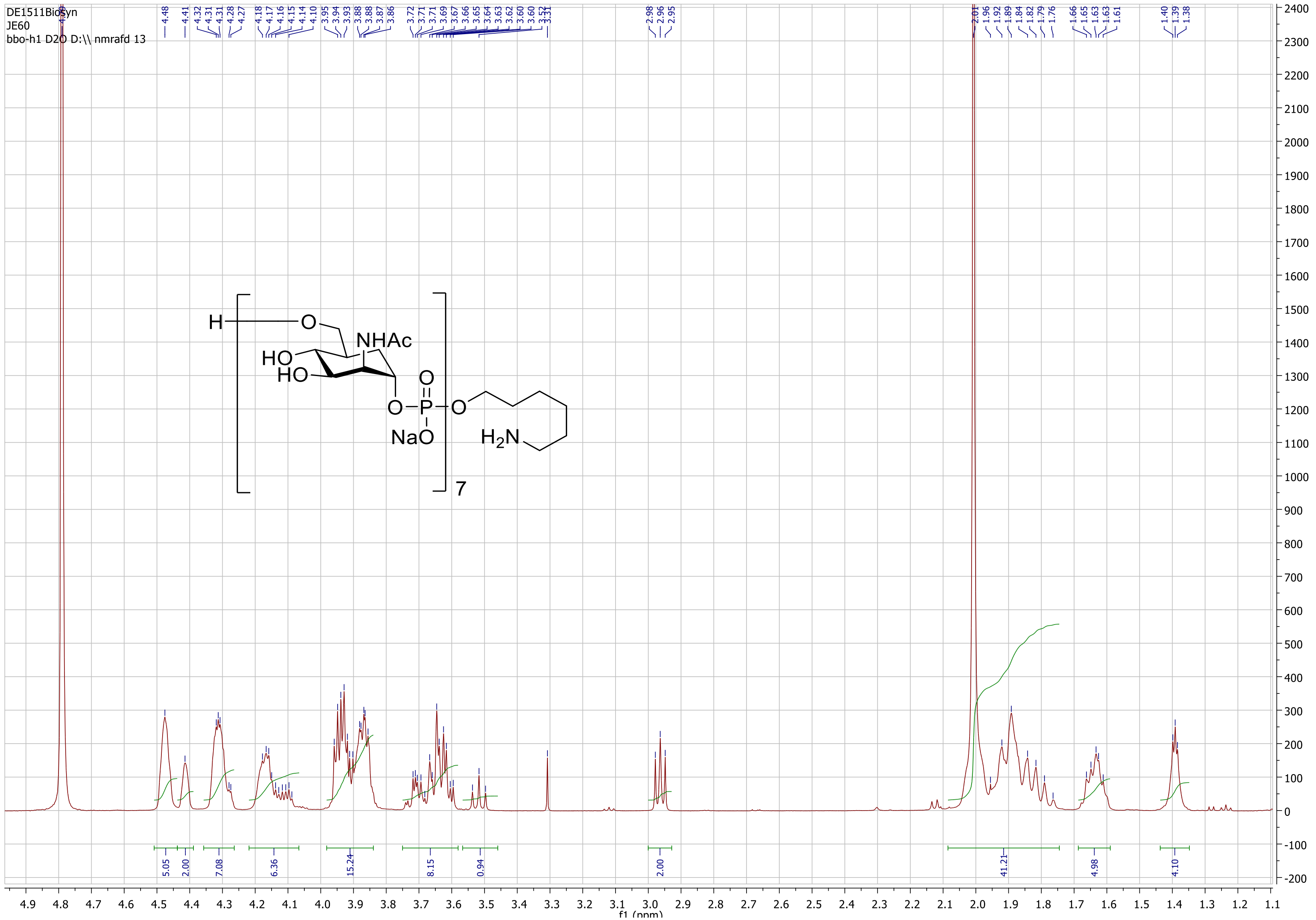

Supplementary Figure 51 1-O-epta-(2-Acetamido-2-deoxy-5a-carba-α-D-mannopyranosyl-1-O-phosphoryl)-6-hexyl-amine **7**

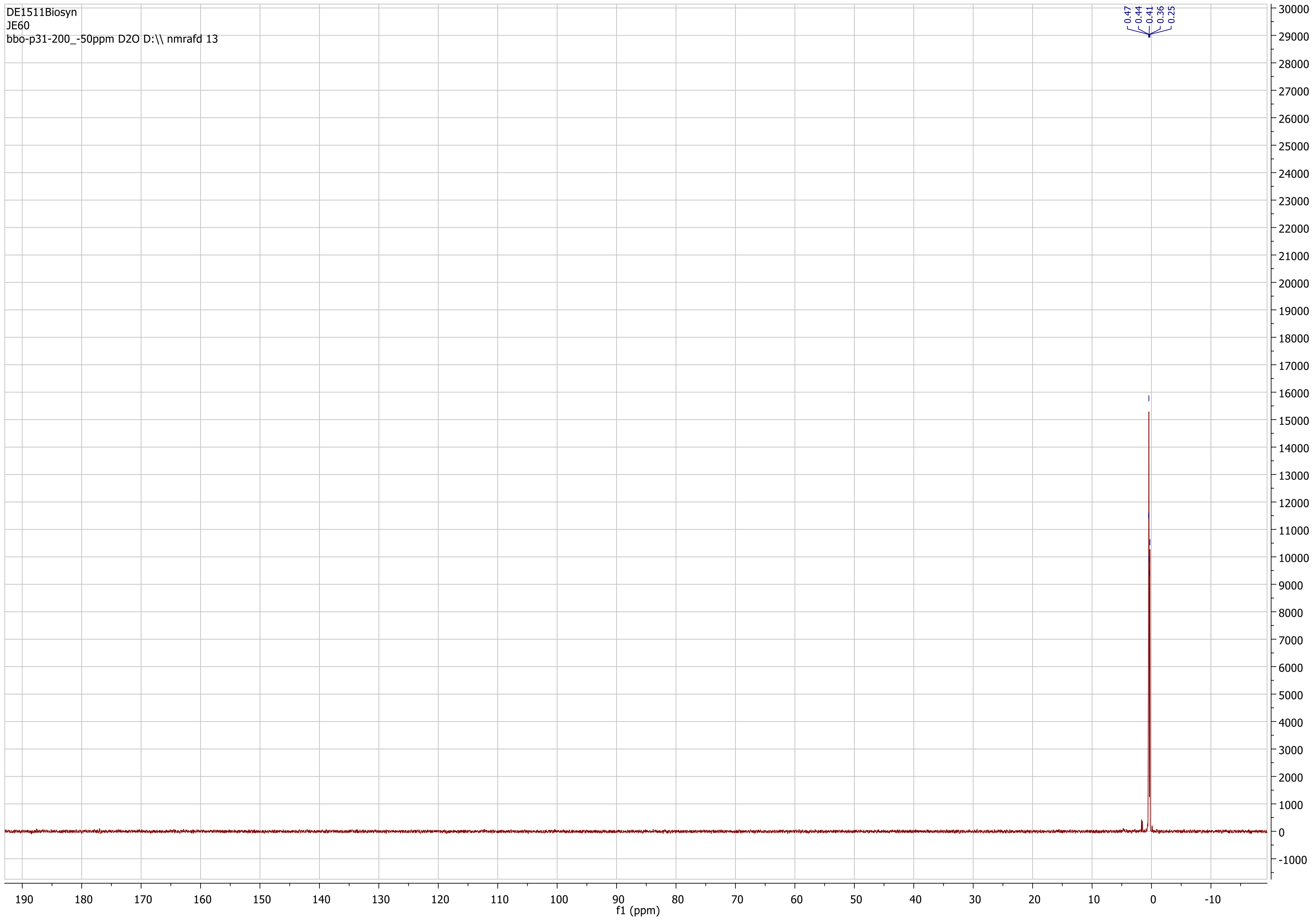

Supplementary Figure 52 1-O-epta-(2-Acetamido-2-deoxy-5a-carba-α-D-mannopyranosyl-1-O-phosphoryl)-6-hexyl-amine **7**

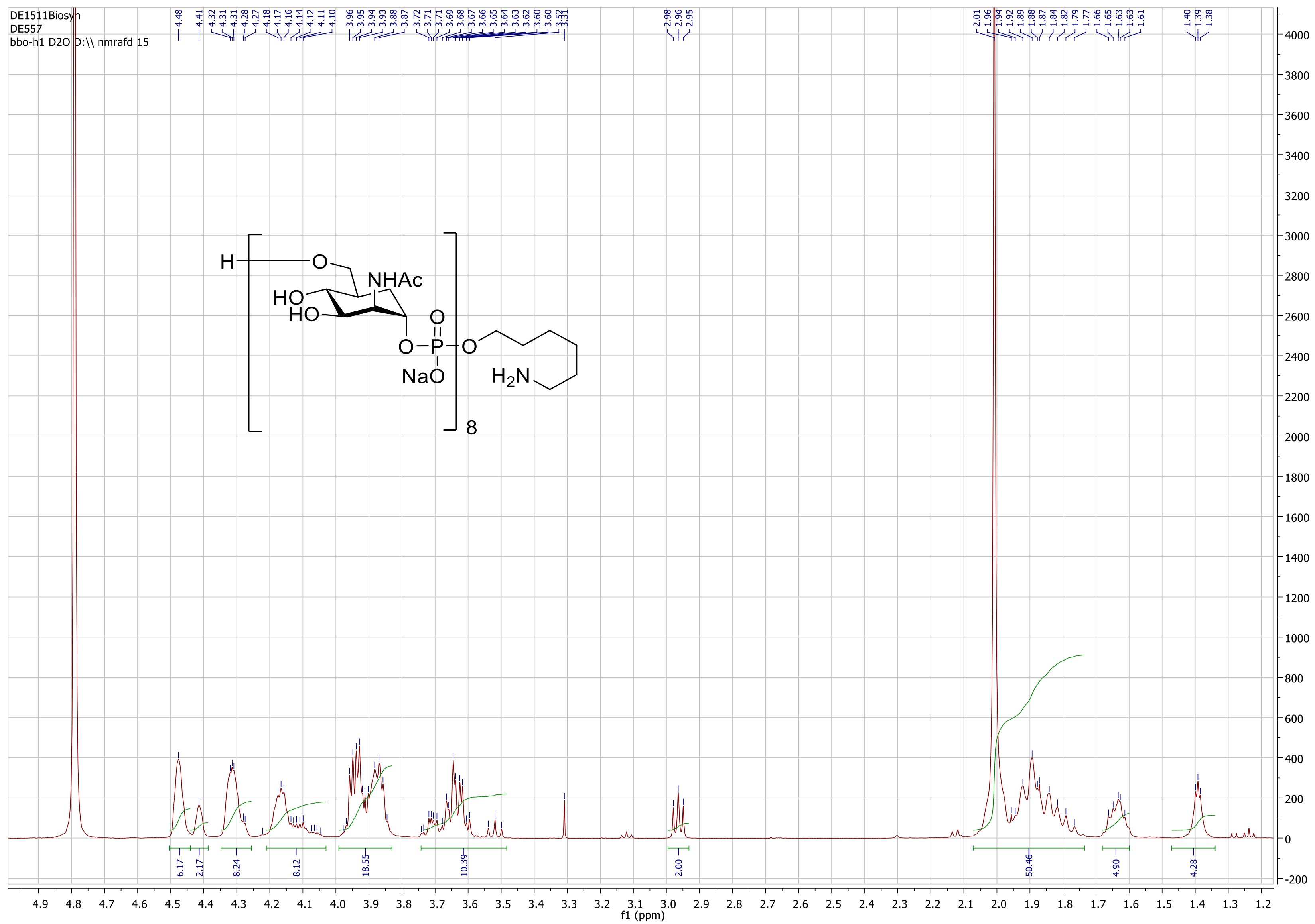

Supplementary Figure 53 1-O-octa-(2-Acetamido-2-deoxy-5a-carba-α-D-mannopyranosyl-1-O-phosphoryl)-6-hexyl-amine **8**

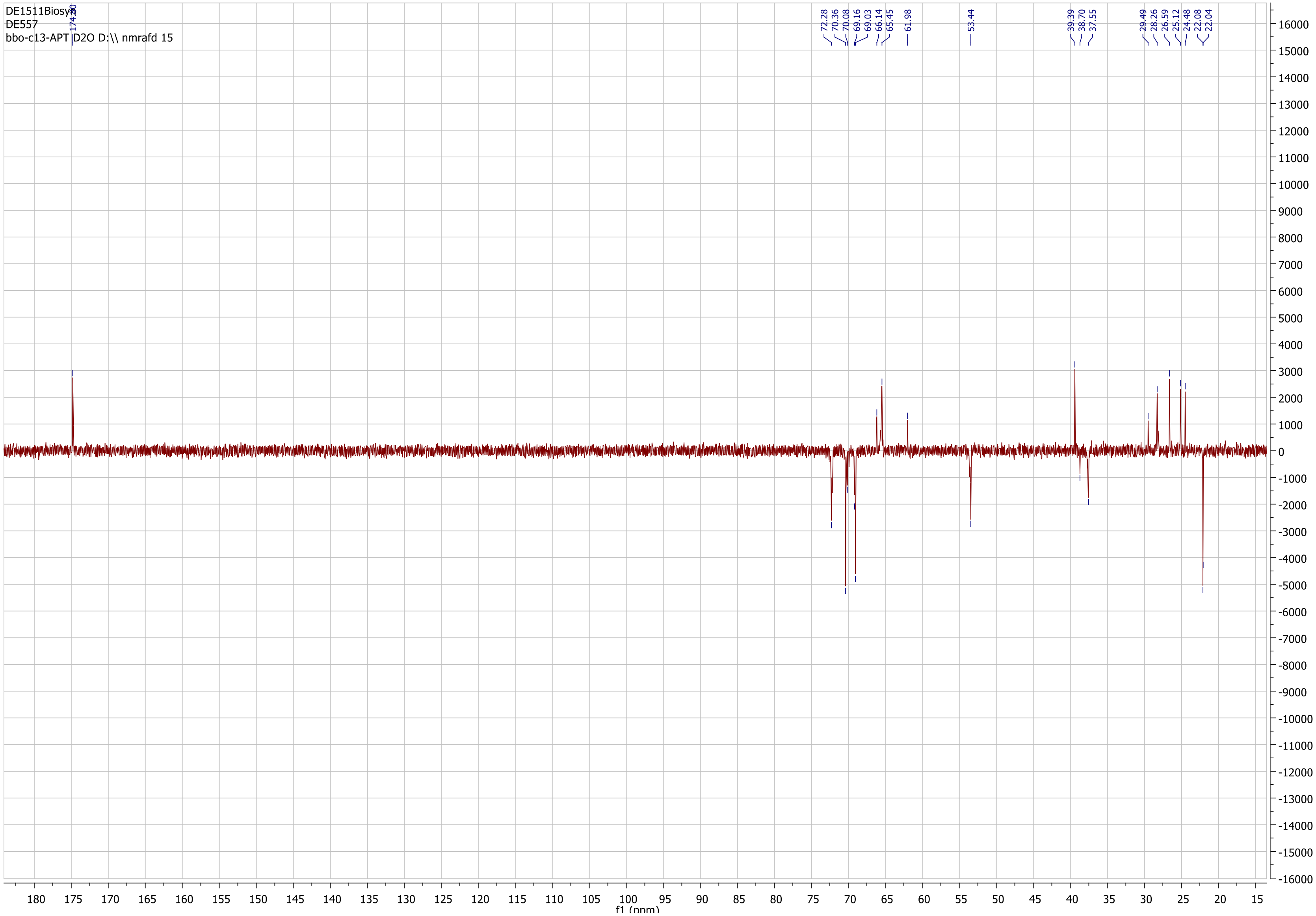

Supplementary Figure 54 1-O-octa-(2-Acetamido-2-deoxy-5a-carba-α-D-mannopyranosyl-1-O-phosphoryl)-6-hexyl-amine **8**

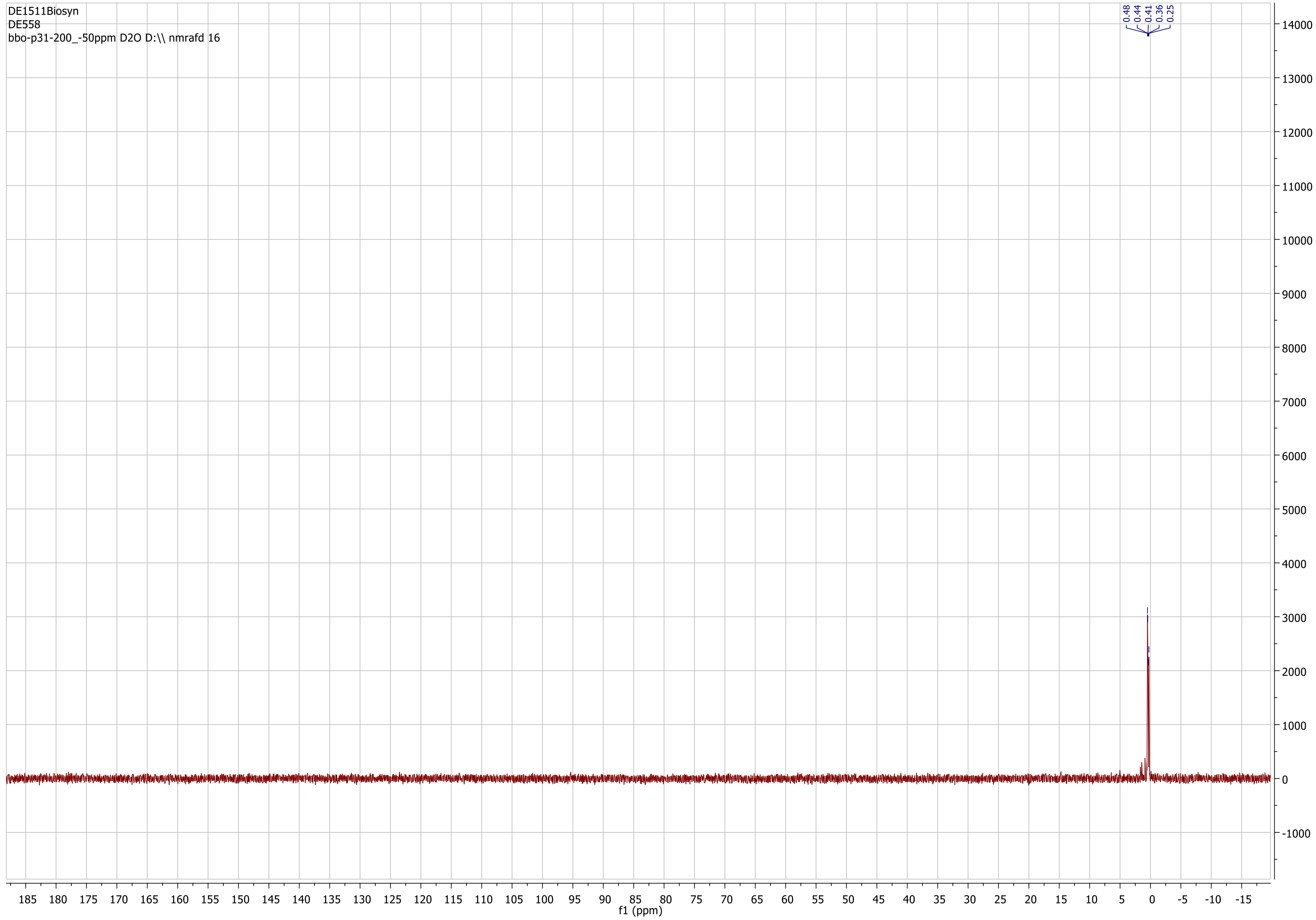

Supplementary Figure 55 1-O-octa-(2-Acetamido-2-deoxy-5a-carba-α-D-mannopyranosyl-1-O-phosphoryl)-6-hexyl-amine **8**

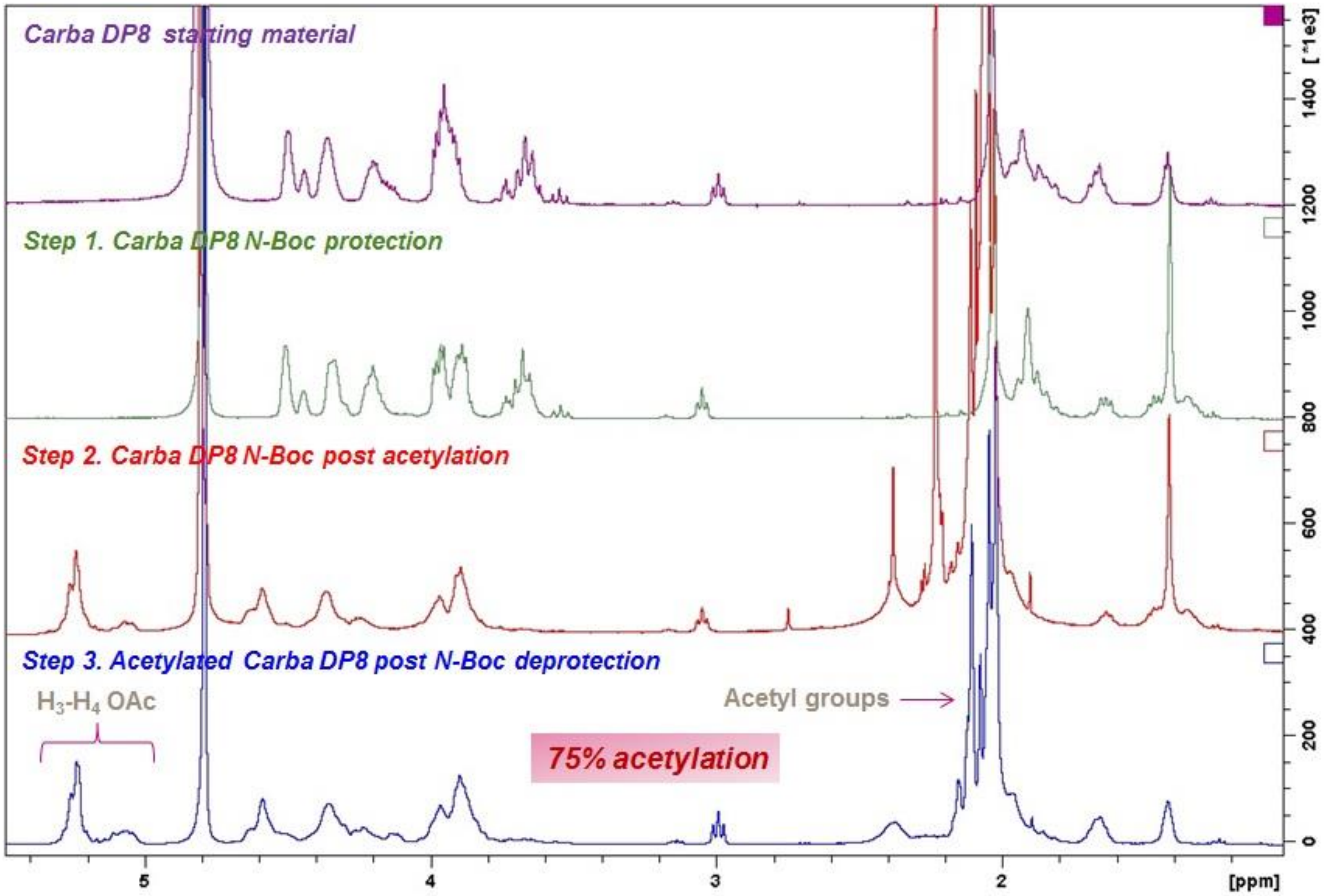

Supplementary Figure 56 ^{1}H NMR (400 MHz, D_2O) of compounds **8a-b**

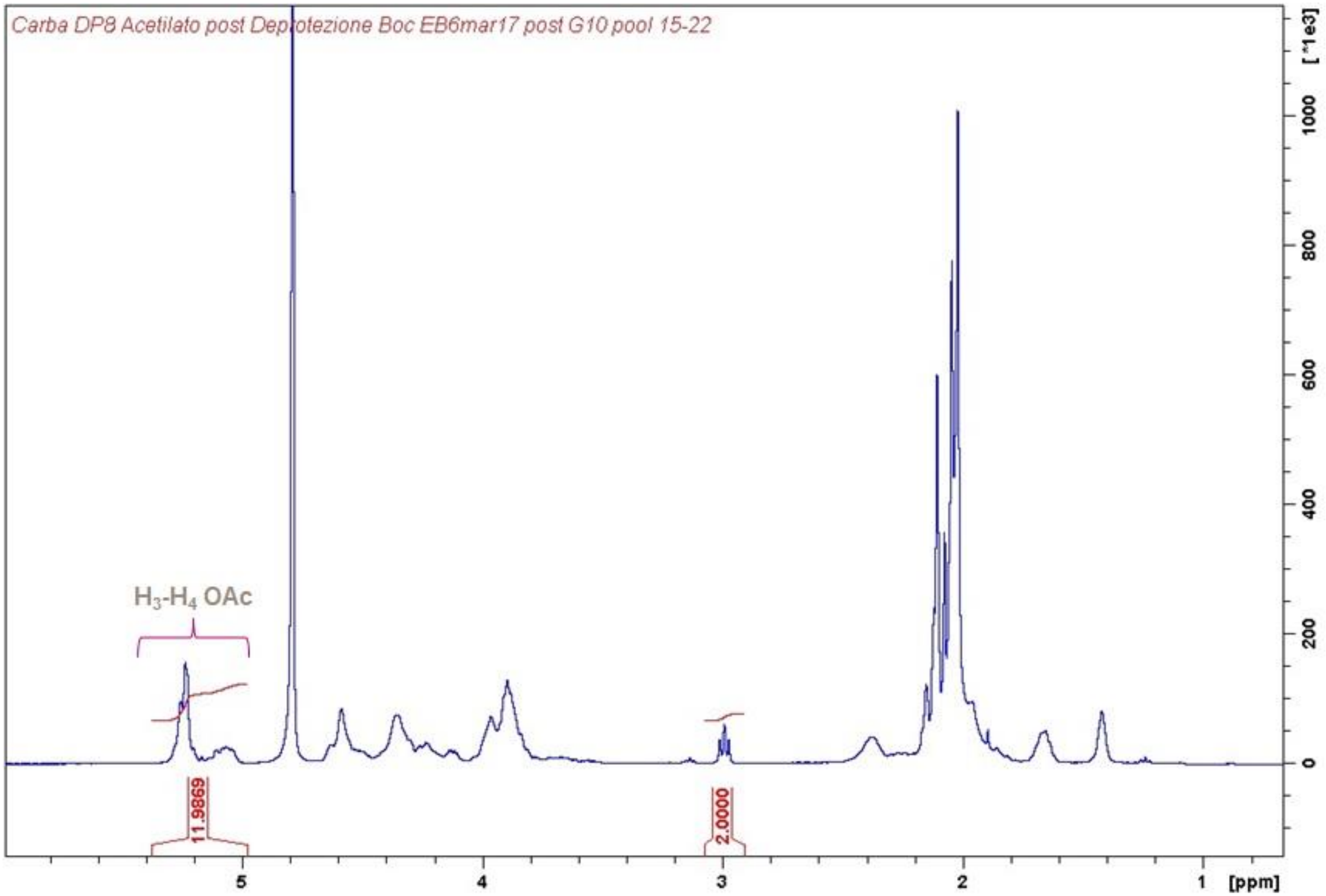

Supplementary Figure 57 ^{1}H NMR (400 MHz, D_2O) of compounds **8a-b**

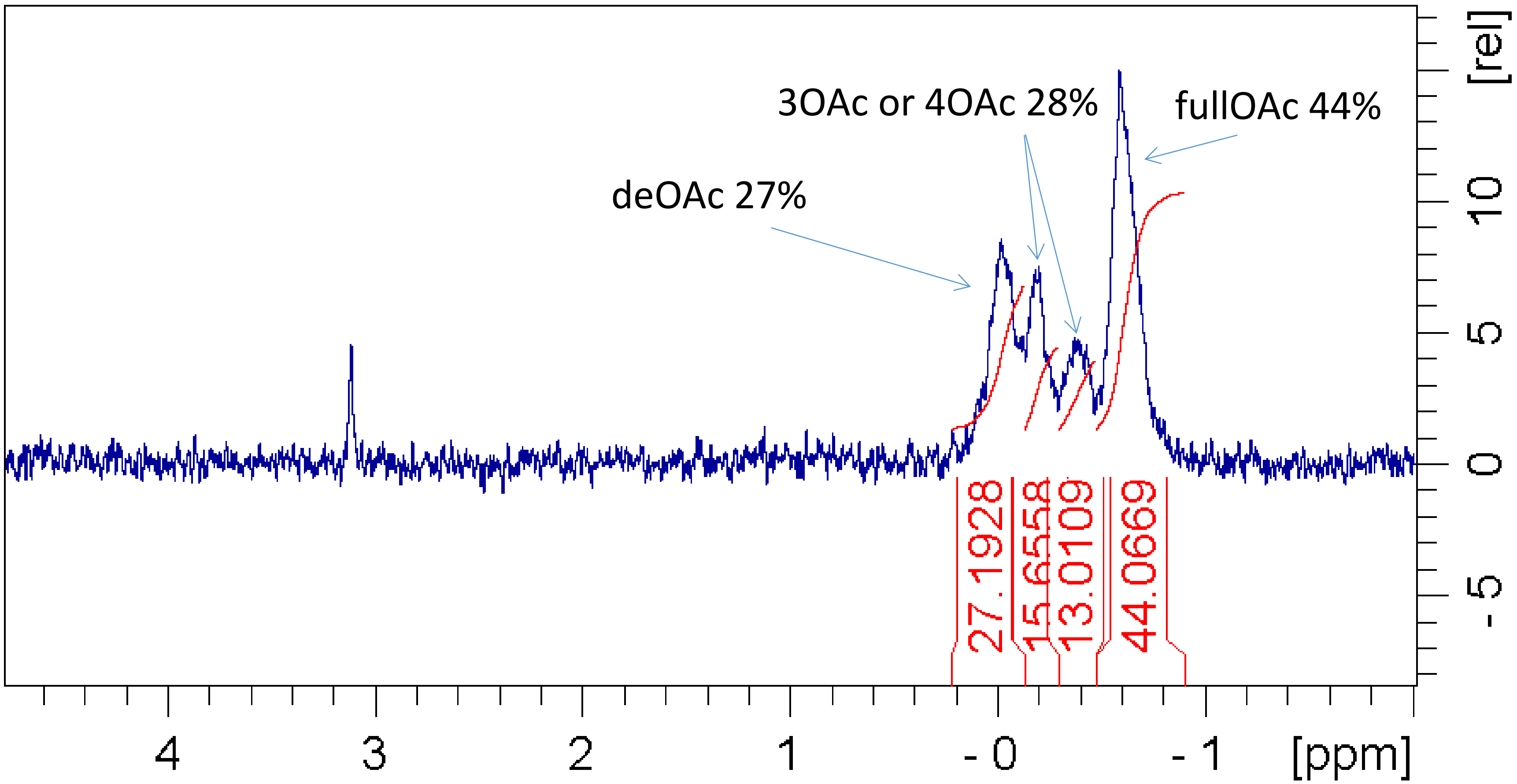

Supplementary Figure 58 ^{31}P NMR (101 MHz, D_2O) compound **8c**
