# Supplementary material for: A stabilized glycomimetic conjugate vaccine inducing protective antibodies against Neisseria meningitidis serogroup A
Source: Nat Commun. 2020 Sep 7;11:4434. doi: 10.1038/s41467-020-18279-x (PMC7477203; doi:10.1038/s41467-020-18279-x)
Supplement: Supplementary file 3 — Reporting Summary [file 41467_2020_18279_MOESM3_ESM.pdf]

nature research

Corresponding author(s): Luigi Lay, Jeroen Codeè, Roberto Adamo

Last updated by author(s): Jul 1, 2020

Reporting Summary

Nature Research wishes to improve the reproducibility of the work that we publish. This form provides structure for consistency and transparency in reporting. For further information on Nature Research policies, see Authors & Referees and the Editorial Policy Checklist .

Statistics

For all statistical analyses, confirm that the following items are present in the figure legend, table legend, main text, or Methods section.

n/a	Confirmed	
☐	☒	The exact sample size (n) for each experimental group/condition, given as a discrete number and unit of measurement
☐	☒	A statement on whether measurements were taken from distinct samples or whether the same sample was measured repeatedly
☐	☒	The statistical test(s) used AND whether they are one- or two-sided<br>*Only common tests should be described solely by name; describe more complex techniques in the Methods section.*
☒	☐	A description of all covariates tested
☒	☐	A description of any assumptions or corrections, such as tests of normality and adjustment for multiple comparisons
☒	☐	A full description of the statistical parameters including central tendency (e.g. means) or other basic estimates (e.g. regression coefficient) AND variation (e.g. standard deviation) or associated estimates of uncertainty (e.g. confidence intervals)
☐	☒	For null hypothesis testing, the test statistic (e.g. F, t, r) with confidence intervals, effect sizes, degrees of freedom and P value noted<br>*Give P values as exact values whenever suitable.*
☒	☐	For Bayesian analysis, information on the choice of priors and Markov chain Monte Carlo settings
☒	☐	For hierarchical and complex designs, identification of the appropriate level for tests and full reporting of outcomes
☒	☐	Estimates of effect sizes (e.g. Cohen's d, Pearson's r), indicating how they were calculated

Our web collection on statistics for biologists contains articles on many of the points above.

Software and code

Policy information about availability of computer code

Data collection	Chromeleon™ 7.2 Chromatography Data System (CDS) Software (SR4 version)
Data analysis	Chromeleon™ 7.2 Chromatography Data System (CDS) Software (SR4 version)

For manuscripts utilizing custom algorithms or software that are central to the research but not yet described in published literature, software must be made available to editors/reviewers. We strongly encourage code deposition in a community repository (e.g. GitHub). See the Nature Research guidelines for submitting code & software for further information.

Data

Policy information about availability of data

All manuscripts must include a data availability statement. This statement should provide the following information, where applicable:

- Accession codes, unique identifiers, or web links for publicly available datasets
- A list of figures that have associated raw data
- A description of any restrictions on data availability

The authors declare that the data supporting the findings of this study are available within the paper and its supplementary information files.

Field-specific reporting

Please select the one below that is the best fit for your research. If you are not sure, read the appropriate sections before making your selection.

☒ Life sciences ☐ Behavioural & social sciences ☐ Ecological, evolutionary & environmental sciences

For a reference copy of the document with all sections, see nature.com/documents/nr-reporting-summary-flat.pdf

Life sciences study design

All studies must disclose on these points even when the disclosure is negative.

Sample size	Group of 10 Balb/C mice were immunized with the vaccine in order to achieve statistically significant. sample calculation was done by nQuery prediction using previous sets of data with similar meningococcal glycoconjugate vaccines. For similar experiments our historical data of antibodies titers in the same animal model showed a standard deviation, on the log 10 transformed scale, of 0.3. Assuming the same variability in our experiment, we predicted a power of 80% to identify a 2.5 fold difference between the geometric means between the two experimental groups using a t test and an alpha level of 0.05.
Data exclusions	No data was excluded from the analysis
Replication	Two separate immunization schedules were conducted and similar results were obtained. Data shown refer to a representative experiment
Randomization	While a formal randomization scheme was not made explicit before the experiment, the animals were randomly allocated to the cages.
Blinding	The study refers to an animal study done in GSK animal care center. Despite a formal protocol for the blinding was not present, the investigators were never in contact with the animals and that the animal facility operators identified the product to be injected with a code that does not allow to know the content of the vials.

Reporting for specific materials, systems and methods

We require information from authors about some types of materials, experimental systems and methods used in many studies. Here, indicate whether each material, system or method listed is relevant to your study. If you are not sure if a list item applies to your research, read the appropriate section before selecting a response.

Materials & experimental systems

n/a	Involved in the study	
☐	☒	Antibodies
☒	☐	Eukaryotic cell lines
☒	☐	Palaeontology
☐	☒	Animals and other organisms
☐	☒	Human research participants
☒	☐	Clinical data

Methods

n/a	Involved in the study	
☒	☐	ChIP-seq
☒	☐	Flow cytometry
☒	☐	MRI-based neuroimaging

Antibodies

Antibodies used	monoclonal anti meningococcal serogroup A polysaccharide JW-A1 GSK proprietary and purified by Areta; the same mAb in recombinant form (GSS2) was produced by Biovest
Validation	the mAb has been produced by Areta and validated internally in GSK through a competitive ELISA for vaccine lots release

Animals and other organisms

Policy information about studies involving animals; ARRIVE guidelines recommended for reporting animal research

Laboratory animals	BALB/c female mice, 5 weeks old, housed under specific pathogen-free conditions at the GSK Vaccines Animal Resource Centre
Wild animals	the study inolved use of commercial rabbit complement
Field-collected samples	field-collected samples were not involved
Ethics oversight	Experiments were undertaken in accordance with the regulations of the Directive 2010/63/EU and GSK ethical guidelines, under approval of the Italian Ministry of Health.

Note that full information on the approval of the study protocol must also be provided in the manuscript.

Human research participants

Policy information about studies involving human research participants

Population characteristics	*Describe the covariate-relevant population characteristics of the human research participants (e.g. age, gender, genotypic information, past and current diagnosis and treatment categories). If you filled out the behavioural & social sciences study design questions and have nothing to add here, write "See above."*

Recruitment	*Describe how participants were recruited. Outline any potential self-selection bias or other biases that may be present and how these are likely to impact results.*
Ethics oversight	*Identify the organization(s) that approved the study protocol.*

Note that full information on the approval of the study protocol must also be provided in the manuscript.
