# Supplementary material for: A stabilized glycomimetic conjugate vaccine inducing protective antibodies against Neisseria meningitidis serogroup A
Source: Nat Commun. 2020 Sep 7;11:4434. doi: 10.1038/s41467-020-18279-x (PMC7477203; doi:10.1038/s41467-020-18279-x)
Supplement: Supplementary file 2 — Peer Review File [file 41467_2020_18279_MOESM2_ESM.pdf]

Editorial Notes: This manuscript has been previously reviewed at another journal that is not operating a transparent peer review scheme. The manuscript was considered suitable for publication without further review at *Nature Communications*.
